# Leveraging the One Health concept for arsenic sustainability

**DOI:** 10.1016/j.eehl.2024.02.006

**Published:** 2024-03-07

**Authors:** Yujie Huang, Qi Miao, Raymond W.M. Kwong, Dapeng Zhang, Yuchuan Fan, Ming Zhou, Xiliang Yan, Jianbo Jia, Bing Yan, Chengjun Li

**Affiliations:** a Institute of Environmental Research at Greater Bay Area, Guangzhou University, Guangzhou 510006, China; b Department of Biology, York University, Toronto, ON M3J 1P3, Canada; c Department of Soil, Water, and Ecosystem Sciences, University of Florida-IFAS, Gainesville, FL 32603, USA; d Centre for Catalysis and Clean Energy, Gold Coast Campus, Griffith University, QLD 4222, Australia; e College of Animal Science, South China Agricultural University, Guangzhou 510642, China

**Keywords:** Arsenic, Framework, Sustainability, Environmental health, One Health

## Abstract

Arsenic (As) is a naturally occurring chemical element widely distributed in the Earth's crust. Human activities have significantly altered As presence in the environment, posing significant threats to the biota as well as human health. The environmental fates and adverse outcomes of As of various species have been extensively studied in the past few decades. It is imperative to summarize these advances as a whole to provide more profound insights into the As cycle for sustainable development. Embracing the One Health concept, we systematically reviewed previous studies in this work and explored the following three fundamental questions, i.e., what the trends and associated changes are in As contamination, how living organisms interact and cope with As contamination, and most importantly what to do to achieve a sustainable future with As. By focusing on one critical question in each section, this review aims to provide a full picture of the complexity of environmental As. To tackle the significant research challenges and gaps in As pollution and mitigation, we further proposed a One Health framework with potential coping strategies, guiding a coordinated agenda on dealing with legacy As in the environment and ensuring a sustainable As future.

## 1 Introduction

The presence of arsenic (As) can be found in almost all environmental substrates [1], with its elemental form being far rarer than its compound counterparts [2]. Arsenic has been used for diverse purposes by humans, such as in medicines and semiconductor industries [1,3]. Environmental As is present in either inorganic or organic forms, with the most common oxidation states of As in the environment being arsenite (As^III^) and arsenate (As^V^) under anoxic and oxic conditions, respectively [4]. Inorganic As (iAs) is well-known in the mainstream and scientific literature since it has been documented as a human carcinogen [5]. By contrast, organic As (oAs) compounds, such as monomethylarsonic acid (MMA) and dimethylarsinic acid (DMA), have relatively low toxicity [6].

Mounting evidence has shown that As contamination is now widespread, affecting not only groundwater but also farmland, causing severe problems [[7], [8], [9]]. For example, As-contaminated groundwater and drinking water have been frequently reported worldwide, especially after poisoning incidents in the late 1980s in India and Bangladesh due to the consumption of high-As well water [10]. Arsenic accumulation in food, particularly grains like rice, and seafood is also becoming a problem of great concern [11]. This is because grains have been the staple food globally for a long time, while seafood has been gaining popularity around the world [12,13]. It should be noted that seafood has the highest As levels, mainly oAs, while grains, especially rice, contain primarily iAs, a carcinogen well-known to the general public and frequently reported in scientific literature [12,13].

Prolonged exposure to As-contaminated water and food through dietary intake or usage of As-containing industrial products poses significant health hazards to animals and humans, including but not limited to dysbiosis, obesity, metabolic syndrome, chronic kidney and heart diseases, cancer, and maternal and fetal complications [5,14]. From the viewpoint of ecosystems, when As accumulates persistently in the environment, it not only impacts biological organisms but also alters various physical and chemical properties, including but not limited to pH and organic matter content within the environment. These changes, in turn, can significantly modify the growth conditions for living organisms. This could ultimately lead to biodiversity loss and disrupted ecological equilibrium in affected regions [15].

To address this challenge, numerous methods have been developed or proposed to mitigate As contamination and health risks associated with As. Numerous As sorption, particularly adsorption, and removal methods have been proposed and applied as possible solutions to As contamination in the environment [16]. However, there are clear disparities between regions with dense populations, especially in low- and middle-income countries (LMICs) where access to mitigation-relevant resources, including knowledge, facilities, and techniques, is lacking [17,18]. Such disparities could amplify As-induced risks and subsequently compromise global efforts to tackle As contamination.

In this study, we focus on the As cycle and its subsequent impacts on the environment as a whole, and explore three key questions: 1) What are the trends and associated changes in As contamination? 2) How do living organisms interact with As contamination? 3) What can be done to achieve As sustainability? By exploring these three questions, we propose a framework with the One Health concept [19], calling for a unified agenda on As contamination. We believe such a framework can help achieve a sustainable and liveable future for both humans and wildlife in the context of As contamination.

## 2 A shifting trend in arsenic contamination

Human activities are the major contributors to the accumulation of As and its compounds in the environment, while natural sources of As, such as volcanic activity, generally account for a relatively small proportion of the total amount of environmental As. Anthropogenic sources of As emissions include fossil fuel and coal combustion, metal mining and smelting, and the intentional use of As in various products during production and everyday life [20]. Changes in these emissions and associated patterns in the past few decades have driven a shifting trend in the hotspots of As contamination in the environment, from groundwater to the soil, and to all possible environments. However, it is noteworthy that As contamination in groundwater for drinking and irrigation results from a combination of anthropogenic and natural causes.

### 2.1 Arsenic in groundwater—a persistent problem

**Fig. 1:**
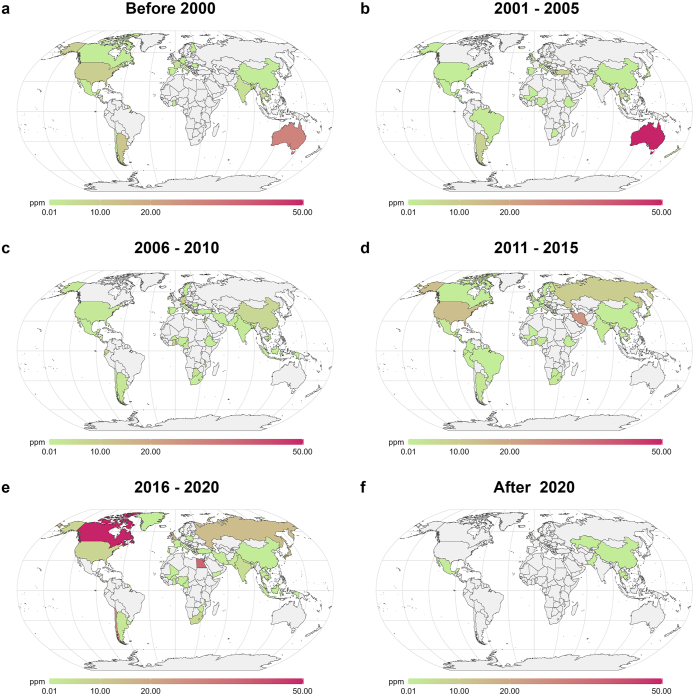
Global average concentration levels (ppm) of As in groundwater during each time interval. The concentration in each map plot is presented as the average concentrations reported in each study published during each time interval for a specific country/region. The groundwater with high As levels is mainly distributed in Southeast Asia and the Mediterranean region, with some extremely high values occasionally detected in Canada, Brazil, and Australia. To make plotting aesthetic, we capped the maximum value at 50 ppm in the map for these extreme cases. Data were collected from 464 qualified publications as per the criteria described in Supplementary Methods. Briefly, to reflect the worst-case scenario of groundwater contamination situation in each region, the highest concentration value in a single study was collected. Then, data points within the same time intervals of the same region were averaged to represent the value within the same region in that specific interval. Details on publication and data collection can be found in Tables S1–S2 and Supplementary References.

The contamination of groundwater by As is prevalent. According to our comprehensive data analysis (see details in Supplementary Methods and Tables S1–S4), a total number of 73 countries during the past four decades reported As contamination problems with their groundwater (Fig. 1; Tables S1–S2). In most cases, natural sources are the primary causes, while mining and industrial activities contribute to only a small portion of As pollution [21].

The As concentration in groundwater in Southeast Asia has maintained a high level for a long time (Fig. 1a–f), reaching 2,629 ppb in Nepal, 517 ppb in Bangladesh, and 1,891 ppb in India (see details in Table S1). China also faces challenges related to As contamination in groundwater, with varying concentrations observed across different years and some years experiencing notably higher concentrations (Fig. 1; Table S1). It should be noted that occasionally, Australia, Brazil, and the USA also report extremely high levels of As in groundwater (Fig. 1b, e; see details and reference information in Table S1). Overall, a significant gap exists between As levels and the 10 ppb standard set (i.e., 0.01 ppm as shown in Fig. 1) by the World Health Organization (WHO) [17]. However, it should be noted that these reported As concentration levels might be misleading or at least biased to some extent [22]. Historically, the detection of As in groundwater has been limited by testing costs, often occurring only after health effects are diagnosed. Indeed, this phenomenon may be a contributing factor to substantial variations in As concentrations observed in certain countries and regions. Previous studies have laid a foundation for predicting As concentrations in groundwater by examining the dissolution form of As released from sediment into groundwater and its movement. Predictive models based on these studies can play a crucial role in areas where the impact of As has not yet been determined [23].

Groundwater in arid or semi-arid inland or enclosed basins is frequently contaminated with As [24]. Historically, poorly flushed aquifers allowed As to be released from sediments and accumulate in the groundwater [25]. Studies have shown that As mobilization is associated with the recent inflow of carbon, with organic carbon or its degradation products quickly mobilizing As and limiting the sorption of As despite the presence of oxidants [26]. Such speculations were confirmed by the fact that carbonate and ferrous iron (Fe^II^) could affect the mobility of As since high As concentrations in subsurface waters were due to the reductive dissolution of hydrous Fe oxides and/or the release of adsorbed As [27]. One year later, a study on the mechanism of As release and source(s) in a small part of a watershed in the Murshidabad district of West Bengal further showed that the main stage of As release into groundwater was related to the bacterial reduction of ferric iron (Fe^III^) to Fe^II^ [28]. Since then, studies have focused on the importance of microbial processes, showing that microbes can mediate the release of As into groundwater under reducing conditions [29]. It is worth noting that As^III^, methyl arsenite, and dimethyl arsenate are all forms of As that can dissolve in water, with As^III^ being more easily dissolved and transported than As^V^ [30].

However, it is confusing that ferric (hydr)oxides appeared to dominate the partitioning of As at the near surface yet had a limited impact at aquifer depths where wells extracted groundwater with high As concentrations [26]. This puzzle was solved to some extent by Polizzotto et al. [31], who presented a sequence of evidence and suggested that As may be released at the near surface and then transported to depth. Three years later, they published a follow-up study explaining how such a mechanism works [32]. Using hydrologic and (bio)geochemical measurements, they showed that As is released from near-surface, river-derived sediments and transported, on a centennial timescale, through the underlying aquifer back to the river. This release process is disturbed by anthropogenic activities such as groundwater pumping and upstream dam installations, thus influencing groundwater As concentrations [32]. Such a model has shown great potential in identifying As mobilization and subsequent contamination, significantly advancing our understanding of As mobilization in different environments.

Overall, understanding the sources of As in groundwater, either for drinking or irrigation, and its mobilization between different environments is critical for addressing As contamination. According to statistics from 50 countries, at least 140 million people are exposed to drinking water with As levels exceeding the guideline value set by WHO [17,33]. At the same time, many more regions have not yet tested their drinking water due to technical and economic limitations [34]. As demonstrated in our collected data (Table S2; Fig. 1), most countries except for a few in the African continent, the world's second-largest and second-most-populous continent, have not been mentioned in previous studies. This should be one of the focal points of future As studies, which is of great importance in safeguarding access to clean drinking water and achieving the UN sustainable development goals (SDGs) by 2030.

### 2.2 Arsenic in the soil–emerging hotspots

Unlike groundwater, where As is mainly emitted from natural sources, anthropogenic mining activities and pollutant emissions from As pesticide production enterprises are the most important sources of As in soil [35,36]. iAs compounds exist primarily as AsO_4_^3−^ and As sulfide (As_x_S_y_) in the environment [21,36].

**Fig. 2:**
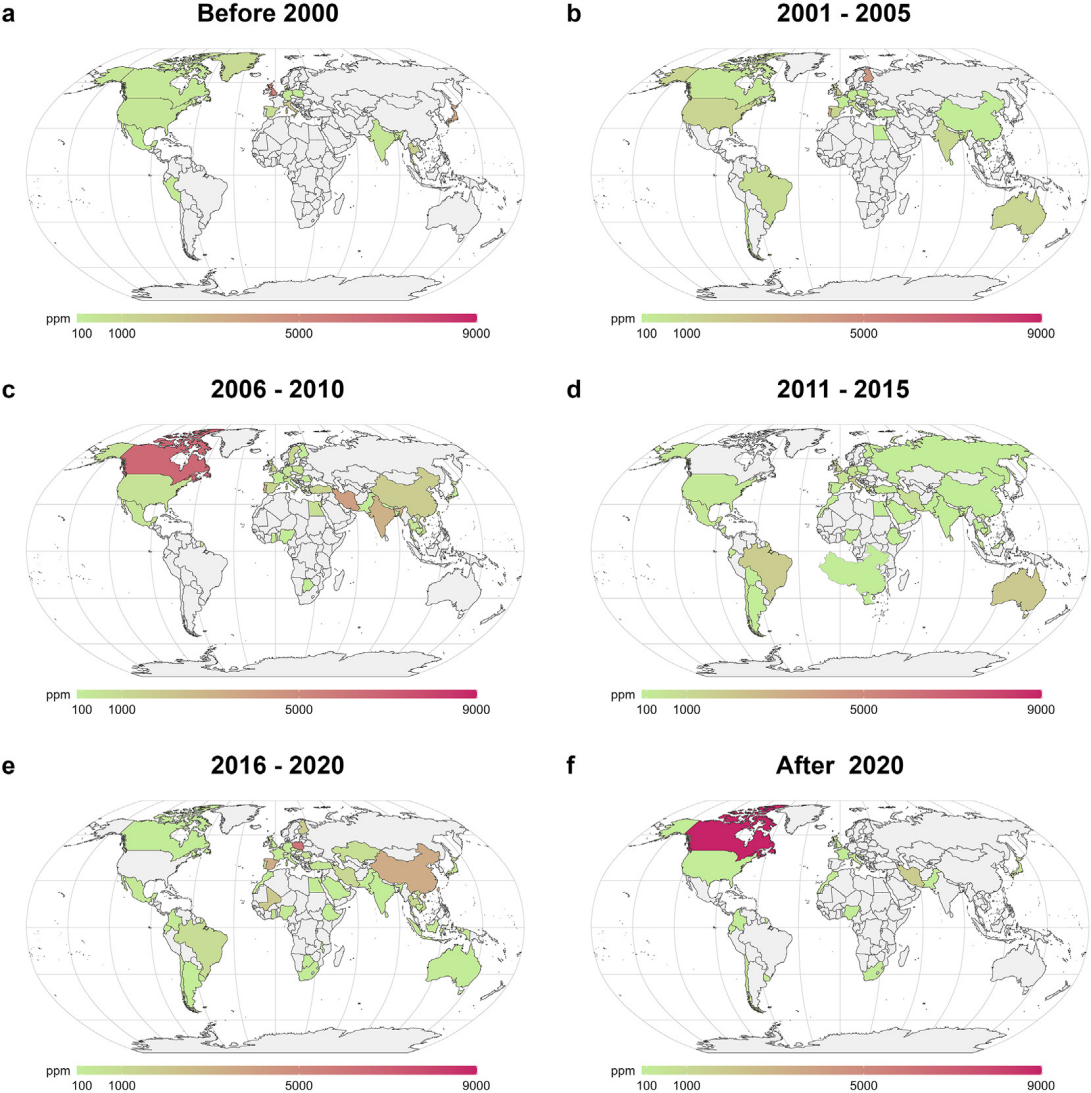
Global average concentration levels of As (ppm) in the soil during each time interval. High As levels in the soil are mainly caused by anthropogenic activities, such as mining and war-induced chemicals and As residues. Arsenic pollution caused by anthropogenic activities far surpasses that resulting from natural causes. Unless the source of As emissions is effectively controlled, significant efforts will be required for later-stage mitigation strategies. Soil pollution by As has drawn an increasing amount of attention since the 2010s, as shown in **a–f**. Data were collected from 464 qualified publications as per the criteria described in Supplementary Methods. Briefly, to reflect the worst-case scenario of groundwater contamination situation in each region, the highest concentration value in a single study was collected. Then, data points within the same time intervals of the same region were averaged to represent the value within the same region in that specific interval. Details on publication and data collection can be found in Tables S3–S4 and Supplementary References.

Since the First Industrial Revolution, mining activities have discharged more than 22,000 tons of As into the environment as As_2_O_3_ [37,38], with the number of countries reporting soil As contamination being 61 (Fig. 2), compared to 73 for groundwater contamination (Fig. 1). By contrast, the crustal abundance of As is relatively low, accounting for only 0.0001% [39]. By plotting these two datasets, our analysis further demonstrates that research topics of As contamination in the soil have been emerging as the hotspot (Fig. S1; Tables S3–S4). Specifically, the number of countries reporting As soil pollution issues has risen from 14 before 2000 (Fig. 2a) to 35 during 2006–2010 (Fig. 2c), and 44 during 2011–2015 (Fig. 2d), surpassing that of groundwater contamination (Fig. S1; Tables S1–S4).

The As concentration in the soil near the mining area can reach up to 30,000 ppm [40], but it decreases rapidly with the increase of the distance from the mining area. In the mining area, As can be released into the atmosphere as As_2_O_3_, adsorbed on particles, and then transported to the ground through the wind, and will undergo dry and wet sedimentation [41]. Due to anthropogenic factors, the As content in soil is generally far higher than that in groundwater [42], except for some extreme cases, and the As concentration in groundwater is often affected by high As levels in soil [43] (Fig. 2; Table S3). This is probably the reason why there is an overlap between countries experiencing groundwater contamination and countries with soil pollution, as shown in Fig. 1, Fig. 2, as well as Tables S1–S4.

It should be noted that changes in Earth's environment, such as the impact of carbon sequestration on soil and the use of phosphorus fertilizers, have a significant impact on soil As activity. Specifically, the composition of soil, particularly its organic carbon content and dissolved organic matter, significantly influences As concentrations. Soils rich in organic carbon tend to exhibit higher As levels, while increased dissolved organic matter hinders As adsorption onto other soil components [44,45]. The transformation and migration of As are intricately linked to soil conditions and microbial communities. In oxidative conditions, microorganisms are more prone to converting iAs into organic forms. Conversely, in anaerobic conditions, As is more likely to undergo reduction, forming diverse As compounds (see *Section* 3.1). Meanwhile, stable phases, such as soil sulfides, heavy metal oxides, and As hydroxides, contribute to the complexity of As dynamics in soil, necessitating a comprehensive understanding of soil composition and As phases for effective study [36]. Meanwhile, the application of phosphorus fertilizer in agricultural production may also elevate the activity of As in the soil, leading to an increased As accumulation in crops and, subsequently, in the human body [46,47]. With the intensification of agricultural production activities and the anticipated widespread use of carbon dioxide and phosphorus fertilizers, the potential for As enrichment in crops becomes a concern. Addressing this requires exploring alternative solutions to support agricultural production while mitigating the risk of As accumulation in crops (see *Section* 3.3).

In summary, while natural As in drinking water is the primary contributor to As pollution in Southeast Asia, particularly in water bodies, anthropogenic activities play an increasingly important role in soil As contamination. However, scientists tend to focus on the impact of natural sources of As while overlooking anthropogenic emissions [39]. Moreover, many regions, especially LMICs have not been frequently covered in previous studies, among which Africa is worth special attention. Despite being the second most populous continent after Asia, Africa is facing a staggering research void in addressing the critical issue of As pollution [48]. This could be further compounded by the fact that many LMICs have been experiencing increasing pressure on food security and thus foreseeable intensified agriculture production [49,50]. Urgent and unwavering attention is imperative to improve understanding of As contamination in drinking water and soils in these areas.

## 3 Organisms' interactions with arsenic: From tiny to big

**Fig. 3:**
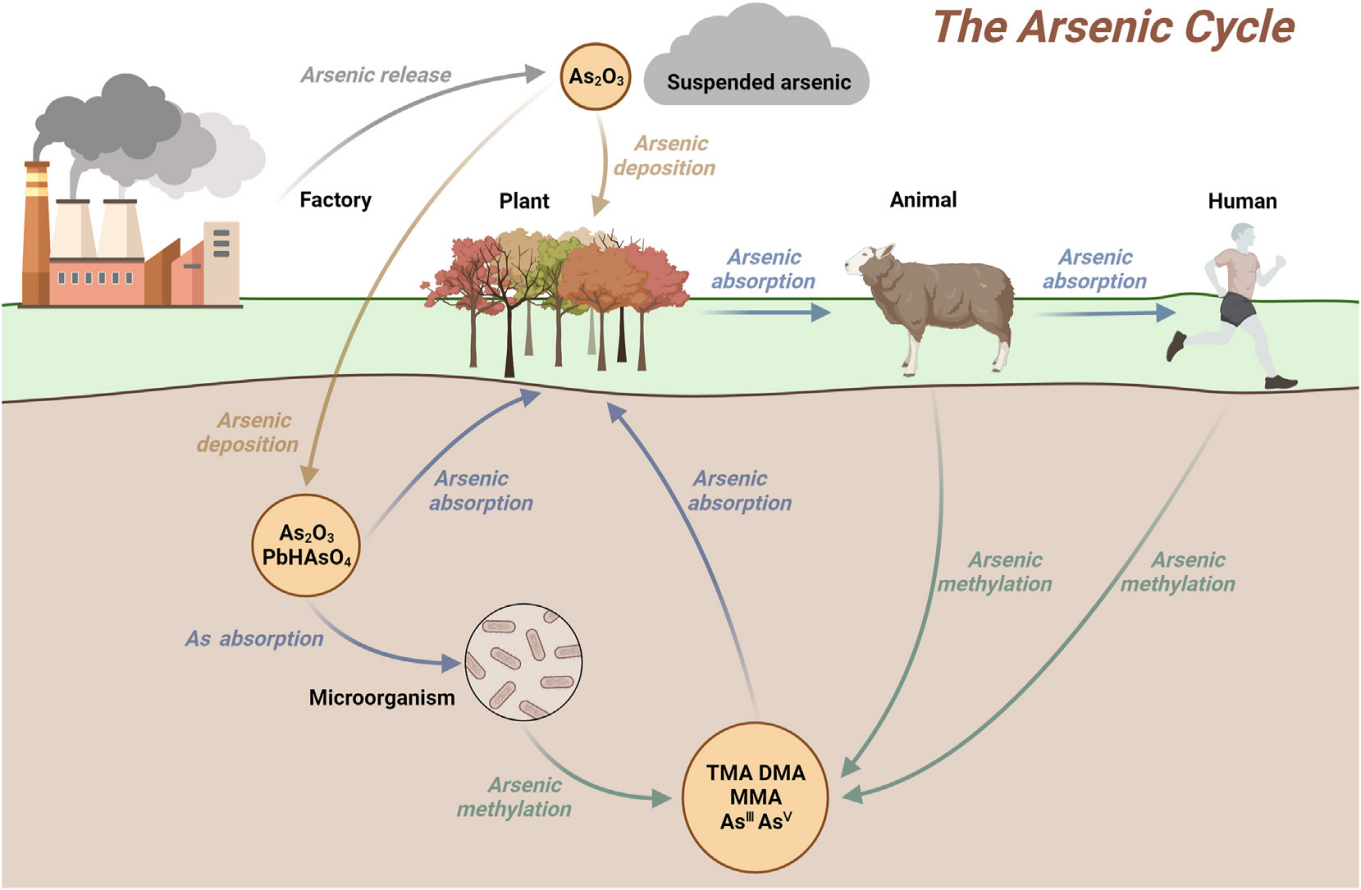
The major roles of organisms in the As cycle. Anthropogenic mining and industrial activities release As into the atmosphere as As_2_O_3_. This airborne As can travel significant distances in the atmosphere before settling on leaves and soil surfaces. Microbes and plants can take up As in the soil. When it enters plants, As accumulates and becomes increasingly concentrated as it moves along the food chain, eventually posing a threat to human health. After ingestion, organisms often metabolize and transform As, leading to the release of MA and iAs back into the environment. Credit: this figure was drawn using Biorender.

In different environments, Arsenic can readily undergo metabolic conversions mediated by microorganisms, plants, and animals, eventually impacting human health via environmental exposures and food chains (Fig. 3) [51]. Arsenic is primarily ingested by microbes in an inorganic form. Once absorbed, it undergoes processes such as metabolism, transformation, storage, and release into the environment as oAs. The mechanism of As entry into plants mirrors that of microbes [39], but notable variations exist between species. Exploring the interactions between As and living organisms in the environment as a whole, therefore, is of great importance to advance our understanding of As toxicity and subsequently mitigate its environmental impacts from a One Health perspective.

### 3.1 Microbes as the starting point

Microorganisms, comprising archaea, bacteria, and fungi, are the oldest living organisms. Among them, bacteria are the focal point of As research due to their crucial role in the biogeochemical cycle of As. In bacteria, As^V^ passes through the inorganic phosphate transporter Pit (a transmembrane protein) and the phosphate-specific transport protein Pst (an ABC periplasmic transport protein). Soluble As is absorbed by aquaporin in the form of As hydroxide, while insoluble As is absorbed by glycerol uptake facilitator, GlpF, in the form of As oxide [52].

There are four major pathways of As metabolism in microorganisms: reduction, oxidation, methylation, and demethylation (Fig. 3). For example, certain marine anaerobic bacterium *Serratia* can convert As^V^ to As^III^, and then to methylsulfite [53]. Similarly, bacteria in rat intestines are capable of reducing As^V^ [54]. As^V^ reductase is an enzyme specifically for As^V^ reduction [55]. Under anoxic conditions, microbes reduce As^V^ as a terminal electron acceptor of anaerobic respiration to As^III^, and use other organic compounds, such as glucose, as electron donors for As^V^ reduction. Under aerobic conditions, the reduction reaction of As^V^ can be inhibited.

There are, however, some special circumstances. For example, the dissimilatory reduction in *Bacillus* sp. SF-1 strain and *Citrobacter* sp. NC-1 strain is not part of the As-resistant system. As^III^ oxidation involves two genes encoding the large subunit of As^III^ oxidase, i.e., *aioA* and *aioB*, where *aioA* oxidizes As^III^ to As^V^. In this oxidation mechanism, Molybdenum (Mo) acts as an electron acceptor, undergoing reduction from Mo^VI^ to Mo^IV^ [56]. In *Rhizobium* sp., the presence of Cytochrome C552 is crucial for As^III^ oxidation. After the generation of As^V^, the reduced arsenite oxidase (Aio) assumes a functional oxidized form through two consecutive oxidation steps with the assistance of Cytochrome C552. The methylated As in bacteria becomes volatile and diffuses out from the cell in trimethylarsine (TMA) oxide forms in a series of methylation reactions (three-step process) by a highly diversified As methyltransferase (arsM) enzyme [57].

Considering the relatively rich As content in the early oceans, the ability of microbes to absorb and metabolize As may have emerged in the oceans billions of years ago [58]. Although As is highly toxic to prokaryotes and eukaryotes, it can still be used as a nutrient for certain anaerobic bacteria [39]. Therefore, the presence of channels in microbes that allow As to enter cells can be reasonably explained from the evolutionary point of view. For example, apart from its role in redox reactions to generate energy for anaerobic bacteria, As is also involved in other vital processes in anaerobic bacteria [59]. However, a noticeable disparity exists between the advancements in research regarding their practical applications and our understanding of the underlying mechanisms. Future studies should pay more attention to how to utilize microbes, the most widespread organisms on Earth, more efficiently to mitigate the impact of As on all living creatures.

### 3.2 Plants as the amplifiers

Plants accumulate As present in the soil, water, and air, potentially amplifying the impacts of As along the food chain. The root system is the most important organ for material exchange between plants and surrounding environments. It is also responsible for filtering and storing harmful substances in plants. Generally, the root system is the main storage site for As, resulting in less As being transferred to stems, leaves, and fruits [60]. Although many harmful substances are isolated in plant roots, some will still enter subsequent tissues. With the progression of human industrialization, an increasing influx of As finds its way into plants via contaminated soil and water sources [61]. Consequently, As contamination can extend to various elements of our environment, including trees, grasslands, and even foods, leading to a profound and detrimental impact on human health.

**Fig. 4:**
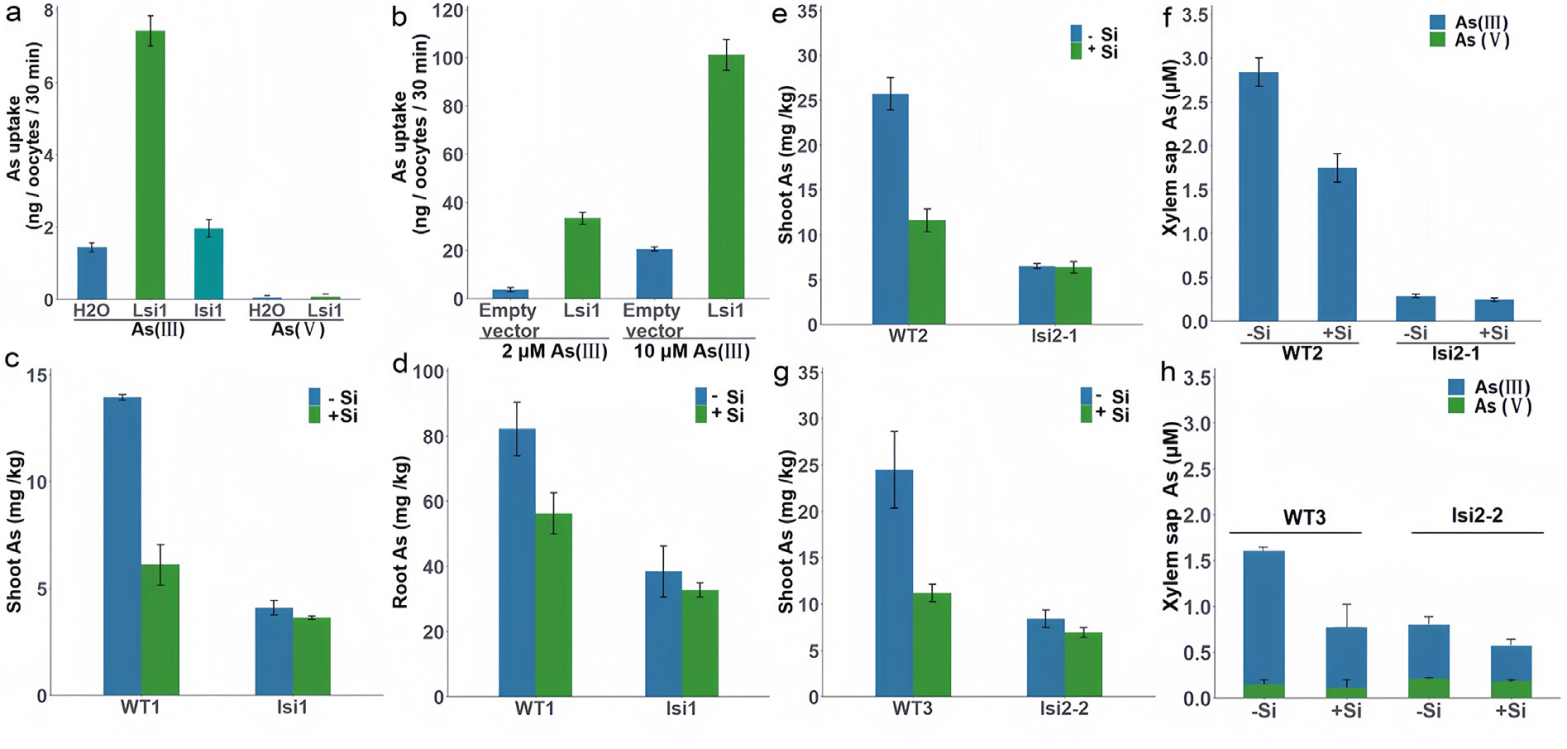
Arsenic transport activities of *Lsi1* and *Lsi2*. (a) Arsenic uptake by oocytes expressing *Lsi1* or mutant *lsi1*, with H_2_O as the control. Oocytes were exposed to 100 μM As^III^ or As^V^ for 30 min. (b) Arsenic uptake by the yeast mutant (*acr3*) expressing *Lsi1* or the empty vector. Yeast was exposed to 2 or 10 μM As^III^ for 30 min. (c and d) Concentration of As in shoots (c) and roots (d) of the wild-type rice (WT1 = cv. Oochikara) and the mutant *lsi1*. Two-week-old seedlings were exposed to a nutrient solution containing 2 μM As^III^ with or without 0.5 mM silicic acid for 1 day. (e) and (g) show concentration of As in the shoots of the two mutants (*lsi2-1* and *lsi2-2*) and their wild-type rice (WT2 = cv. T-65, WT3 = cv. Koshihikari) where two-week-old seedlings of four lines were exposed to 2 μM As^III^ with or without 0.5 mM silicic acid for 7 days. (f) and (h) show the concentrations of As^III^ and As^V^ in the xylem sap collected from the wild-type rice and *lsi2-1* and *lsi2-2* mutants after seedlings were exposed to 5 μM As^III^ with or without 0.5 mM silicic acid for 1 day. (e–h) Two-week-old seedlings of the wild-type rice (cv. Nipponbare) were exposed to 0 or 5 μM As^III^ for 7 days, and *Lsi2* expression was quantified by RT-PCR. Data in (a)–(d) are means ± SD (n = 3). Adapted with permission from ref. [62]. Copyright (2008) Proceedings of the National Academy of Sciences of the United States of America.

The form of As is a critical factor that affects its absorption efficiency in different environments, e.g., a higher absorption efficiency in anaerobic environments for As^III^, and in aerobic environments for As^V^ [63]. In the actual environment, factors such as microbes in soil or redox potential will lead to revisable transformation between As^V^ and As^III^. For As^V^, many phosphorus transporters in plants have been characterized [64], especially in *Arabidopsis thaliana* where two transporters Pht1;1 and Pht1;4 play a significant role in phosphorus acquisition [65]. For As^III^, rice (*Oryza sativa*) is the most frequently studied model species [66]. As^III^ is the dominant As species in paddy fields where flooding facilitates the mobilization of As into the soil solution, thus enhancing As bioavailability to rice plants [67]. Rice is capable of rapidly taking up As^III^ from an external medium, which could be inhibited by glycerol and antimonite but not by phosphate due to the involvement of aquaporin channels in plant roots that can mediate As^III^ influx [62]. The major pathway for the entry of As^III^ into rice roots is via OsNIP2;1, also known as *Lsi1* because of its primary function as a silicon (Si) transporter [68] (Fig. 4). *Lsi1* is responsible for transferring As^III^ from root cells to the xylem. Its counterpart, *Lsi2*, is an efflux transporter. The transport of silicon and As^III^ results from the synergy between *Lsi1* and *Lsi2* transporters. Meanwhile, iAs may also be absorbed by *Lsi1* [69]. The As absorption by leaves also accounts for a part of the total As absorption of plants, mainly through the cutin cracks and stomata [70]. While DMA transfer efficiency in rice is higher than iAs, As^V^ is easier to move in the phloem and xylem. At the same time, As^III^ remained in the endosperm vascular, but DMA dispersed in the endosperm outer granules and entered the endosperm [71].

After absorption, As^V^ is effectively reduced to As^III^ in cells of most plant species [72]. For example, the rice genome contains two *acr2*-like genes *OsACR2.1* and *OsACR2.2*. The purified product of the gene can reduce As^V^ to As^III^ *in vitro*, and both have phosphatase activity. After knocking out the *AtACR2* gene in Arabidopsis, As^V^ could not be effectively reduced to As^III^ and translocated to the aboveground part of the plant as a phosphate substitute [73]. As for the methylation of iAs into oAs, it is very rare in plants despite being very common in microbes [74]. These various forms of As are mainly stored directly in vacuoles, while the rest participate in the life activities of plant cells and undergo the process of oxidation, reduction, and methylation. Nevertheless, researchers mainly focus on finding hyperaccumulating plants for *in situ* remediation of contaminated land, while little attention has been paid to advancing understanding and techniques of reducing the influx of As and other heavy metals from farmland to crops, which may have a greater impact on human health (see details in *Section* 4) [75].

### 3.3 Animals and humans as the major recipients

The fates of As compounds have been extensively studied in animals of terrestrial ecosystems due to their high trophic levels (Fig. 3). The main As-exposure routes for animals are drinking As-containing water and feeding on As-contaminated terrestrial or aquatic foodstuffs [76]. For humans, social and behavioral differences determine the routes of As exposure. For example, As-containing particles are the main way of intake in occupational environments, with significant intake and skin exposure under specific circumstances [30]. Exposure to As is also subject to cultural and dietary differences since populations in different regions may have completely different diets, e.g., various combination of rice and wheat as well as their related products, thus suffering from different degrees of As contamination. Take China as an example. Populations in Northern China mostly consume wheat-based products, whereas the staple food in Southern China is rice [77]. Since foodstuffs, especially rice and wheat, contain different levels of As, the extent to which populations with different diets suffer from As contamination in foodstuffs may vary between/within regions. Nowadays, trading activities may further complicate such a situation. Consumers nowadays have been provided with many more choices of rice and wheat varieties from different sources, either regionally, globally, or both, thanks to the development of global/regional trade [2]. As a result, consumers are likely to be affected by As contamination in other regions as well due to the consumption of imported rice- and wheat-based products [78].

In vertebrates, As^III^ is generally taken up via aquaglyceroporins [a subset of the aquaporin (AQPs) family of proteins], similar to that in prokaryotes and eukaryotes. Members of AQPs, especially AQP7 in the kidney and AQP9 in the liver, facilitate the uptake of trivalent iAs, whereas AQP3 shows limited ability to transport As^III^. This observation is consistent with the finding that As is largely accumulated in the liver in different mammals, including humans [79]. Therefore, AQP9 may play an important role in the simultaneous influx of As^III^ from blood to the liver and the efflux of As metabolite MMA^III^ from the liver to blood [80]. As^V^ is transported into cells by phosphate transporters. In mammals, some Na^+^/Pi-cotransporters are shown to facilitate phosphate in an As^V^-sensitive manner, suggesting that they might use As^V^ as a substrate [81]. A more recent study suggests that rat Type II phosphate transporters can transport As^V^ in an oocyte expression system [82].

It was originally thought that humans and mammals share the same As metabolic pathways, but numerous studies have suggested that mammals are often less sensitive than humans to As, which may be due to diverse As methyltransferase in mammals [83]. Low trophic organisms, often interacting more with the soil, are the first to reflect changes in the concentration of heavy metals in the soil. Once As enters the low trophic organism's body, it is excreted from the body or transferred to the next trophic level. The gastrointestinal tract is the primary pathway for As ingress into the human body. In animals and humans [84], As^V^ can be reduced to As^III^ in the blood. The resulting As^III^ mainly accumulates in the liver and is metabolized by a sequence of reduction and oxidative methylation [85]. Specifically, iAs undergoes methylation to generate MMA^III^ and DMA^III^ [79]. Then, As is excreted mostly by urine as a mixture of metabolites and the remaining As in foodstuffs [86]. Further studies have suggested that in certain species, but not humans, DMA can be converted into trimethylarsine oxide (TMAO) during oxidative methylation [87]. At present, there are also studies indicating that the methylation of iAs is actually the activation mode of As, rather than the detoxification mode of As in organisms. It can be used as a carcinogen but can be excreted by animal and human metabolic waste [36].

In the metabolic process of humans and animals, As may undergo various intermediate transformations, leading to the formation of end products and intermediates that can be excreted from the body [30]. Specifically, iAs compounds in the environment can be transformed into oAs compounds after various biological transformations, and they may be transformed again into iAs compounds after photolysis or biodegradation by microbes [88]. Therefore, once As and its compounds are released from geology to the external environment, they will undergo a complex biogeochemical cycle in the environment. In this process, As will exist for a long time and continue to accumulate, with back-and-forth transformation between various forms [30]. It should be noted that researchers seldom regard humans as a part of the As cycle but more as the ultimate victims of As toxicity. However, to the whole As cycle on Earth, humans are also an extremely important part of it. Such a viewpoint should be well integrated into current ongoing research projects and continue to be strengthened in future studies.

## 4 Organisms' arsenic-coping strategies

Arsenic is a naturally occurring omnipresent element, and even the first life on Earth was accompanied by exposure to As [89]. The toxicity of As is closely related to the As metabolic ability of organisms. When encountering As stress, a higher metabolic rate can effectively reduce the toxicity of As to organisms. The As compounds have acute and chronic effects on individuals, groups, and communities, with concentrations ranging from a few micrograms per liter to milligrams, depending on species, exposure time, and measurement endpoint [90]. These effects include lethally inhibition of growth, photosynthesis, and reproduction, and impairment in behavioral performance, as well as As-induced degradation of environmental attributes that constrain species abundance and diversity [30]. In this section, we discuss organisms' coping strategies towards As contamination from the viewpoints of toxicity and detoxification.

### 4.1 Microbial coping strategies

Microbes play a very important role in As cycle [91]. The first batch of microbes evolved a detoxification mechanism under the toxicity of high concentrations of As, some of which were able to tolerate or resist As, while others were able to obtain energy from As respiration [92]. However, most microbes exhibit symptoms of poisoning under As stress that can interfere with cell metabolism and damage DNA. The ingestion of As by microbes is the primary cause of As poisoning. AQPs are the primary channels for organisms to absorb various substances, including As^III^. In eukaryotes, AQPs, especially Fps1p, function as predominant transporters facilitating the bidirectional movement of As^III^ across cellular membranes [93]. Inhibition of Fps1p expression can enhance cell tolerance to As^III^ exposure, while its activation renders cells more susceptible to this toxicant. Due to the structural similarity between As^V^ and phosphate, As^V^ often enters cells through phosphate transporters and competes with phosphate for internal transport. The phosphate concentration indirectly affects the accumulation and biotransformation of As in cells. In yeast cells, As^III^ can induce toxicity to the cell from three aspects: 1) increasing the level of reactive oxygen species (ROS), 2) destroying the spatial structure of proteins, and 3) inhibiting DNA repair [94]. The ultimate manifestation at the cellular level is to inhibit the growth and vitality of yeast cells, leading to their apoptosis. Although As is used as an electron donor or acceptor by some bacteria, it does not play any metabolic or nutritional role in the cytoplasm. Therefore, microorganism cells do not need intracellular As or As^III^, nor do they develop any special As absorption system [91].

The conversion of iAs to MA species has been considered the primary detoxification pathway of As [95]. However, the production of trivalent methylated metabolites, which requires the same methyl donor as biological enzymes such as DNA MeTases, may activate As as a toxin and a carcinogen, making it a controversial topic that needs further research [87]. Although As^V^ can inhibit oxidative phosphorylation and block the primary energy production system, As^III^ is more toxic since it binds to thiols and impairs the functions of many proteins [96]. arsM enzymes are critical for the microbial conversion of iAs to volatile MA and hydrogen arsenide [97]. The heterologous expression of *arsM* from *Rhodopseudomonas palustris* has been shown to confer As^III^ resistance to an As-sensitive strain of *Escherichia coli*. *arsM* catalyzes the formation of several methylated intermediates from As^III^, with trimethylarsine as the end product [97]. The resistance of bacteria to As is determined by the plasmid, which also encodes resistance to other heavy metal ions and antibiotics. It involves a plasmid-encoded oxyanion transport system that is encoded by a single cluster of genes, designated the *ars* operon [98]. The specific gene in the operon that is responsible for As reduction in bacteria is *arsC* [99]. Studies have shown that the As-reducing genes (*arsABCDRT* and *acr3*) account for the highest proportion of common genes in high As metabolism microorganisms, and the proportion of As-reducing genes in all As metabolism genes is more than 70%, accounting for the vast majority [96]. However, the reduction of As in the environment by microbes can also harm their own energy metabolism, DNA replication and repair, and membrane transport, which are the substitutable harmful effects of As^V^ due to its structural similarity with phosphate [96].

### 4.2 Plant coping strategies

Plants, especially crops, are a crucial component of human society, and the toxic effects of As on plants are significant issues of concern. The phytotoxicity of As ultimately manifests as delayed plant growth and development, as well as reduced crop productivity, which may be attributed to the disruption of normal metabolic functions [100]. The toxicity of As to plants can lead to three types of responses in plants: 1) morphological changes, including leaf number reduction and defoliation, chlorosis, and necrosis, 2) physiological responses such as reduced bud and root growth, limited stomatal conductance and nutrient absorption, chlorophyll degradation, and limited biomass and yield production, and 3) production of excessive ROS in biochemical reactions, leading to damage of carbohydrates, proteins, and DNA [101].

The impact of As on plant photosynthesis systems is manifested at both the photochemical and biochemical stages. The accumulation of As within plants results in damage to the chloroplast membrane, which leads to disruption of the photosynthetic process and a reduction in carbon dioxide fixation rates by interfering with PS-II function [102]. Due to its structural similarity to Pi, As^V^ inhibits the formation of ADP and rapid self-hydrolysis, disrupting the connection between oxidative phosphorylation and photophosphorylation. This leads to impaired ATP production and compromises cellular metabolism [103]. The cell membrane is the most vulnerable component of plants when exposed to As stress, and the extent of membrane damage can serve as an indicator of stress tolerance [104]. It has been reported that an increase in As accumulation leads to a rise in malondialdehyde (MDA, a byproduct of membrane lipid peroxidation) content and results in electrolyte leakage [105].

Plants have several detoxification mechanisms for As, including metal-binding proteins and specific transporters or compartments[106]. Specifically, the As that enters the plant will chelate with phytochelatins (PCs) and metallothioneins (MTs). This is the first step of As detoxification in the plant [93]. Glutathione and the sulfhydryl group of PCs will combine with As^III^ as much as possible and isolate them into vacuoles to reduce the toxicity of As to other tissues in cells. ABC transporter found in Arabidopsis can also detoxify As [107]. Previous research suggested that plants cannot methylate As to reduce the toxicity of As^V^ and As^III^; instead, they can reduce As^V^ to As^III^ and store it in vacuoles. As a result, plants have evolved a type of hyperaccumulation of As, creating a different approach to As remediation from microbes [108]. For example, tomato is the most likely crop species to act as an As enrichment plant. Despite its high As absorption capability, the accumulation of As and other heavy metals in its fruit is minimal. Although it also shows that high concentrations of As may inhibit the root length and fruit size of tomatoes, it also indicates that the tomato root system has a self-protection mechanism [109].

Overall, As^V^ enters prokaryotes through phosphate channels and is reduced to trivalent As before excretion from the body. The elimination process of As in eukaryotes is very similar, but they can also deposit As into vacuoles when present. This is a natural detoxification mechanism developed by organisms; this mechanism appears to be consistent across different species, stemming from the evolutionary process. In the future, it may be possible to control As intake by controlling the expression of specific gene products to reduce its toxicity to organisms.

### 4.3 Possible coping strategies in animals and humans

Different forms of As can be released from various sources into the environment, and their accumulation can pose potential threats to human health. Exposure to high levels of As has been associated with many health issues, such as cancer, skin lesions, and cardiovascular disease. The target and tolerance mechanisms of As toxicity to mammals are similar to those of eukaryotes [110], producing ROS and damaging proteins and DNA as the main toxic mechanisms.

Arsenic targets mitochondria. Specifically, As^III^ reduces the mitochondrial membrane potential of exposed cells, leading to a reduction in ATP synthesis. By contrast, As^V^ directly competes with Pi, which also results in the inhibition of ATP synthesis, thereby inhibiting various activities of cells [111]. The influence of As on human health is very significant, with both chronic and acute toxicity. Symptoms of As poisoning include skin damage, liver fibrosis, and cancer. These symptoms are widely reported in areas with high As contents, such as the Taiwan region, where “blackfoot disease” is reported [112]. Noncirrhotic portal fibrosis in children has been reported to be associated with high As exposures in India [113]. It is estimated that the increase of nearly 9,000–11,900 cases of bladder cancer, 12,000–121,000 cases of lung cancer, and 11,000–110,000 cases of skin cancer worldwide is due to As exposure [114]. Through investigation of these cases, it has been discovered that they possess common characteristics. When GLUT1 and GLUT4 (two glucose transporters) are heterologously expressed in human cells, they catalyze the uptake of As^III^ and MMA^III^ [115]. GLUT1 is the main glucose transporter in red blood cells and epithelial cells that form the blood-brain barrier, so GLUT1 may be the pathway for iAs and methylene As to enter these tissues, leading to cardiovascular problems or neurotoxicity. Similarly, GLUT4 may contribute to As transportation into cardiac myocytes, thus acting as a promoter of As-related cardiovascular diseases [116].

However, even though the toxicity and detoxification mechanisms expressed by microbes and plants under As stress have been extensively studied, the detoxification mechanism of animals and humans under As stress has not been fully understood. Due to its relatively low toxicity of oAs, the methylation of iAs into oAs after its intake in many animals has been considered a primary biological method for As immobilization and detoxification [95]. However, this viewpoint remains controversial [87,117]. Since As poisoning is mainly caused by oxidative stress induced by iAs, which subsequently leads to DNA damage and cell death, existing drugs designed to alleviate As toxicity mainly target these characteristics. Significant examples of such drugs include curcumin, selenium, N-acetylcysteine, melatonin, propolis, etc [118]. The toxicity and species diversity of ecosystems are different between natural and artificial As sources. In high-As environments, biological growth is relatively low, while in natural high-As environments, this impact is significantly reduced when phosphorus concentration is high [76]. Currently, the detoxification mechanism of organisms facing As stress is still dominated by specific species, and no theory that covers most species has been proposed [117]. As a result, using biological means to deal with As problems often involves a lot of repetitive work. Therefore, it is imperative for researchers to further explore the impact of long-term As stress in animals, and work collaboratively to propose a universal theory that could tackle As toxicity and speed up the progress in these areas.

Humans have also been exploring external detoxification ways to deal with As toxicity. Many studies also used chemicals as dietary supplementation to counteract As toxicity [119]. Selenium was increasingly recognized as a versatile anticarcinogenic agent in the 1990s, especially As [120], since it was regarded as nature's antidote to heavy metal toxicity. However, *in vivo* and *in vitro* tests showed that exposure to selenium could also induce damage and chromosomal aberrations [121]. However, it has been shown that short-term dietary intervention with selenium can reduce its side effects without compromising the reduction of As toxicity, which may help protect against the widespread health problems induced by As-contaminated drinking water [122]. Other possible antidotes, such as vitamin E [123], garlic extract [124], and fruit extract [125], could also be used as dietary supplementation to counteract As toxicity.

## 5 Current solutions to mitigate arsenic contamination

### 5.1 Conventional techniques

Apart from considering the possibility of relying on methylation and other intrinsic detoxification pathways in living organisms, the scientific community is also interested in different As sorption, particularly adsorption, and removal methods as possible solutions to As contamination in the environment. Minerals, especially clay minerals and iron oxides, are found to be effective constituents for As adsorption because of their high affinity for As [16]. Generally, clay minerals exhibit less As^III^ adsorption than As^V^ adsorption [126]. Similarly, activated carbon, ferrihydrite [127], iron hydroxides [128], and manganese oxides [129] are also effective adsorbents for As. Among them, activated carbons and iron oxides (e.g., hematite, magnetite, and goethite) are frequently used to remove As from groundwater [130]. Some activated carbons impregnated with metallic, such as those impregnated with iron, are effective in As removal as well [130]. For iron oxide minerals, As^V^ is strongly sorbed to iron^III^ oxides (α-Fe_2_O_3_) and oxide hydroxides (α-γ-FeOOH) but poorly to crystalline ferrihydrite (hydrous ferric oxide). It should be noted that zerovalent iron is also frequently used as an effective sorbent for As in potable water supplies and other groundwater systems [131]. Together, As adsorption on different iron oxides (hematite, magnetite, and goethite) is a function of different parameters, including pH, types of sorbents, and time [132]. The remediation methods for As-contaminated sites are similar to those for most heavy metal-contaminated soils, including isolation, fixation, supervision, physical separation, and extraction [40].

### 5.2 Emerging arsenic-mitigation techniques

Recent advances in chemistry and material sciences have significantly advanced As removal techniques in aqueous solutions, especially sorption (including absorption and adsorption), due to its ease of operation and the availability of a wide range of adsorbents [133]. In the past decade, new generations of adsorbents have been developed, especially nanoparticles. Generally, nano-adsorbents can remove 5–10 times more As than their micron-size counterparts. They are regarded as the major step towards achieving the nanoscale effects and properties that have been utilized by nature for millions of years [134]. Nanomaterials for As immobilization and removal were first introduced in the late 2000s when traditional adsorbents were synthesized at the nanoscale with better removal capacities, such as nano zerovalent iron [135], nano-iron-oxide-coated quartz [136], and bimetal oxide magnetic nanomaterials [137]. Nevertheless, more studies are required to make the technology applicable under field conditions, e.g., inexpensive and highly selective nano-adsorbents, which have been the focus in recent years [138].

Meanwhile, biochar has emerged as a promising tool for addressing As contamination in aqueous solutions. Biochar is a solid material produced through the thermochemical conversion of biomass under oxygen-limited conditions [139]. It can be a good substitute for activated carbons since they are plentiful, inexpensive, and locally available [63]. Recent studies have shown that different types of feedstock, including agricultural, solid and sludge wastes, and industrial by-products, are promising sources to produce biochar, offering additional waste management value [140]. Various types of biochar from different origins have been proposed with increased removal efficiency in the last few years, while the possibilities of using new types of feedstocks have been reported more recently [141]. Moreover, increasing research interests have been given to the modified biochar, which exhibited high sorption efficiency for As [142], such as iron-impregnated biochar [143], and magnetic biochar where γ-Fe_2_O_3_ or γ-FeOOH particles on the biochar served as sorption sites for As [144].

There are several bacterial remediation methods to combat As pollution, including biosorption, bioaccumulation, biotransformation, bioleaching, and biomineralization. Biosorption is a passive process [145], whereas bioaccumulation is driven by energy [146]. Scientists are exploring the combination of both methods to overcome their shortcomings. Genetic engineering may also be necessary to enhance the metal tolerances of microbial strains [147]. Additionally, the biotransformation of metal-citrate complexes to an alkaline pH has been shown to achieve the complete removal of metals through precipitation and co-precipitation [148]. Bioleaching heavy metals from sediment using autotrophic bacteria combined with heterotrophic bacteria can effectively improve bioleaching efficiency and reduce toxicity [148]. Some bacteria produce urease, which can hydrolyze urea, increasing soil pH and producing carbonate, leading to the mineralization of soluble heavy metal ions in soil water [149].

Moreover, various biological combinations of bacteria, fungi, and algae have been proven to exhibit high efficiency in removing heavy metals from complexly polluted environments while maintaining stability [150]. The bioaccumulation of As by plants may also provide a means for As removal from contaminated soils and waters [151], especially in some places with high iAs concentrations. Ma et al. first reported that Chinese Brake fern (*Pteris vittata*) can hyperaccumulate As from As-contaminated land and proposed its potential to remove As from contaminated soils [152]. Several plants have also been identified as hyperaccumulators of As. For example, *Pityrogramma calomelanos* is potentially a good phytoremediator of As-contaminated soils [153]. Compared with non-hyperaccumulators, hyperaccumulating species possess a higher antioxidant capacity with a relatively low concentration of ROS [154].

Overall, the applicable treatment methods vary between different environments (Table 1) [[135], [136], [137],[141], [142], [143], [144], [145],^152^,^153^,[155], [156], [157], [158], [159], [160], [161], [162], [163], [164], [165], [166], [167], [168]], so it is necessary to formulate plans for specific use scenarios. So far, time-proven bioremediation technologies, such as phytoremediation, can be most widely used. They can promote each other with microbes to better treat groundwater and soil [169]. One microbial agent particularly adept at detoxifying AsO_4_^3−^/H_3_AsO_4_ is *Chrysiogenes arsenatis*, which uses nitrate and nitrite as electron acceptors [170]. These combined methods complement each other and can represent the future of As removal in the environment.

**Table 1:** Emerging As mitigation techniques and their advantages and disadvantages.

Category	Technique	Target	Advantage	Disadvantage	Reference
Nano materials	Conventional nanomaterials	Water	• Rapid adsorption	• Adsorption affected by phosphates and silicates • Not applicable to drinking water • Low adsorption capacity	[135,155]
	Biocomposite nanomaterials	Water	• Robust • Less toxic • Can be regenerated and reused	• Complex preparation process • High energy consumption	[136,156,157]
	Bimetal oxide magnetic nanomaterials	Water	• A high adsorption capacity for both As^III^ and As^V^ • Relatively convenient elution and high regeneration efficiency	• Adsorption affected by phosphates and silicates	[137,158]
Biochar	Conventional biochar	Water	• Wide and inexpensive sources of materials • Rapid adsorption	• High pH requirements • Low adsorption capacity • Poor stability	[141,159]
	Modified biochar	Water	• Low regeneration cost • Large adjustable space • High adsorption capacity	• Consume a considerable amount of energy • Low cost	[143,144,159]
Biosorption	Bacteria	Soil Water	• Easy to operate • Low cost • Possible genetic customization	• Low removal efficiency or economic efficiency. • High requirements for the working environment	[145,160,161]
	Fungi	Soil	• High adsorption efficiency • Large adsorption capacity • Simultaneously adsorbing other heavy metals	• Adsorption effect affected by the presence of other heavy metals	[[162], [163], [164]]
	Algae	Water	• High adsorption efficiency at low concentrations of heavy metals • Not affected by temperature	• High pH requirements	[[165], [166], [167]]
	Plants	Soil	• A high total adsorption capacity • Convenient for subsequent processing	• The inability to transfer • High cultivation time costs	[152,153,168]

## 6 Pathways to arsenic sustainability: A robust framework

In the previous contents, we discussed the issue of As pollution that arises from both natural and human activities and explored the progress and gaps related to the three key questions mentioned in the introduction. Specifically, we summarized the research gaps identified and suggested that As contamination is of great concern in many Asian and African countries that are densely populated and heavily dependent on local resources. This indicates that we need to consider As contamination in a broader context. In this section, we summarize the research gaps identified in this work and propose a framework based on the One Health concept, shedding light on future As research topics of an urgent need for sustainability.

### 6.1 Opportunities for improvement

The As cycle is a biogeochemical process similar to the carbon cycle, and thus both natural and anthropogenic activities contribute to As pollution, mostly iAs. Anthropogenic sources tend to produce higher concentrations in soil than natural sources do in water. However, high As concentrations in surface water from human sources have been underrepresented in research. Previous As poisoning events suggest that high As in groundwater is more harmful, especially in areas where there is limited water availability and a lack of treatment facilities that makes water purification challenging.

Many studies have investigated the role of organisms in the As cycle, with a focus iAs due to its higher toxicity compared to oAs. Plants have evolved various mechanisms to reduce iAs toxicity, such as chelation and compartmentalization into vacuoles. Recent studies suggest that plants under heavy metal stress activate antioxidant enzymes and proteins that generate endogenous hydrogen sulfide (H_2_S) gas, which can chelate heavy metals. Quantitative testing of H_2_S gas in plants may help assess the degree of heavy metal pollution in an area. Although animals and humans typically acquire As through food and drinking water, their role in the As cycle is not well understood. More research is needed to elucidate this role.

Organisms have evolved diverse mechanisms to cope with As exposure and reduce its impact. With the movement of As from soil to microorganisms, animals, and plants, it becomes enriched in the environment. The absorption and metabolism of As by organisms not only reduce As toxicity but also weaken its overall environmental toxicity. Despite this, current research often neglects the role of mammals in reducing As toxicity, focusing instead on their exposure to it. To effectively address As pollution, it is crucial to consider the role of all organisms in the As cycle. While plants and microbes are promising for removing As due to their enrichment and metabolism, animals occupy a significant role in the ecosystem and cannot be overlooked. Therefore, research on the role of animals in the As cycle needs to be strengthened to identify more effective methods for mitigating As toxicity.

Last but not least, while traditional As removal methods such as oxidation and membrane technology are commonly used, they are often inaccessible due to high costs or difficulties in subsequent treatments. Scientific breakthroughs are now addressing these shortcomings. For example, adsorption is becoming a more prominent method for treating high-As water due to advances in chemistry and materials science. Biological methods, such as phytoremediation and microbial remediation, are other viable options. However, these techniques also have limitations, as no single plant can adapt to all environments. The future of As removal technologies may lie in combining biological methods with adsorption treatment for lower concentrations. This combination could leverage the advantages of both approaches to provide a more cost-effective and sustainable solution to As pollution.

### 6.2 A One Health framework for arsenic sustainability

**Fig. 5:**
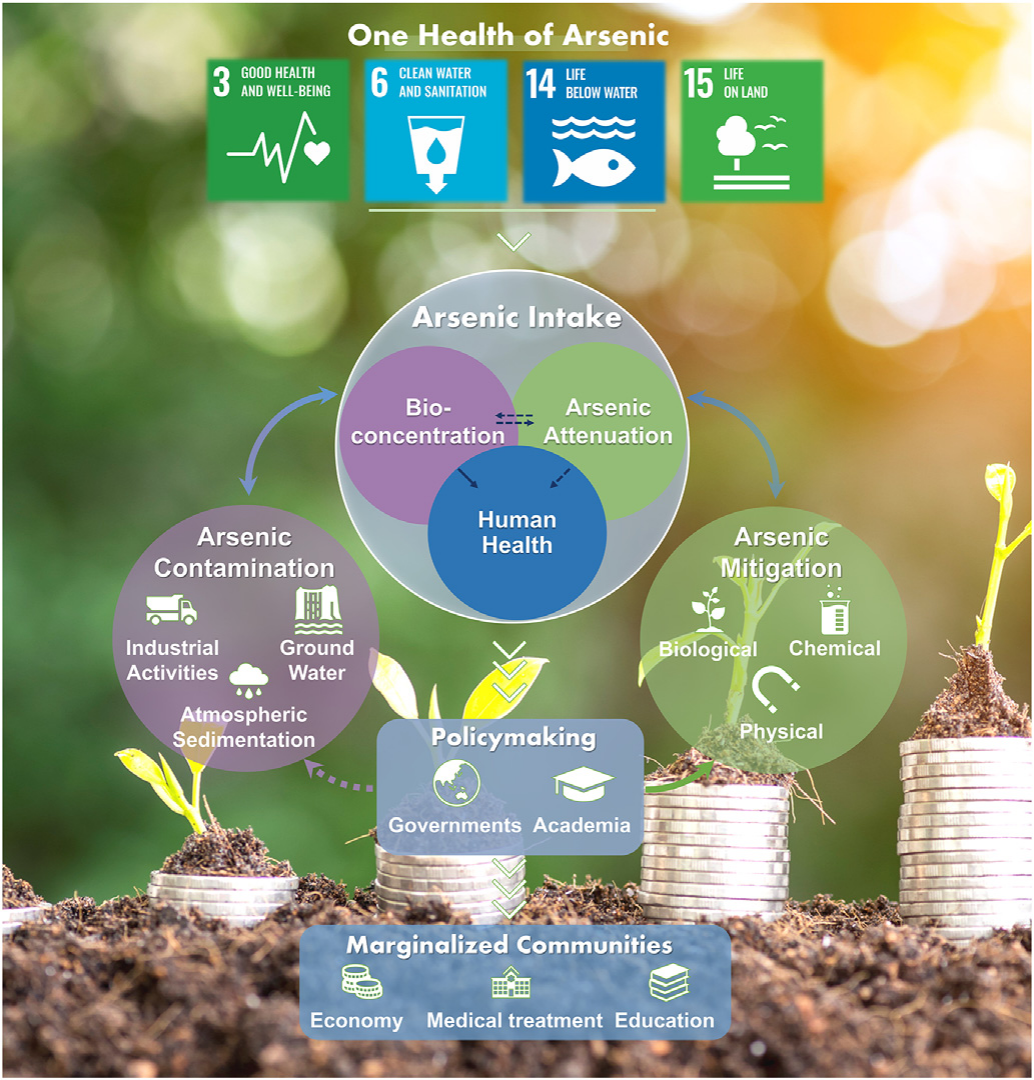
A proposed framework with the One Health concept to achieve As sustainability. The control of As pollution is not achievable by a single entity. In the context of global As pollution, international organizations and institutions such as WHO, UN, and FAO must work together to form a unified agenda on As mitigation strategies to achieve a sustainable As future. Corresponding policies should then be formulated through governments and academic communities to provide feedback and adjust the guidance of global organizations accordingly. Such joint efforts should be the focal point of further As research, facilitating the achievement of SDGs. Arrows with dashed lines indicate where research gaps are, and special attention should be given. Arrows with solid lines suggest the interactions between the three key questions identified in this study. Arrowheads indicate the actions the joint efforts and actions that are needed to ensure the implementation of the proposed framework and subsequent As sustainability. Credit for field image: Microsoft 365.

Arsenic contamination is a global problem that requires a comprehensive approach to mitigate its impact on the biota, especially on humans. To cope with the problems identified in the previous section, a One Health framework that integrates all available resources and strategies is promising to achieve this goal (Fig. 5). Similar to the efforts made to contain global warming, collective actions and multidisciplinary approaches are essential to introduce laws, regulations, and treaties that reduce As emissions and mitigate further damage.

While the focus on mitigating the impact of As contamination on environmental and human health is vital, it is imperative to underscore the One Health concept's capacity to address health disparities and support marginalized communities. The profound effects of As contamination is not uniformly distributed. They often disproportionately affect vulnerable populations such as low-income communities, indigenous groups, and regions with limited access to healthcare, especially in LMICs. These groups usually represent the marginalized communities that are most affected by As exposure. Using China as an example, marginalized communities are emerging due to the ongoing progress of urbanization, resulting in a continual decline in rural populations. These communities primarily consist of elderly residents, facing challenges related to inadequate sanitation conditions and a lack of preventative measures for As exposure. One Health's emphasis on interconnectedness extends beyond biological and environmental factors. It encapsulates the social determinants of health, including economic status, education, and access to healthcare. Applying this lens to As contamination reveals the intricate interplay between environmental degradation, compromised health, and social injustices.

Efforts to mitigate As impacts, therefore, should incorporate a two-pronged approach that considers both environmental rehabilitation and community empowerment. Such an approach calls for collaborative engagement with affected communities, recognizing their insights, needs, and aspirations. By involving these communities in decision-making processes and tailoring mitigation strategies to their unique circumstances, the impact of As contamination can be more effectively reduced. Academia and policymakers should work together to create holistic solutions that not only alleviate environmental damage but also empower communities with better health resources and opportunities. This could involve educating communities about safe water sources, promoting hygiene practices, and disseminating information about alternative products that are safer than As-containing ones.

Specifically, bold and proactive engagements and actions should be taken. For example, in developed regions, a ban on the use of pesticides and artificial products containing As should be considered. As a matter of fact, the utilization of As in French vineyards was prohibited as early as 2003 [171]. Similarly, the United States has implemented measures to control and regulate As levels in public water supply systems [172]. Subsequently, additional pretreatment measures for drinking water should be implemented in regions where groundwater serves as the primary source of drinking water. By contrast, for economically disadvantaged regions like most African countries and LMICs, governments, together with non-governmental organizations, industries, and academics, should step in to provide low-cost As removal agents in substantial quantities, thereby ensuring the basic clean domestic water and food supply for local residents. Such actions will significantly diminish the intake of As and mitigate its harmful effects on individuals. To further elucidate the framework, consider the case of China:

Step 1 The Chinese central government plays a pivotal role in establishing the overarching direction, including but not limited to rules and regulations. This is the key to achieving environmental remediation and protection strategies, including sustainable As mitigation.
Step 2 Local authorities at the provincial, municipal, and county levels delineate emission baselines for enterprises based on their economic status and the prevailing environmental conditions. Penalties are imposed for emissions surpassing the stipulated limits.
Step 3 The academic and industry sectors are proactively encouraged by governments at various levels to delve into As removal and mitigation techniques [[173], [174], [175]]. This support is manifested through initiatives such as the establishment of funds aimed at advancing fundamental sciences like materials science and chemistry.
Step 4 Safety networks and feedback loops are established to safeguard the community's economy, promoting economic self-reliance and sustainability with a specific focus on pollution mitigation and similar endeavors. The governments may commission scholars to conduct a comprehensive assessment of As content in groundwater and soil at the national level [176].
Step 5 In cases where economic feasibility allows, residents in high-As areas identified and confirmed by such assessments could be relocated. Such a multifaceted approach underscores China's commitment to addressing As-related challenges comprehensively and sustainably.

In summary, our proposed framework integrates environmental, animal, and human health, considering social determinants and fostering collaboration between diverse disciplines. By prioritizing the inclusion of marginalized communities, acknowledging health disparities, and promoting education, a comprehensive One Health approach has the potential to not only mitigate the impact of As contamination but also promote equitable health outcomes and sustainable futures for all beings. More importantly, these actions will be of great importance to achieving the United Nations SDGs, especially SDGs 3, 4, 14, and 15 (Fig. 5).

## CRediT authorship contribution statement

C.L. and J.J conceived the idea. C.L. and Y.H. led the writing, with authors contributing to the final version of this manuscript.

## Declaration of competing interests

The authors declare no conflict of interest.
