# Supplementary material for: Leveraging the One Health concept for arsenic sustainability
Source: Eco Environ Health. 2024 Mar 7;3(3):392–405. doi: 10.1016/j.eehl.2024.02.006 (PMC11401129; doi:10.1016/j.eehl.2024.02.006)
Supplement: Multimedia component 1 [file mmc1.docx]

*Supplemental Information*

**Leveraging the One Health concept for arsenic sustainability**

Yujie Huang^1^, Raymond W.M. Kwong^2^, Qi Miu^1^, Dapeng Zhang^1^, Yuchuan Fan^3^, Min Zhou^4^, Jianbo Jia^1*^, Bing Yan^1^, Chengjun Li^1,5*^

^1^ Institute of Environmental Research at Greater Bay Area, Guangzhou University, Guangzhou, 510006, China

^2^ Department of Biology, York University, Toronto, ON, M3J 1P3, Canada

^3^ Department of Soil, Water, and Ecosystem Sciences, University of Florida-IFAS, Gainesville, FL, 32603, USA

^4^ Centre for Catalysis and Clean Energy, Gold Coast Campus, Griffith University, QLD 4222, Australia

^5^ School of Agriculture and Biological Sciences, Qiannan Normal University for Nationalities, Duyun 558000, China

^*^Author for correspondence; Email: [jiajianbo03@gmail.com](mailto:jiajianbo03@gmail.com); [cli@gzhu.edu.cn](mailto:cli@gzhu.edu.cn)

This Supplemental Information includes the following contents:

1. Three Supplemental Methods (Database search, Web-enhanced search, and, Data plotting),
2. One Supplemental Figure (Figure S1),
3. Four Supplemental Tables (Tables S1-S4), and
4. 464 Supplemental References (cited in Tables S1 and S3).

# Supplementary Methods

## **Database search**

Relevant papers were retrieved from the Web of Science (WoS) Core Collection with the searching phrase restricted as “arsenic” and category restricted as “Topic”. The access to WOS core collection was granted by Nanjing University. In the core collection, we selected the “All years” timespan during the time of searching (December 2022) to retrieve as many peer-reviewed articles as possible. We then further restricted nine major document types included in these records (i.e., Article, Proceedings Paper, Review Article, Meeting Abstract, Editorial Material, Letter, News Item, Early Access, and Book Chapters). As of the time of writing, there were a total number of 61,883 records on arsenic research in the database. in which article was the dominant (50,016), contributing more than 80% of the total publications. We systematically reviewed relevant papers according to the structure of this work and referred to and cited relevant studies in this work, if necessary.

## **Web-enhanced search**

Within the searching record of WoS, we further looked into the geological distribution (countries/regions filter in WoS) to collect the concentration of As in the water and soil on a global scale. Specifically, we refined the countries/regions filter to one county at a time to collect concentration data possibly available in published literature. For regions with tens of hundreds of studies, we also used Google Search to speed up the process by searching country name plus “water” or “soil” with “arsenic concentration” as the keywords. The resulting records were matched with those in the records in WoS. This helped us rapidly identify candidate studies that were highly likely to have usable data. When we confirmed that a specific study was qualified, we recorded the highest As concentration and its measurement time in different papers in the country. If some papers did not record the measurement time, we used the year before the publication date of their papers as the measurement time for this data.

## **Data plotting**

A total number of 464 qualified publications were manually inspected to collect possible concentration data. Specifically, soil and water As concentration data in qualified publications are collected and presented in **Tables S1-S4**, which were used for plotting **Figure 1** and **Figure 2** in the main text. To reduce the number of plots, the timespan in the map plotting was refined into six different time intervals, i.e., “Before 2000”, “2001-2005”, “2006-2010”, “2011-2015”, “2016-2020”, and “After 2020”. All concentration data in each county, if available, was recorded and then averaged based on the time intervals provided. To reflect the worst-case scenario of groundwater contamination situation, the highest concentration value in a single study was collected. Then, data points within the same time intervals of the same region were averaged to represent the value within the same region in that specific interval. For instance, in China, there were three individual studies about the groundwater arsenic (As) concentrations recorded for the Years 2006, 2007, and 2010, respectively, within the 2006 to 2010 period. Therefore, we collected the highest value for each year and recorded them in Table S1. By summing these concentrations and dividing them by the corresponding three-year periods, the average of the highest arsenic concentration for these five-year intervals was calculated.

# Supplementary Figures


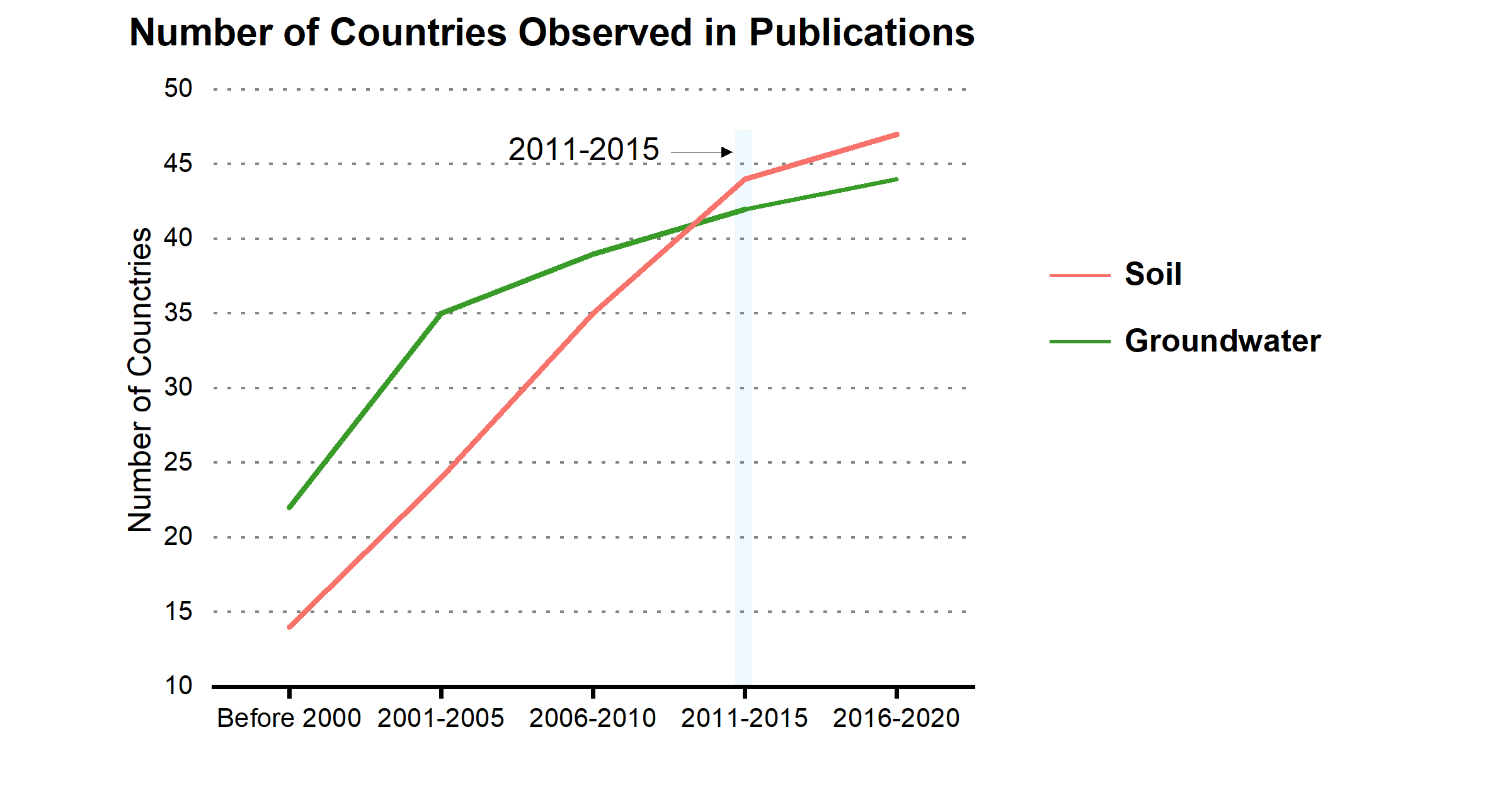


**Figure S1.** The total number of countries observed during the past 40 years that reported either soil or groundwater contamination by arsenic. The “After 2020” period is excluded from the plot. Data were collected from qualified publications as described in **Supplementary Methods**.

# Supplementary Tables

**Table S1**. Global arsenic concentrations in groundwater. Data are collected from qualified articles as described in **Supplementary Methods**. Average concentration (ppm) is determined by averaging the concentrations in the predefined time intervals in **Supplementary Methods**.

| **Country** | **Sample year** | **Highest Concentration (ppm)** | **Average Concentration (ppm)** | **Reference** |
| --- | --- | --- | --- | --- |
| **Korea** | 2000 | 0.167 | 0.167 | [1] |
| **Korea** | 2003 | 0.187 | 0.098 | [2] |
| **Korea** | 2005 | 0.009 |  | [3] |
| **Korea** | 2008 | 0.143 | 0.143 | [4] |
| **Korea** | 2011 | 0.178 | 0.1132 | [4] |
| **Korea** | 2015 | 0.0484 |  | [5] |
| **Korea** | 2017 | 5.594 | 5.594 | [6] |
| **India** | 1995 | 3.7 | 3.7 | [7] |
| **India** | 2007 | 1.059 | 1.162667 | [4] |
| **India** | 2008 | 1.891 |  | [8] |
| **India** | 2009 | 0.538 |  | [4] |
| **India** | 2012 | 0.143 | 0.788667 | [9] |
| **India** | 2013 | 1.362 |  | [10] |
| **India** | 2014 | 0.861 |  | [11] |
| **India** | 2017 | 4.73 | 4.73 | [12] |
| **Finland** | 1999 | 1.04 | 1.04 | [8] |
| **Finland** | 2014 | 2.23 | 2.23 | [13] |
| **Nepal** | 2004 | 0.74 | 1.68 | [4] |
| **Nepal** | 2005 | 2.62 |  | [14] |
| **Nepal** | 2006 | 0.456 | 0.735 | [15] |
| **Nepal** | 2007 | 1.014 |  | [16] |
| **Nepal** | 2014 | 1.8 | 2.2145 | [17] |
| **Nepal** | 2015 | 2.629 |  | [8] |
| **Nepal** | 2017 | 4.73 | 4.73 | [12] |
| **Turkey** | 2002 | 7.754 | 8.527 | [18] |
| **Turkey** | 2004 | 9.3 |  | [19] |
| **Turkey** | 2008 | 0.17 | 0.17 | [20] |
| **Turkey** | 2011 | 0.0744 | 0.2099 | [21] |
| **Turkey** | 2012 | 0.3454 |  | [22] |
| **Cambodia** | 2007 | 2.5 | 1.794333 | [23] |
| **Cambodia** | 2008 | 1.543 |  | [24] |
| **Cambodia** | 2010 | 1.34 |  | [25] |
| **Cambodia** | 2017 | 1.31 | 1.46 | [26] |
| **Cambodia** | 2018 | 1.61 |  | [27] |
| **Cambodia** | 2021 | 0.5 | 0.5 | [28] |
| **China** | 1996 | 1.354 | 1.354 | [29] |
| **China** | 2002 | 1.48 | 1.48 | [4] |
| **China** | 2006 | 12 | 6.286 | [4] |
| **China** | 2007 | 0.572 |  | [4] |
| **China** | 2010 | 4.44 |  | [25] |
| **China** | 2011 | 2.33 | 2.36 | [30] |
| **China** | 2012 | 0.51 |  | [31] |
| **China** | 2013 | 2.16 |  | [32] |
| **China** | 2016 | 2 | 2.335 | [27] |
| **China** | 2017 | 4.73 |  | [12] |
| **China** | 2018 | 0.275 |  | [33] |
| **China** | 2021 | 0.356 | 0.356 | [34] |
| **USA** | 1987 | 0.15 | 9.075 | [35] |
| **USA** | 1988 | 18 |  | [36] |
| **USA** | 1997 | 2.62 | 7.31 | [4] |
| **USA** | 1999 | 12 |  | [4] |
| **USA** | 2002 | 0.171 | 0.104 | [4] |
| **USA** | 2005 | 0.037 |  | [37] |
| **USA** | 2006 | 1.2 | 1.3225 | [27] |
| **USA** | 2007 | 0.09 |  | [37] |
| **USA** | 2008 | 3 |  | [37] |
| **USA** | 2010 | 1 |  | [37] |
| **USA** | 2013 | 3 | 13.65 | [37] |
| **USA** | 2014 | 24.3 |  | [27] |
| **USA** | 2017 | 7.02 | 7.02 | [38] |
| **Canada** | 1983 | 0.086 | 0.086 | [4] |
| **Canada** | 1999 | 0.28 | 0.28 | [39] |
| **Canada** | 2013 | 0.3606 | 0.3435 | [40] |
| **Canada** | 2015 | 0.3264 |  | [41] |
| **Canada** | 2016 | 100 | 100 | [27] |
| **Colombia** | 2012 | 0.225 | 0.1385 | [42] |
| **Colombia** | 2013 | 0.052 |  | [43] |
| **Mexico** | 1996 | 1.097 | 0.9185 | [44] |
| **Mexico** | 1998 | 0.74 |  | [45] |
| **Mexico** | 2002 | 0.277 | 0.2785 | [45] |
| **Mexico** | 2004 | 0.28 |  | [46] |
| **Mexico** | 2008 | 0.3 | 0.462 | [45] |
| **Mexico** | 2010 | 0.624 |  | [25] |
| **Mexico** | 2012 | 0.4 | 1.016 | [47] |
| **Mexico** | 2014 | 2.4 |  | [27] |
| **Mexico** | 2015 | 0.248 |  | [48] |
| **Mexico** | 2022 | 0.35 | 0.35 | [49] |
| **Nicaragua** | 1995 | 0.289 | 0.8045 | [50] |
| **Nicaragua** | 1996 | 1.32 |  | [51] |
| **Nicaragua** | 2000 | 0.069 | 0.069 | [50] |
| **Nicaragua** | 2001 | 0.088 | 0.4653 | [50] |
| **Nicaragua** | 2003 | 0.108 |  | [50] |
| **Nicaragua** | 2004 | 1.2 |  | [50] |
| **Nicaragua** | 2008 | 0.122 | 0.227333 | [50] |
| **Nicaragua** | 2009 | 0.235 |  | [50] |
| **Nicaragua** | 2010 | 0.325 |  | [52] |
| **Nicaragua** | 2011 | 0.057 | 0.5535 | [50] |
| **Nicaragua** | 2014 | 1.05 |  | [50] |
| **Brazil** | 2001 | 0.0066 | 0.7437 | [53] |
| **Brazil** | 2002 | 1.97 |  | [53] |
| **Brazil** | 2004 | 0.2545 |  | [54] |
| **Brazil** | 2011 | 0.03 | 0.09635 | [55] |
| **Brazil** | 2013 | 0.1627 |  | [56] |
| **Brazil** | 2016 | 0.428 | 50.214 | [57] |
| **Brazil** | 2018 | 100 |  | [27] |
| **Burkina Faso** | 2005 | 0.421 | 0.421 | [58] |
| **Burkina Faso** | 2007 | 1.67 | 0.897 | [55] |
| **Burkina Faso** | 2009 | 0.124 |  | [59] |
| **Burkina Faso** | 2016 | 1.63 | 1.63 | [27] |
| **Ethiopia** | 2002 | 0.096 | 0.096 | [60] |
| **Ethiopia** | 2010 | 0.278 | 0.278 | [55] |
| **Ethiopia** | 2012 | 0.19 | 0.1317 | [60] |
| **Ethiopia** | 2013 | 0.0734 |  | [60] |
| **Ghana** | 1992 | 0.18 | 0.1605 | [60] |
| **Ghana** | 1995 | 0.141 |  | [60] |
| **Ghana** | 2006 | 0.004 | 8.406 | [60] |
| **Ghana** | 2007 | 4.5 |  | [27] |
| **Ghana** | 2008 | 28.95 |  | [60] |
| **Ghana** | 2009 | 0.17 |  | [60] |
| **Ghana** | 2011 | 0.0694 | 0.655133 | [60] |
| **Ghana** | 2012 | 1.76 |  | [55] |
| **Ghana** | 2014 | 0.136 |  | [60] |
| **Ghana** | 2019 | 0.434 | 0.434 | [60] |
| **Nigeria** | 2005 | 0.16 | 0.16 | [55] |
| **Nigeria** | 2007 | 0.75 | 0.587 | [27] |
| **Nigeria** | 2009 | 0.424 |  | [61] |
| **Nigeria** | 2011 | 0.4 | 1.73 | [62] |
| **Nigeria** | 2013 | 3.06 |  | [63] |
| **Nigeria** | 2016 | 0.071 | 0.071 | [64] |
| **South Africa** | 2010 | 0.12 | 0.12 | [55] |
| **South Africa** | 2015 | 1.553 | 1.553 | [60] |
| **South Africa** | 2018 | 6.15 | 6.15 | [65] |
| **Argentina** | 1988 | 11.5 | 11.5 | [66] |
| **Argentina** | 1998 | 2.4 | 3.59 | [67] |
| **Argentina** | 1999 | 4.78 |  | [67] |
| **Argentina** | 2004 | 0.5 | 7.7345 | [68] |
| **Argentina** | 2005 | 14.969 |  | [69] |
| **Argentina** | 2008 | 0.25 | 2.292 | [70] |
| **Argentina** | 2009 | 1.326 |  | [71] |
| **Argentina** | 2010 | 5.3 |  | [25] |
| **Argentina** | 2012 | 1.99 | 1.99 | [72] |
| **Argentina** | 2018 | 0.57 | 1.875 | [73] |
| **Argentina** | 2020 | 3.18 |  | [74] |
| **Japan** | 2002 | 0.0422 | 0.0422 | [75] |
| **Japan** | 2015 | 0.0606 | 0.0606 | [76] |
| **Japan** | 2016 | 25.7 | 12.85015 | [27] |
| **Japan** | 2020 | 0.0003 |  | [74] |
| **Pakistan** | 2001 | 2.48 | 2.19 | [77] |
| **Pakistan** | 2002 | 1.9 |  | [78] |
| **Pakistan** | 2007 | 0.106 | 0.106 | [79] |
| **Pakistan** | 2016 | 0.132 | 1.36425 | [80] |
| **Pakistan** | 2017 | 2.09 |  | [81] |
| **Pakistan** | 2018 | 2.58 |  | [27] |
| **Pakistan** | 2019 | 0.655 |  | [33] |
| **Pakistan** | 2021 | 0.1036 | 0.1018 | [78] |
| **Pakistan** | 2022 | 0.1 |  | [82] |
| **UK** | 2002 | 0.103 | 0.103 | [83] |
| **UK** | 2015 | 0.355 | 0.355 | [27] |
| **UK** | 2019 | 13.5 | 13.5 | [84] |
| **Greece** | 2001 | 0.237 | 0.735667 | [85] |
| **Greece** | 2004 | 1.84 |  | [86] |
| **Greece** | 2005 | 0.13 |  | [87] |
| **Greece** | 2006 | 0.07 | 3.220233 | [88] |
| **Greece** | 2008 | 0.0907 |  | [89] |
| **Greece** | 2009 | 9.5 |  | [90] |
| **Greece** | 2011 | 0.0442 | 2.2721 | [91] |
| **Greece** | 2013 | 4.5 |  | [92] |
| **Greece** | 2019 | 0.0547 | 0.0547 | [93] |
| **Germany** | 1998 | 0.025 | 0.2875 | [94] |
| **Germany** | 2000 | 0.55 |  | [27] |
| **Germany** | 2007 | 9 | 9 | [95] |
| **Germany** | 2015 | 0.015 | 0.015 | [96] |
| **Iran** | 2008 | 1.5 | 1.09445 | [97] |
| **Iran** | 2010 | 0.6889 |  | [98] |
| **Iran** | 2012 | 25 | 25 | [99] |
| **Iran** | 2016 | 1.44 | 0.754333 | [1] |
| **Iran** | 2017 | 0.545 |  | [100] |
| **Iran** | 2019 | 0.278 |  | [101] |
| **Bangladesh** | 1999 | 2.19 | 2.19 | [102] |
| **Bangladesh** | 2001 | 0.64 | 2.685 | [4] |
| **Bangladesh** | 2002 | 4.73 |  | [103] |
| **Bangladesh** | 2006 | 1.63 | 0.849 | [60] |
| **Bangladesh** | 2007 | 0.315 |  | [4] |
| **Bangladesh** | 2008 | 0.517 |  | [104] |
| **Bangladesh** | 2009 | 0.934 |  | [4] |
| **Bangladesh** | 2011 | 0.2 | 1.6726 | [105] |
| **Bangladesh** | 2012 | 0.0878 |  | [60] |
| **Bangladesh** | 2014 | 4.73 |  | [27] |
| **Ecuador** | 2009 | 7.853 | 7.853 | [106] |
| **Ecuador** | 2015 | 0.01 | 0.01 | [107] |
| **Togo** | 2007 | 0.3 | 3.11 | [108] |
| **Togo** | 2008 | 5.92 |  | [109] |
| **Iceland** | 2002 | 0.31 | 0.2335 | [110] |
| **Iceland** | 2004 | 0.157 |  | [111] |
| **Sweden** | 2006 | 0.205 | 0.2775 | [112] |
| **Sweden** | 2009 | 0.35 |  | [113] |
| **Sweden** | 2013 | 0.0861 | 0.0861 | [114] |
| **Australia** | 2000 | 30 | 30 | [1] |
| **Australia** | 2002 | 220 | 175.6667 | [115] |
| **Australia** | 2003 | 7 |  | [116] |
| **Australia** | 2005 | 300 |  | [27] |
| **Switzerland** | 2001 | 0.3 | 0.35 | [117] |
| **Switzerland** | 2004 | 0.4 |  | [118] |
| **Italy** | 1996 | 1.479 | 1.479 | [119] |
| **Italy** | 2004 | 6.441 | 3.28475 | [120] |
| **Italy** | 2005 | 0.1285 |  | [121] |
| **Italy** | 2006 | 1.3 | 0.907667 | [27] |
| **Italy** | 2008 | 0.992 |  | [122] |
| **Italy** | 2010 | 0.431 |  | [123] |
| **Italy** | 2011 | 0.14 | 0.2175 | [124] |
| **Italy** | 2012 | 0.295 |  | [125] |
| **Italy** | 2017 | 0.1285 | 0.665 | [126] |
| **Italy** | 2018 | 0.6665 |  | [127] |
| **Italy** | 2019 | 1.2 |  | [125] |
| **Denmark** | 2017 | 0.025 | 0.025 | [128] |
| **Serbia** | 2003 | 0.22 | 0.485 | [129] |
| **Serbia** | 2005 | 0.75 |  | [130] |
| **Serbia** | 2006 | 0.27 | 0.21875 | [129] |
| **Serbia** | 2007 | 0.27 |  | [129] |
| **Serbia** | 2009 | 0.148 |  | [131] |
| **Serbia** | 2010 | 0.187 |  | [132] |
| **Serbia** | 2011 | 0.349 | 0.192333 | [133] |
| **Serbia** | 2012 | 0.0902 |  | [134] |
| **Serbia** | 2013 | 0.1378 |  | [135] |
| **Serbia** | 2016 | 0.01 | 0.18685 | [136] |
| **Serbia** | 2017 | 0.3637 |  | [137] |
| **Chile** | 1999 | 0.4 | 0.4 | [138] |
| **Chile** | 2007 | 0.278 | 1.139 | [139] |
| **Chile** | 2010 | 2 |  | [25] |
| **Chile** | 2017 | 27 | 27 | [27] |
| **Spain** | 2000 | 0.091 | 0.091 | [140] |
| **Spain** | 2001 | 0.26 | 0.1755 | [141] |
| **Spain** | 2005 | 0.04 |  | [142] |
| **Spain** | 2006 | 0.613 | 0.254825 | [27] |
| **Spain** | 2007 | 0.26 |  | [143] |
| **Spain** | 2008 | 0.0583 |  | [144] |
| **Spain** | 2010 | 0.088 |  | [145] |
| **Spain** | 2012 | 0.241 | 0.241 | [146] |
| **France** | 1997 | 3.6 | 3.6 | [147] |
| **France** | 2013 | 0.263 | 0.263 | [27] |
| **France** | 2016 | 0.1 | 0.128405 | [148] |
| **France** | 2019 | 0.15681 |  | [149] |
| **Costa Rica** | 2011 | 0.0141 | 0.0141 | [150] |
| **Costa Rica** | 2017 | 0.063 | 0.063 | [151] |
| **Romania** | 2000 | 0.176 | 0.176 | [152] |
| **Romania** | 2008 | 0.24 | 0.24 | [153] |
| **Vietnam** | 2000 | 3.05 | 3.05 | [154] |
| **Vietnam** | 2003 | 0.198 | 0.198 | [155] |
| **Vietnam** | 2006 | 0.703 | 1.22575 | [156] |
| **Vietnam** | 2007 | 0.34 |  | [4] |
| **Vietnam** | 2008 | 3.05 |  | [27] |
| **Vietnam** | 2009 | 0.81 |  | [157] |
| **Vietnam** | 2011 | 0.5 | 0.715155 | [158] |
| **Vietnam** | 2015 | 0.93031 |  | [159] |
| **Vietnam** | 2016 | 0.884 | 0.847 | [160] |
| **Vietnam** | 2017 | 0.81 |  | [161] |
| **Lao PDR** | 2004 | 0.112 | 0.112 | [162] |
| **Lao PDR** | 2010 | 0.278 | 0.278 | [163] |
| **Lao PDR** | 2014 | 0.005 | 0.01694 | [164] |
| **Lao PDR** | 2015 | 0.02888 |  | [165] |
| **Thailand** | 1995 | 5.114 | 5.114 | [4] |
| **Thailand** | 2005 | 5 | 5 | [166] |
| **Thailand** | 2013 | 0.00364 | 0.00364 | [167] |
| **Thailand** | 2017 | 0.009081 | 0.009081 | [168] |
| **Thailand** | 2021 | 0.48 | 0.307815 | [169] |
| **Thailand** | 2022 | 0.13563 |  | [170] |
| **Guatemala** | 2006 | 0.261 | 0.261 | [171] |
| **Guatemala** | 2013 | 0.0475 | 0.0475 | [172] |
| **Russia** | 2011 | 9.3 | 9.3 | [1] |
| **Russia** | 2017 | 0.035 | 13.7625 | [173] |
| **Russia** | 2020 | 27.49 |  | [174] |
| **New Zealand** | 2002 | 8.5 | 4.855 | [27] |
| **New Zealand** | 2005 | 1.21 |  | [1] |
| **Seychelles** | 1999 | 5 | 5 | [1] |
| **Botswana** | 2003 | 0.112 | 0.112 | [175] |
| **Botswana** | 2007 | 3.28 | 3.24 | [176] |
| **Botswana** | 2010 | 3.2 |  | [1] |
| **Botswana** | 2013 | 0.185 | 0.185 | [60] |
| **Kazakhstan** | 2021 | 0.029 | 0.029 | [41] |
| **Cameroon** | 2008 | 2 | 2 | [27] |
| **Cameroon** | 2017 | 0.2 | 0.1685 | [177] |
| **Cameroon** | 2020 | 0.137 |  | [178] |
| **Burma** | 2013 | 0.63 | 0.54 | [27] |
| **Burma** | 2015 | 0.45 |  | [179] |
| **Mali** | 2015 | 0.132 | 0.132 | [180] |
| **Mali** | 2017 | 0.54 | 0.3395 | [181] |
| **Mali** | 2019 | 0.139 |  | [60] |
| **Malawi** | 2001 | 0.002 | 0.002 | [182] |
| **Malawi** | 2006 | 0.003 | 0.003 | [60] |
| **Malawi** | 2007 | 0.003 |  | [60] |
| **Malawi** | 2018 | 0.015 | 0.069 | [183] |
| **Tanzania** | 2019 | 0.123 |  | [60] |
| **Tanzania** | 2021 | 0.3 | 0.3 | [184] |
| **Qatar** | 2020 | 0.0155 | 0.0155 | [33] |
| **Sri Lanka** | 2017 | 0.034 | 0.034 | [33] |
| **Poland** | 2019 | 0.015 | 0.015 | [185] |
| **Indonesia** | 2007 | 0.065 | 0.065 | [186] |
| **Indonesia** | 2019 | 0.31 | 0.31 | [187] |
| **Ireland** | 2008 | 0.025 | 0.025 | [188] |
| **KSA** | 2012 | 0.029 | 0.029 | [189] |
| **Croatia** | 2004 | 0.619 | 0.619 | [189] |
| **Slovakia** | 2002 | 0.028 | 0.028 | [190] |
| **Slovakia** | 2011 | 0.285 | 0.285 | [191] |
| **Slovakia** | 2016 | 4.9 | 4.9 | [192] |
| **Peru** | 2012 | 0.0931 | 0.0931 | [193] |
| **Zimbabwe** | 2016 | 0.006 | 0.006 | [194] |
| **Jordan** | 2009 | 0.173 | 0.173 | [195] |
| **Yemen** | 2017 | 0.115 | 0.115 | [196] |
| **Netherlands** | 2022 | 0.82 | 0.82 | [197] |
| **Malaysia** | 2019 | 0.072 | 0.072 | [198] |
| **Uruguay** | 2018 | 0.12048 | 0.12048 | [199] |
| **Egypt** | 2018 | 37.55 | 37.55 | [200] |
| **Portugal** | 2002 | 0.18839 | 0.18839 | [201] |
| **Portugal** | 2006 | 9.73 | 4.571333 | [202] |
| **Portugal** | 2007 | 1.434 |  | [203] |
| **Portugal** | 2010 | 2.55 |  | [202] |
| **Portugal** | 2013 | 0.035 | 0.057467 | [202] |
| **Portugal** | 2014 | 0.0707 |  | [204] |
| **Portugal** | 2015 | 0.0667 |  | [204] |
| **Portugal** | 2018 | 0.607 | 0.607 | [203] |
| **Czech Republic** | 2005 | 1.141 | 1.141 | [205] |

**Table S2**. The countries reported As contamination problems with their groundwater during the past four decades. Data are collected from qualified articles as described in **Supplementary Methods**.

| **Country No.** | **Country Name** | **Contaminated Medium** |
| --- | --- | --- |
| **1** | Argentina | Groundwater |
| **2** | Australia | Groundwater |
| **3** | Bangladesh | Groundwater |
| **4** | Botswana | Groundwater |
| **5** | Brazil | Groundwater |
| **6** | Burkina Faso | Groundwater |
| **7** | Burma | Groundwater |
| **8** | Cambodia | Groundwater |
| **9** | Cameroon | Groundwater |
| **10** | Canada | Groundwater |
| **11** | Chile | Groundwater |
| **12** | China | Groundwater |
| **13** | Colombia | Groundwater |
| **14** | Costa Rica | Groundwater |
| **15** | Croatia | Groundwater |
| **16** | Czech Republic | Groundwater |
| **17** | Denmark | Groundwater |
| **18** | Ecuador | Groundwater |
| **19** | Egypt | Groundwater |
| **20** | Ethiopia | Groundwater |
| **21** | Finland | Groundwater |
| **22** | France | Groundwater |
| **23** | Germany | Groundwater |
| **24** | Ghana | Groundwater |
| **25** | Greece | Groundwater |
| **26** | Guatemala | Groundwater |
| **27** | Iceland | Groundwater |
| **28** | India | Groundwater |
| **29** | Indonesia | Groundwater |
| **30** | Iran | Groundwater |
| **31** | Ireland | Groundwater |
| **32** | Italy | Groundwater |
| **33** | Japan | Groundwater |
| **34** | Jordan | Groundwater |
| **35** | Kazakhstan | Groundwater |
| **36** | Korea | Groundwater |
| **37** | KSA | Groundwater |
| **38** | Lao PDR | Groundwater |
| **39** | Malawi | Groundwater |
| **40** | Malaysia | Groundwater |
| **41** | Mali | Groundwater |
| **42** | Mexico | Groundwater |
| **43** | Nepal | Groundwater |
| **44** | Netherlands | Groundwater |
| **45** | New Zealand | Groundwater |
| **46** | Nicaragua | Groundwater |
| **47** | Nigeria | Groundwater |
| **48** | Pakistan | Groundwater |
| **49** | Peru | Groundwater |
| **50** | Poland | Groundwater |
| **51** | Portugal | Groundwater |
| **52** | Qatar | Groundwater |
| **53** | Romania | Groundwater |
| **54** | Russia | Groundwater |
| **55** | Russia | Groundwater |
| **56** | Serbia | Groundwater |
| **57** | Seychelles | Groundwater |
| **58** | Slovakia | Groundwater |
| **59** | South Africa | Groundwater |
| **60** | Spain | Groundwater |
| **61** | Sri Lanka | Groundwater |
| **62** | Sweden | Groundwater |
| **63** | Switzerland | Groundwater |
| **64** | Tanzania | Groundwater |
| **65** | Thailand | Groundwater |
| **66** | Togo | Groundwater |
| **67** | Turkey | Groundwater |
| **68** | United Kingdom | Groundwater |
| **69** | United States of America | Groundwater |
| **70** | Uruguay | Groundwater |
| **71** | Vietnam | Groundwater |
| **72** | Yemen | Groundwater |
| **73** | Zimbabwe | Groundwater |

**Table S3**. Global arsenic concentrations in the soil. Data are collected from qualified articles as described in **Supplementary Methods**. Average concentration (ppm) is determined by averaging the concentrations in the predefined time intervals in **Supplementary Methods**.

| **Country** | **Sample year** | **Highest concentration (ppm)** | **Average concentration (ppm)** | **Reference** |
| --- | --- | --- | --- | --- |
| **Canada** | 1980 | 11 | 11 | [206] |
| **Canada** | 1987 | 280 | 271 | [207] |
| **Canada** | 1990 | 262 |  | [208] |
| **Canada** | 1998 | 70 | 76.63333 | [209] |
| **Canada** | 1999 | 150 |  | [210] |
| **Canada** | 2000 | 9.9 |  | [211] |
| **Canada** | 2001 | 0.51 | 302.2775 | [212] |
| **Canada** | 2002 | 1038 |  | [213] |
| **Canada** | 2003 | 13.6 |  | [39] |
| **Canada** | 2004 | 157 |  | [214] |
| **Canada** | 2006 | 6590 | 6590 | [215] |
| **Canada** | 2019 | 54.3 | 54.3 | [216] |
| **Canada** | 2022 | 8988 | 8988 | [217] |
| **Colombia** | 2012 | 148 | 148 | [42] |
| **Colombia** | 2021 | 34 | 34 | [218] |
| **Mexico** | 1998 | 30 | 30 | [219] |
| **Mexico** | 2006 | 400 | 400 | [220] |
| **Mexico** | 2011 | 300 | 300 | [221] |
| **Mexico** | 2016 | 173.6 | 173.6 | [222] |
| **Brazil** | 2003 | 900 | 854.5 | [223] |
| **Brazil** | 2005 | 809 |  | [224] |
| **Brazil** | 2011 | 264 | 1649.5 | [221] |
| **Brazil** | 2014 | 3035 |  | [225] |
| **Brazil** | 2016 | 1694 | 867.6 | [226] |
| **Brazil** | 2017 | 8.8 |  | [227] |
| **Brazil** | 2019 | 900 |  | [53] |
| **Tanzania** | 2020 | 2.9 | 2.9 | [228] |
| **Ghana** | 2008 | 103 | 103 | [229] |
| **Ghana** | 2019 | 484.56 | 484.56 | [230] |
| **Bolivia** | 2011 | 21 | 21 | [221] |
| **South Africa** | 2011 | 44.60 | 45.68 | [231] |
| **South Africa** | 2013 | 46.76 |  | [232] |
| **South Africa** | 2017 | 65.3 | 65.3 | [233] |
| **South Africa** | 2022 | 91.92 | 91.92 | [234] |
| **Argentina** | 2015 | 20.7 | 20.7 | [235] |
| **Argentina** | 2018 | 0.5059 | 11.25295 | [236] |
| **Argentina** | 2020 | 22 |  | [74] |
| **Nigeria** | 2009 | 18 | 18 | [237] |
| **Nigeria** | 2014 | 16 | 16 | [238] |
| **Nigeria** | 2019 | 468 | 240.6 | [198] |
| **Nigeria** | 2020 | 13.2 |  | [74] |
| **Nigeria** | 2021 | 4.6 | 4.6 | [239] |
| **Egypt** | 2001 | 14 | 14 | [240] |
| **Egypt** | 2009 | 700 | 700 | [241] |
| **Egypt** | 2015 | 61.3 | 61.3 | [242] |
| **Egypt** | 2016 | 67.5 | 157.925 | [243] |
| **Egypt** | 2018 | 182.8 |  | [244] |
| **Egypt** | 2019 | 377 |  | [245] |
| **Egypt** | 2020 | 4.4 |  | [246] |
| **Portugal** | 2003 | 6909 | 3039.367 | [247] |
| **Portugal** | 2004 | 651.1 |  | [248] |
| **Portugal** | 2005 | 1558 |  | [249] |
| **Portugal** | 2008 | 3078 | 3078 | [250] |
| **Portugal** | 2011 | 223 |  | [251] |
| **Portugal** | 2013 | 918.6 | 429.5333 | [252] |
| **Portugal** | 2015 | 147 |  | [253] |
| **Portugal** | 2017 | 130 | 130 | [254] |
| **Spain** | 1998 | 782 | 782 | [255] |
| **Spain** | 2001 | 86.2 | 1313.1 | [256] |
| **Spain** | 2005 | 2540 |  | [257] |
| **Spain** | 2006 | 3003 | 1187.5 | [258] |
| **Spain** | 2007 | 87 |  | [259] |
| **Spain** | 2008 | 1510 |  | [260] |
| **Spain** | 2009 | 150 |  | [261] |
| **Spain** | 2013 | 125.1 | 68.05 | [262] |
| **Spain** | 2015 | 11 |  | [263] |
| **Spain** | 2016 | 3349 | 3349 | [264] |
| **Slovakia** | 2002 | 892 | 657.5 | [190] |
| **Slovakia** | 2005 | 423 |  | [265] |
| **Slovakia** | 2009 | 426 | 426 | [266] |
| **Slovakia** | 2010 | 426 |  | [1] |
| **Slovakia** | 2012 | 519 | 519 | [1] |
| **Slovakia** | 2018 | 2484 | 1004.833 | [267] |
| **Slovakia** | 2019 | 401 |  | [268] |
| **Slovakia** | 2020 | 129.5 |  | [269] |
| **Germany** | 1993 | 39.39 | 39.39 | [270] |
| **Germany** | 1997 | 265 | 161.05 | [271] |
| **Germany** | 1998 | 57.1 |  | [272] |
| **Germany** | 2001 | 143.1 | 107.0333 | [198] |
| **Germany** | 2002 | 86 |  | [273] |
| **Germany** | 2003 | 92 |  | [273] |
| **Germany** | 2010 | 320 | 320 | [274] |
| **Germany** | 2012 | 800 | 581 | [274] |
| **Germany** | 2013 | 362 |  | [275] |
| **Germany** | 2016 | 377.7 | 192.3333 | [243] |
| **Germany** | 2019 | 159.3 |  | [245] |
| **Germany** | 2020 | 40 |  | [274] |
| **France** | 2001 | 29 | 29 | [276] |
| **France** | 2007 | 131 | 131 | [277] |
| **France** | 2011 | 426 | 547 | [278] |
| **France** | 2013 | 668 |  | [279] |
| **France** | 2016 | 412 | 144.4267 | [280] |
| **France** | 2019 | 3.28 |  | [281] |
| **France** | 2020 | 18 |  | [274] |
| **France** | 2021 | 75.1 | 75.1 | [282] |
| **Finland** | 2003 | 4340 | 4340 | [283] |
| **Finland** | 2006 | 12.5 | 17.1 | [284] |
| **Finland** | 2007 | 21.7 |  | [285] |
| **Finland** | 2012 | 63.1 | 63.1 | [286] |
| **Finland** | 2018 | 3731 | 1880.5 | [287] |
| **Finland** | 2020 | 30 |  | [274] |
| **Iran** | 2007 | 6525 | 4012.5 | [288] |
| **Iran** | 2010 | 1500 |  | [110] |
| **Iran** | 2011 | 920.8 | 793.2175 | [289] |
| **Iran** | 2012 | 8.27 |  | [290] |
| **Iran** | 2013 | 795 |  | [291] |
| **Iran** | 2015 | 1448.8 |  | [292] |
| **Iran** | 2017 | 2288.55 | 1129.815 | [293] |
| **Iran** | 2018 | 1775.2 |  | [294] |
| **Iran** | 2019 | 430 |  | [295] |
| **Iran** | 2020 | 25.51 |  | [296] |
| **Iran** | 2021 | 1700 | 1700 | [297] |
| **China** | 2001 | 57.8 | 195.9333 | [298] |
| **China** | 2002 | 190 |  | [299] |
| **China** | 2005 | 340 |  | [300] |
| **China** | 2006 | 4030 | 1679.767 | [301] |
| **China** | 2008 | 935 |  | [198] |
| **China** | 2010 | 74.3 |  | [302] |
| **China** | 2012 | 133.4 | 57.12667 | [303] |
| **China** | 2013 | 23.4 |  | [304] |
| **China** | 2014 | 14.58 |  | [305] |
| **China** | 2016 | 300.8 | 3351.4 | [306] |
| **China** | 2020 | 6402 |  | [307] |
| **India** | 1991 | 390 | 390 | [308] |
| **India** | 2001 | 31.6 | 1020.533 | [309] |
| **India** | 2003 | 30 |  | [310] |
| **India** | 2004 | 3000 |  | [311] |
| **India** | 2008 | 12.43 | 3061.143 | [312] |
| **India** | 2009 | 9136 |  | [313] |
| **India** | 2010 | 35 |  | [314] |
| **India** | 2012 | 15.43 | 41.7 | [315] |
| **India** | 2013 | 9.67 |  | [316] |
| **India** | 2014 | 100 |  | [317] |
| **India** | 2018 | 62.5 | 44.815 | [318] |
| **India** | 2019 | 27.13 |  | [318] |
| **Bangladesh** | 1996 | 220 | 138.34 | [319] |
| **Bangladesh** | 1999 | 56.68 |  | [320] |
| **Bangladesh** | 2001 | 42.5 | 26.58333 | [321] |
| **Bangladesh** | 2003 | 24.65 |  | [322] |
| **Bangladesh** | 2005 | 12.6 |  | [323] |
| **Bangladesh** | 2007 | 51.9 | 44.535 | [324] |
| **Bangladesh** | 2008 | 65.03 |  | [325] |
| **Bangladesh** | 2009 | 35.21 |  | [326] |
| **Bangladesh** | 2010 | 26 |  | [302] |
| **Bangladesh** | 2012 | 116 | 116 | [327] |
| **UK** | 1986 | 110 | 110 | [328] |
| **UK** | 1999 | 5266 | 5266 | [329] |
| **UK** | 2001 | 1845 | 1750.5 | [330] |
| **UK** | 2003 | 4466 |  | [328] |
| **UK** | 2004 | 220 |  | [331] |
| **UK** | 2005 | 471 |  | [1] |
| **UK** | 2006 | 130 | 1545 | [332] |
| **UK** | 2007 | 2960 |  | [333] |
| **UK** | 2012 | 1575 | 1575 | [334] |
| **UK** | 2016 | 89 | 89 | [335] |
| **Poland** | 1994 | 30 | 30 | [336] |
| **Poland** | 2001 | 16.45 | 16.925 | [337] |
| **Poland** | 2003 | 17.4 |  | [338] |
| **Poland** | 2008 | 22 | 576 | [339] |
| **Poland** | 2009 | 1130 |  | [340] |
| **Poland** | 2012 | 19.62 | 19.62 | [341] |
| **Poland** | 2019 | 6070 | 6070 | [342] |
| **USA** | 1989 | 7.63 | 7.63 | [343] |
| **USA** | 1993 | 363.37 | 363.37 | [344] |
| **USA** | 2001 | 1860 | 1260 | [345] |
| **USA** | 2002 | 660 |  | [346] |
| **USA** | 2006 | 900 | 555.6667 | [347] |
| **USA** | 2008 | 700 |  | [348] |
| **USA** | 2010 | 67 |  | [349] |
| **USA** | 2011 | 291 | 216.1 | [350] |
| **USA** | 2012 | 278 |  | [351] |
| **USA** | 2015 | 79.3 |  | [352] |
| **Chile** | 2001 | 391.1 | 184.525 | [353] |
| **Chile** | 2002 | 202 |  | [354] |
| **Chile** | 2004 | 70 |  | [355] |
| **Chile** | 2005 | 75 |  | [356] |
| **Chile** | 2011 | 68 | 107.3333 | [221] |
| **Chile** | 2012 | 213 |  | [357] |
| **Chile** | 2014 | 41 |  | [358] |
| **Chile** | 2019 | 16 | 16 | [359] |
| **Chile** | 2021 | 58.2 | 441.1 | [360] |
| **Chile** | 2022 | 824 |  | [361] |
| **Italy** | 1998 | 2035.3 | 2035.3 | [362] |
| **Italy** | 2004 | 691 | 691 | [363] |
| **Italy** | 2007 | 169 | 236.5 | [364] |
| **Italy** | 2009 | 304 |  | [365] |
| **Italy** | 2012 | 4358 | 1818 | [365] |
| **Italy** | 2014 | 194 |  | [366] |
| **Italy** | 2015 | 902 |  | [367] |
| **Italy** | 2017 | 200 | 200 | [125] |
| **Italy** | 2021 | 528 | 528 | [368] |
| **Czech Republic** | 2015 | 483 | 483 | [369] |
| **Czech Republic** | 2018 | 30 | 30 | [1] |
| **Korea** | 2001 | 788 | 407.25 | [370] |
| **Korea** | 2002 | 137 |  | [371] |
| **Korea** | 2004 | 573 |  | [372] |
| **Korea** | 2005 | 131 |  | [3] |
| **Korea** | 2011 | 145 | 80.945 | [373] |
| **Korea** | 2013 | 16.89 |  | [374] |
| **Korea** | 2016 | 704 | 641.45 | [375] |
| **Korea** | 2018 | 578.9 |  | [376] |
| **Nepal** | 2007 | 33 | 33 | [324] |
| **Cambodia** | 2009 | 26.36 | 26.36 | [377] |
| **Cambodia** | 2011 | 18 | 22.905 | [378] |
| **Cambodia** | 2012 | 27.81 |  | [379] |
| **Cambodia** | 2017 | 95 | 95 | [380] |
| **Japan** | 1989 | 216.1 | 216.1 | [381] |
| **Japan** | 2000 | 3675 | 3675 | [382] |
| **Japan** | 2006 | 0.33 | 15.06 | [381] |
| **Japan** | 2009 | 29.79 |  | [381] |
| **Japan** | 2018 | 921 | 361.5 | [383] |
| **Japan** | 2019 | 93.5 |  | [384] |
| **Japan** | 2020 | 70 |  | [74] |
| **Japan** | 2021 | 442 | 442 | [385] |
| **Vietnam** | 2005 | 41.7 | 525.85 | [386] |
| **Vietnam** | 2006 | 1010 |  | [387] |
| **Vietnam** | 2011 | 940 | 492.75 | [388] |
| **Vietnam** | 2015 | 45.5 |  | [389] |
| **Vietnam** | 2016 | 54.2 | 1169.733 | [390] |
| **Vietnam** | 2018 | 3390 |  | [391] |
| **Vietnam** | 2019 | 65 |  | [392] |
| **Thailand** | 1998 | 124 | 1562 | [393] |
| **Thailand** | 1999 | 3000 |  | [394] |
| **Thailand** | 2006 | 269 | 287.9375 | [395] |
| **Thailand** | 2007 | 167 |  | [396] |
| **Thailand** | 2008 | 711 |  | [397] |
| **Thailand** | 2010 | 4.75 |  | [398] |
| **Thailand** | 2011 | 64 | 64 | [399] |
| **Thailand** | 2017 | 1070.4 | 1070.4 | [400] |
| **Kazakhstan** | 2020 | 721 | 721 | [401] |
| **Qatar** | 2018 | 7.63 | 29.965 | [402] |
| **Qatar** | 2020 | 52.3 |  | [403] |
| **Sri Lanka** | 2004 | 0.84 | 0.84 | [404] |
| **Sri Lanka** | 2013 | 24 | 24 | [405] |
| **Sri Lanka** | 2020 | 3.6 | 3.6 | [406] |
| **Indonesia** | 2019 | 10.8 | 205.9 | [407] |
| **Indonesia** | 2020 | 401 |  | [408] |
| **KSA** | 2012 | 19 | 19 | [409] |
| **KSA** | 2021 | 6 | 13 | [410] |
| **KSA** | 2022 | 20 |  | [411] |
| **Jordan** | 2014 | 17.77 | 17.77 | [412] |
| **Malaysia** | 2011 | 57.05 | 57.05 | [413] |
| **Ecuador** | 2020 | 11.63 | 27.315 | [414] |
| **Ecuador** | 2022 | 43 |  | [415] |
| **Peru** | 1996 | 158 | 158 | [416] |
| **Sweden** | 2007 | 1128 | 1128 | [417] |
| **Switzerland** | 2006 | 50 | 50 | [418] |
| **Denmark** | 1997 | 917 | 917 | [419] |
| **Romania** | 2005 | 1020 | 1020 | [420] |
| **Romania** | 2009 | 295 | 295 | [421] |
| **Romania** | 2019 | 688 | 688 | [422] |
| **Serbia** | 2006 | 58.5 | 129.9667 | [423] |
| **Serbia** | 2009 | 310 |  | [424] |
| **Serbia** | 2010 | 21.4 |  | [425] |
| **Serbia** | 2012 | 92.21 | 92.21 | [426] |
| **Russia** | 2015 | 40 | 40 | [427] |
| **Mali** | 2016 | 1928.8 | 1928.8 | [180] |
| **Malawi** | 2018 | 39 | 22.055 | [428] |
| **Malawi** | 2019 | 5.11 |  | [429] |
| **Tanzania** | 2011 | 20.298 | 20.298 | [430] |
| **Morocco** | 2011 | 10.9 | 29.04 | [431] |
| **Morocco** | 2013 | 56.5 |  | [432] |
| **Morocco** | 2018 | 19.72 | 52.64 | [433] |
| **Morocco** | 2020 | 26.2 |  | [434] |
| **Morocco** | 2022 | 112.00 | 112 | [435] |
| **Australia** | 2003 | 721 | 1192.667 | [436] |
| **Australia** | 2005 | 1000 |  | [437] |
| **Australia** | 2011 | 1857 | 1857 | [438] |
| **Australia** | 2019 | 40 | 48.89333 | [115] |
| **Australia** | 2020 | 99.2 |  | [439] |
| **Ecuador** | 2015 | 7.48 | 104.24 | [107] |
| **Ecuador** | 2020 | 201 |  | [440] |
| **Ecuador** | 2022 | 115.8 | 115.8 | [440] |
| **Greece** | 2009 | 520 | 474.3333 | [441] |
| **Greece** | 2010 | 513 |  | [1] |
| **Greece** | 2014 | 1077 |  | [442] |
| **Greece** | 2015 | 1200 |  | [443] |
| **Pakistan** | 2008 | 35 | 35 | [444] |
| **Pakistan** | 2011 | 13.9 | 13.9 | [445] |
| **Pakistan** | 2018 | 15 | 15 | [446] |
| **Pakistan** | 2021 | 15.674 | 15.674 | [447] |
| **Turkey** | 2002 | 7.11 | 29.055 | [18] |
| **Turkey** | 2005 | 51 |  | [448] |
| **Turkey** | 2007 | 2488.4 | 1277 | [1] |
| **Turkey** | 2010 | 65.6 |  | [449] |
| **Turkey** | 2011 | 0.083 | 158.7915 | [450] |
| **Turkey** | 2015 | 317.5 |  | [451] |
| **Turkey** | 2018 | 660 | 340.5 | [1] |
| **Turkey** | 2020 | 21 |  | [452] |
| **Togo** | 2008 | 0.829 | 0.829 | [109] |
| **Botswana** | 2008 | 5 | 4.63 | [453] |
| **Botswana** | 2010 | 4.26 |  | [454] |
| **Botswana** | 2020 | 19.88 | 19.88 | [455] |
| **Ethiopia** | 2011 | 44.60 | 22.965 | [231] |
| **Ethiopia** | 2015 | 1.33 |  | [456] |
| **Ethiopia** | 2020 | 31.40 | 31.4 | [457] |
| **Nicaragua** | 2015 | 51 | 51 | [458] |
| **Uruguay** | 2018 | 3.62 | 3.62 | [459] |
| **Uruguay** | 2022 | 30.7 | 30.7 | [460] |
| **Croatia** | 2010 | 42 | 30.2 | [461] |
| **Croatia** | 2012 | 18.40 |  | [462] |
| **Croatia** | 2017 | 490 | 490 | [463] |
| **Croatia** | 2021 | 105 | 105 | [464] |

**Table S4**. The countries reported arsenic contamination in their soils during the past four decades. Data are collected from qualified articles as described in **Supplementary Methods**.

| **Country No.** | **Argentina** | **Contaminated Medium** |
| --- | --- | --- |
| **1** | Australia | Soil |
| **2** | Bangladesh | Soil |
| **3** | Bolivia | Soil |
| **4** | Botswana | Soil |
| **5** | Brazil | Soil |
| **6** | Cambodia | Soil |
| **7** | Canada | Soil |
| **8** | Chile | Soil |
| **9** | China | Soil |
| **10** | Colombia | Soil |
| **11** | Croatia | Soil |
| **12** | Czech Republic | Soil |
| **13** | Denmark | Soil |
| **14** | Ecuador | Soil |
| **15** | Egypt | Soil |
| **16** | Ethiopia | Soil |
| **17** | Finland | Soil |
| **18** | France | Soil |
| **19** | Germany | Soil |
| **20** | Ghana | Soil |
| **21** | Greece | Soil |
| **22** | India | Soil |
| **23** | Indonesia | Soil |
| **24** | Iran | Soil |
| **25** | Italy | Soil |
| **26** | Japan | Soil |
| **27** | Jordan | Soil |
| **28** | Kazakhstan | Soil |
| **29** | Korea | Soil |
| **30** | Malawi | Soil |
| **31** | Malaysia | Soil |
| **32** | Mali | Soil |
| **33** | Mexico | Soil |
| **34** | Morocco | Soil |
| **35** | Nepal | Soil |
| **36** | Nicaragua | Soil |
| **37** | Nigeria | Soil |
| **38** | Pakistan | Soil |
| **39** | Peru | Soil |
| **40** | Poland | Soil |
| **41** | Portugal | Soil |
| **42** | Qatar | Soil |
| **43** | Romania | Soil |
| **44** | Russia | Soil |
| **45** | Saudi Arabia | Soil |
| **46** | Serbia | Soil |
| **47** | Slovakia | Soil |
| **48** | Solomon Islands | Soil |
| **49** | South Africa | Soil |
| **50** | Spain | Soil |
| **51** | Sri Lanka | Soil |
| **52** | Sweden | Soil |
| **53** | Switzerland | Soil |
| **54** | Tanzania | Soil |
| **55** | Thailand | Soil |
| **56** | Togo | Soil |
| **57** | Turkey | Soil |
| **58** | United Kingdom | Soil |
| **59** | United States of America | Soil |
| **60** | Uruguay | Soil |
| **61** | Vietnam | Soil |

# Supplementary References

1. Medunić, G., Ž. Fiket, and M. Ivanić, *Arsenic Contamination Status in Europe, Australia, and Other Parts of the World*, in *Arsenic in Drinking Water and Food*, S. Srivastava, Editor. 2020, Springer Singapore: Singapore. p. 183-233.

2. Lee, J.-Y., J.-C. Choi, and K.-K. Lee, *Variations in heavy metal contamination of stream water and groundwater affected by an abandoned lead–zinc mine in Korea.* Environmental Geochemistry and Health, 2005. **27**(3): p. 237-257.

3. Lee, J.-S., et al., *Evaluation of human exposure to arsenic due to rice ingestion in the vicinity of abandoned Myungbong Au–Ag mine site, Korea.* Journal of Geochemical Exploration, 2008. **96**(2): p. 231-235.

4. Kim, K., et al., *Relations of As concentrations among groundwater, soil, and bedrock in Chungnam, Korea: Implications for As mobilization in groundwater according to the As-hosting mineral change.* Journal of Hazardous Materials, 2012. **199-200**: p. 25-35.

5. Park, J.-D., et al., *Arsenic levels in the groundwater of Korea and the urinary excretion among contaminated area.* Journal of Exposure Science & Environmental Epidemiology, 2016. **26**(5): p. 458-463.

6. Lee, J.-Y., J. Cha, and M. Raza, *Groundwater development, use, and its quality in Korea: tasks for sustainable use.* Water Policy, 2021. **23**(6): p. 1375-1387.

7. Das, D., et al., *Arsenic in groundwater in six districts of West Bengal, India.* Environmental Geochemistry and Health, 1996. **18**(1): p. 5-15.

8. Brammer, H. and P. Ravenscroft, *Arsenic in groundwater: A threat to sustainable agriculture in South and South-east Asia.* Environment International, 2009. **35**(3): p. 647-654.

9. Singh, S., et al., *Groundwater arsenic contamination and associated health risks in Bihar, India.* International Journal of Environmental Research, 2014. **8**(1): p. 49-60.

10. Rahman, M.M., et al., *Status of groundwater arsenic contamination in all 17 blocks of Nadia district in the state of West Bengal, India: A 23-year study report.* Journal of Hydrology, 2014. **518**: p. 363-372.

11. Dhillon, A.K., *Arsenic Contamination of India’s Groundwater: A Review and Critical Analysis*, in *Arsenic Water Resources Contamination: Challenges and Solutions*, A. Fares and S.K. Singh, Editors. 2020, Springer International Publishing: Cham. p. 177-205.

12. Chakraborti, D., et al. *Groundwater Arsenic Contamination in the Ganga River Basin: A Future Health Danger*. International Journal of Environmental Research and Public Health, 2018. **15**, DOI: 10.3390/ijerph15020180.

13. Parviainen, A., et al., *Arsenic in bedrock, soil and groundwater — The first arsenic guidelines for aggregate production established in Finland.* Earth-Science Reviews, 2015. **150**: p. 709-723.

14. Pokhrel, D., B.S. Bhandari, and T. Viraraghavan, *Arsenic contamination of groundwater in the Terai region of Nepal: An overview of health concerns and treatment options.* Environment International, 2009. **35**(1): p. 157-161.

15. Shrestha, R.R., et al., *Groundwater Arsenic Contamination, Its Health Impact and Mitigation Program in Nepal.* Journal of Environmental Science and Health, Part A, 2003. **38**(1): p. 185-200.

16. Dahal, B.M., et al., *Arsenic contamination of soils and agricultural plants through irrigation water in Nepal.* Environmental Pollution, 2008. **155**(1): p. 157-163.

17. Gyawali, T., et al., *Spatial and temporal distribution of arsenic contamination in groundwater of Nawalparasi-West, Nepal: an investigation with suggested countermeasures for South Asian Region.* Environmental Monitoring and Assessment, 2022. **194**(8): p. 582.

18. olak, M., *THE EFFECTS OF COLEMANITE DEPOSITS ON THE ARSENIC CONCENTRATIONS OF SOIL AND GROUNDWATER IN IGDEKY-EMET, KTAHYA, TURKEY.* Water Air and Soil Pollution, 2003. **149**: p. 127–143.

19. Gunduz, O., A. Baba, and H. Elpit, *Arsenic in groundwater in Western Anatolia, Turkey: a review.* Groundwater Quality Sustainability, 2012: p. 141.

20. Aksoy, N., C. Şimşek, and O. Gunduz, *Groundwater contamination mechanism in a geothermal field: A case study of Balcova, Turkey.* Journal of Contaminant Hydrology, 2009. **103**(1): p. 13-28.

21. Arslan, S., *Assessment of groundwater and soil quality for agricultural purposes in Kopruoren basin, Kutahya, Turkey.* Journal of African Earth Sciences, 2017. **131**: p. 1-13.

22. Simsek, C., *Assessment of naturally occurring arsenic contamination in the groundwater of Sarkisla Plain (Sivas/Turkey).* Environmental Earth Sciences, 2013. **68**(3): p. 691-702.

23. Papacostas, N.C., et al., *Geomorphic controls on groundwater arsenic distribution in the Mekong River Delta, Cambodia.* Geology, 2008. **36**(11): p. 891-894.

24. Luu, T.T.G., S. Sthiannopkao, and K.-W. Kim, *Arsenic and other trace elements contamination in groundwater and a risk assessment study for the residents in the Kandal Province of Cambodia.* Environment International, 2009. **35**(3): p. 455-460.

25. Chakraborti, D., et al., *Arsenic: occurrence in groundwater.* Encyclopedia of environmental health, 2011. **2**: p. 1e17.

26. Murphy, T., et al., *Groundwater Irrigation and Arsenic Speciation in Rice in Cambodia.* Journal of Health and Pollution, 2018. **8**(19).

27. Shaji, E., et al., *Arsenic contamination of groundwater: A global synopsis with focus on the Indian Peninsula.* Geoscience frontiers, 2021. **12**(3): p. 101079.

28. Polizzotto, M.L., et al., *Near-surface wetland sediments as a source of arsenic release to ground water in Asia.* Nature, 2008. **454**(7203): p. 505-8.

29. Guo, X.j., et al., *Arsenic contamination of groundwater and prevalence of arsenical dermatosis in the Hetao plain area, Inner Mongolia, China*, in *Molecular Mechanisms of Metal Toxicity and Carcinogenesis*, X. Shi, et al., Editors. 2001, Springer US: Boston, MA. p. 137-140.

30. Gan, Y., et al., *Hydrogeochemistry and arsenic contamination of groundwater in the Jianghan Plain, central China.* Journal of Geochemical Exploration, 2014. **138**: p. 81-93.

31. Zhang, L., et al., *Review of arsenic geochemical characteristics and its significance on arsenic pollution studies in karst groundwater, Southwest China.* Applied Geochemistry, 2017. **77**: p. 80-88.

32. He, X., et al., *Groundwater Arsenic and Fluoride and Associated Arsenicosis and Fluorosis in China: Occurrence, Distribution and Management.* Exposure and Health, 2020. **12**(3): p. 355-368.

33. Dilpazeer, F., et al., *A Comprehensive Review of the Latest Advancements in Controlling Arsenic Contaminants in Groundwater.* Water, 2023. **15**(3): p. 478.

34. Xu, N., et al., *Exposure risk of groundwater arsenic contamination from Huaihe River Plain, China.* Emerging Contaminants, 2022. **8**: p. 310-317.

35. Sonderegger, J.L. and T. Ohguchi, *Irrigation related arsenic contamination of a thin, alluvial aquifer, Madison River Valley, Montana, U.S.A.* Environmental Geology and Water Sciences, 1988. **11**(2): p. 153-161.

36. Frost, F., et al., *A seasonal study of arsenic in groundwater, Snohomish County, Washington, USA.* Environmental Geochemistry and Health, 1993. **15**(4): p. 209-214.

37. Ayotte, J.D., et al., *Factors affecting temporal variability of arsenic in groundwater used for drinking water supply in the United States.* Science of The Total Environment, 2015. **505**: p. 1370-1379.

38. Thomas, et al., *Natural Background and Anthropogenic Arsenic Enrichment in Florida Soils, Surface Water, and Groundwater: A Review with a Discussion on Public Health Risk.* International Journal of Environmental Research & Public Health, 2018.

39. Wang, S. and C.N. Mulligan, *Occurrence of arsenic contamination in Canada: sources, behavior and distribution.* Sci Total Environ, 2006. **366**(2-3): p. 701-21.

40. Alam, M.S., Y. Wu, and T. Cheng, *Silicate Minerals as a Source of Arsenic Contamination in Groundwater.* Water, Air, & Soil Pollution, 2014. **225**(11): p. 2201.

41. Adenova, D., et al., *Groundwater Quality and Potential Health Risk in Zhambyl Region, Kazakhstan.* Water, 2023. **15**(3): p. 482.

42. Alonso, D.L., et al., *Environmental occurrence of arsenic in Colombia: a review.* Environ Pollut, 2014. **186**: p. 272-81.

43. Alonso, D.L., et al., *Environmental occurrence of arsenic in Colombia: A review.* Environmental Pollution, 2014. **186**: p. 272-281.

44. Armienta, M.A., et al., *Arsenic Contamination of Groundwater at Zimapán, Mexiko.* Hydrogeology Journal, 1997. **5**(2): p. 39-46.

45. Camacho, L.M., et al., *Occurrence and treatment of arsenic in groundwater and soil in northern Mexico and southwestern USA.* Chemosphere, 2011. **83**(3): p. 211-225.

46. Armienta, M.A. and N. Segovia, *Arsenic and fluoride in the groundwater of Mexico.* Environmental Geochemistry and Health, 2008. **30**(4): p. 345-353.

47. Alarcón-Herrera, M.T., et al., *Co-occurrence of arsenic and fluoride in groundwater of semi-arid regions in Latin America: Genesis, mobility and remediation.* Journal of Hazardous Materials, 2013. **262**: p. 960-969.

48. Prieto García, F., et al., *Arsenic contamination in groundwater in Zimapan, Hidalgo, Mexico.* Desalination and Water Treatment, 2016. **57**(28): p. 13038-13047.

49. Mahlknecht, J., et al., *Hydrochemical controls on arsenic contamination and its health risks in the Comarca Lagunera region (Mexico): Implications of the scientific evidence for public health policy.* Science of The Total Environment, 2023. **857**: p. 159347.

50. Gonzalez Rodriguez, B., et al., *Arsenic contamination of rural community wells in Nicaragua: A review of two decades of experience.* Science of The Total Environment, 2019. **657**: p. 1441-1449.

51. Delgado Quezada, V., M. Altamirano Espinoza, and J. Bundschuh, *Arsenic in geoenvironments of Nicaragua: Exposure, health effects, mitigation and future needs.* Science of The Total Environment, 2020. **716**: p. 136527.

52. Bundschuh, J., et al., *Arsenic in Latin America: New findings on source, mobilization and mobility in human environments in 20 countries based on decadal research 2010-2020.* Critical Reviews in Environmental Science and Technology, 2020(6): p. 1-139.

53. Teixeira, M.C., et al., *Arsenic contamination assessment in Brazil – Past, present and future concerns: A historical and critical review.* Science of The Total Environment, 2020. **730**: p. 138217.

54. Mirlean, N. and A. Roisenberg, *The effect of emissions of fertilizer production on the environment contamination by cadmium and arsenic in southern Brazil.* Environmental Pollution, 2006. **143**(2): p. 335-340.

55. Ahoulé, D.G., et al., *Arsenic in African Waters: A Review.* Water, Air, & Soil Pollution, 2015. **226**(9): p. 302.

56. Mirlean, N., P. Baisch, and D. Diniz, *Arsenic in groundwater of the Paraiba do Sul delta, Brazil: An atmospheric source?* Science of The Total Environment, 2014. **482-483**: p. 148-156.

57. de Meyer, C.M.C., et al., *Hotspots of geogenic arsenic and manganese contamination in groundwater of the floodplains in lowland Amazonia (South America).* Science of The Total Environment, 2023. **860**: p. 160407.

58. Bretzler, A., et al., *Groundwater arsenic contamination in Burkina Faso, West Africa: Predicting and verifying regions at risk.* Science of The Total Environment, 2017. **584-585**: p. 958-970.

59. Somé, I.T., et al., *Arsenic levels in tube-wells water, food, residents' urine and the prevalence of skin lesions in Yatenga province, Burkina Faso.* Interdiscip Toxicol, 2012. **5**(1): p. 38-41.

60. Irunde, R., et al., *Arsenic in Africa: potential sources, spatial variability, and the state of the art for arsenic removal using locally available materials.* Groundwater for Sustainable Development, 2022. **18**: p. 100746.

61. Izah, S.C. and A.L. Srivastav, *Level of arsenic in potable water sources in Nigeria and their potential health impacts: A review.* Journal of Environmental Treatment Techniques, 2015. **3**(1): p. 15-24.

62. Garba, Z., C. Gimba, and A. Galadima, *Arsenic contamination of domestic water from northern Nigeria.* International Journal of Science and Technology, 2012. **2**(1): p. 55-60.

63. Egbinola, C.N. and A.C. Amanambu, *Groundwater contamination in Ibadan, south-west Nigeria.* SpringerPlus, 2014. **3**(1): p. 1-6.

64. Etim, E.U., *Occurrence and Distribution of Arsenic, Antimony and Selenium in Shallow Groundwater Systems of Ibadan Metropolis, Southwestern Nigerian.* Journal of Health and Pollution, 2017. **7**(13): p. 32-41.

65. Abiye, T.A. and P. Bhattacharya, *Arsenic concentration in groundwater: Archetypal study from South Africa.* Groundwater for Sustainable Development, 2019. **9**: p. 100246.

66. Nicolli, H.B., et al., *Groundwater contamination with arsenic and other trace elements in an area of the pampa, province of Córdoba, Argentina.* Environmental Geology and Water Sciences, 1989. **14**(1): p. 3-16.

67. Bundschuh, J., et al., *Groundwater arsenic in the Chaco-Pampean Plain, Argentina: case study from Robles county, Santiago del Estero Province.* Applied Geochemistry, 2004. **19**(2): p. 231-243.

68. Paoloni, J.D., M.E. Sequeira, and C.E. Fiorentino, *Mapping of arsenic content and distribution in groundwater in the southeast Pampa, Argentina.* Journal of Environmental Health, 2005. **67**(8).

69. Bhattacharya, P., et al., *Distribution and mobility of arsenic in the Río Dulce alluvial aquifers in Santiago del Estero Province, Argentina.* Science of The Total Environment, 2006. **358**(1): p. 97-120.

70. Francisca, F.M. and M.E. Carro Perez, *Assessment of natural arsenic in groundwater in Cordoba Province, Argentina.* Environmental Geochemistry and Health, 2009. **31**(6): p. 673-682.

71. O’Reilly, J., et al., *Arsenic contamination of natural waters in San Juan and La Pampa, Argentina.* Environmental Geochemistry and Health, 2010. **32**(6): p. 491-515.

72. Giménez, M.C., et al., *Assessment of heavy metals concentration in arsenic contaminated groundwater of the Chaco Plain, Argentina.* International Scholarly Research Notices, 2013. **2013**.

73. Alvarez, M.d.P. and E. Carol, *Geochemical occurrence of arsenic, vanadium and fluoride in groundwater of Patagonia, Argentina: Sources and mobilization processes.* Journal of South American Earth Sciences, 2019. **89**: p. 1-9.

74. Kayode, O.T., A.P. Aizebeokhai, and A.M. Odukoya, *Arsenic in agricultural soils and implications for sustainable agriculture.* IOP Conference Series: Earth and Environmental Science, 2021. **655**(1): p. 012081.

75. Torres, I. and H. Ishiga, *Assessment of the geochemical conditions for the release of Arsenic, Iron and Copper into groundwater in the coastal Aquifer at Yumigahama, Western Japan.* WIT Transactions on Ecology and the Environment, 2003. **65**.

76. Hossain, S., et al., *Redox processes and occurrence of arsenic in a volcanic aquifer system of Kumamoto Area, Japan.* Environmental Earth Sciences, 2016. **75**(9): p. 740.

77. Ahmad, A. and P. Bhattacharya, *Arsenic Contamination of Groundwater in Indus River Basin of Pakistan*, in *Groundwater of South Asia*, A. Mukherjee, Editor. 2018, Springer Singapore: Singapore. p. 393-403.

78. Farooqi, A., H. Masuda, and N. Firdous, *Toxic fluoride and arsenic contaminated groundwater in the Lahore and Kasur districts, Punjab, Pakistan and possible contaminant sources.* Environmental Pollution, 2007. **145**(3): p. 839-849.

79. Baig, J.A., et al., *Evaluation of arsenic and other physico-chemical parameters of surface and ground water of Jamshoro, Pakistan.* Journal of Hazardous Materials, 2009. **166**(2): p. 662-669.

80. Shahid, M., et al., *Arsenic Level and Risk Assessment of Groundwater in Vehari, Punjab Province, Pakistan.* Exposure and Health, 2018. **10**(4): p. 229-239.

81. Shahid, M., et al., *A meta-analysis of the distribution, sources and health risks of arsenic-contaminated groundwater in Pakistan.* Environmental Pollution, 2018. **242**: p. 307-319.

82. Ullah, Z., et al., *Arsenic contamination, water toxicity, source apportionment, and potential health risk in groundwater of Jhelum Basin, Punjab, Pakistan.* Biological Trace Element Research, 2023. **201**(1): p. 514-524.

83. Ayotte, J.D., et al., *Arsenic in Groundwater in Eastern New England:  Occurrence, Controls, and Human Health Implications.* Environmental Science & Technology, 2003. **37**(10): p. 2075-2083.

84. Fordyce, F.M., et al., *Soil metal/metalloid concentrations in the Clyde Basin, Scotland, UK: implications for land quality – CORRIGENDUM.* Earth and Environmental Science Transactions of The Royal Society of Edinburgh, 2020. **111**(1): p. 75-76.

85. Fytianos, K. and C. Christophoridis, *Nitrate, Arsenic and Chloride Pollution of Drinking Water in Northern Greece. Elaboration by Applying GIS.* Environmental Monitoring and Assessment, 2004. **93**(1): p. 55-67.

86. Kouras, A., I. Katsoyiannis, and D. Voutsa, *Distribution of arsenic in groundwater in the area of Chalkidiki, Northern Greece.* Journal of Hazardous Materials, 2007. **147**(3): p. 890-899.

87. Katsoyiannis, I.A. and A.A. Katsoyiannis, *Arsenic and Other Metal Contamination of Groundwaters in the Industrial Area of Thessaloniki, Northern Greece.* Environmental Monitoring and Assessment, 2006. **123**(1): p. 393-406.

88. Katsoyiannis, I.A., et al., *Arsenic speciation and uranium concentrations in drinking water supply wells in Northern Greece: Correlations with redox indicative parameters and implications for groundwater treatment.* Science of The Total Environment, 2007. **383**(1): p. 128-140.

89. Aloupi, M., et al., *Influence of geology on arsenic concentrations in ground and surface water in central Lesvos, Greece.* Environmental Monitoring and Assessment, 2009. **151**(1): p. 383-396.

90. Papastergios, G., et al., *Arsenic Background Concentrations in Surface Soils of Kavala Area, Northern Greece.* Water, Air, & Soil Pollution, 2010. **209**(1): p. 323-331.

91. Zkeri, E., M. Aloupi, and P. Gaganis, *Seasonal and spatial variation of arsenic in groundwater in a rhyolithic volcanic area of Lesvos Island, Greece.* Environmental monitoring and assessment, 2018. **190**: p. 1-20.

92. Katsoyiannis, I.A., M. Mitrakas, and A.I. Zouboulis, *Arsenic occurrence in Europe: Emphasis in Greece and description of the applied full-scale treatment plants.* Desalination and Water Treatment, 2015. **54**(8): p. 2100-2107.

93. Zkeri, E., M. Aloupi, and P. Gaganis, *Natural Occurrence of Arsenic in Groundwater from Lesvos Island, Greece.* Water, Air, & Soil Pollution, 2015. **226**(9): p. 294.

94. Heinrichs, G. and P. Udluft, *Natural arsenic in Triassic rocks: A source of drinking-water contamination in Bavaria, Germany.* Hydrogeology Journal, 1999. **7**(5): p. 468-476.

95. Krüger, T., et al., *In situ remediation of arsenic at a highly contaminated site in Northern Germany.* IAHS publication, 2008. **324**: p. 118.

96. Weiske, A., et al., *Enhanced Arsenic Mobility in a Dystrophic Water Reservoir System After Acidification Recovery.* Water, Air, & Soil Pollution, 2017. **228**(8): p. 285.

97. Mehrdadi, N., et al., *Monitoring the arsenic concentration in groundwater resources, case study: Ghezel ozan water basin, Kurdistan, Iran.* Asian journal of chemistry, 2009. **21**(1): p. 446-50.

98. Keshavarzi, B., et al., *The Source of Natural Arsenic Contamination in Groundwater, West of Iran.* Water Quality, Exposure and Health, 2011. **3**(3): p. 135-147.

99. Pazand, K. and A.R. Javanshir, *Hydrogeochemistry and arsenic contamination of groundwater in the Rayen area, southeastern Iran.* Environmental Earth Sciences, 2013. **70**(6): p. 2633-2644.

100. Hamidian, A.H., et al., *Spatial distribution of arsenic in groundwater of Iran, a review.* Journal of Geochemical Exploration, 2019. **201**: p. 88-98.

101. Rahnamarad, J., R. Derakhshani, and A. Abbasnejad, *Data on arsenic contamination in groundwater of Rafsanjan plain, Iran.* Data in Brief, 2020. **31**: p. 105772.

102. Islam, M.R., R. Salminen, and P.W. Lahermo, *Arsenic and other toxic elemental contamination of groundwater, surface water and soil in Bangladesh and its possible effects on human health.* Environmental Geochemistry and Health, 2000. **22**(1): p. 33-53.

103. Chakraborti, D., et al., *Status of groundwater arsenic contamination in Bangladesh: A 14-year study report.* Water Research, 2010. **44**(19): p. 5789-5802.

104. Acharya, R., A.G.C. Nair, and A.V.R. Reddy, *Speciation and instrumental neutron activation analysis for arsenic in water samples.* Journal of Radioanalytical & Nuclear Chemistry, 2009. **281**(2): p. 279-282.

105. Jiang, J.-Q., et al., *Arsenic Contaminated Groundwater and Its Treatment Options in Bangladesh.* International Journal of Environmental Research and Public Health, 2013. **10**(1): p. 18-46.

106. Cumbal, L., et al., *Arsenic in geothermal sources at the north-central Andean region of Ecuador: concentrations and mechanisms of mobility.* Environmental Earth Sciences, 2010. **61**: p. 299-310.

107. Guanoluisa, et al., *Arsenic in rice agrosystems (water, soil and rice plants) in Guayas and Los Rios provinces, Ecuador.* Science of the Total Environment, 2016.

108. Buamah, R., B. Petrusevski, and J. Schippers, *Presence of arsenic, iron and manganese in groundwater within the gold-belt zone of Ghana.* Journal of Water Supply: Research and Technology—AQUA, 2008. **57**(7): p. 519-529.

109. Rezaieboroon, M.H., K. Gnandi, and K.T.M. Folly, *Presence and Distribution of Toxic Trace Elements in Water and Sediments of the Southern Togo Rivers Watershed, West Africa.* Fresenius Environmental Bulletin, 2011. **20**(7): p. 1853-1865.

110. Zandsalimi, S., N. Karimi, and A. Kohandel, *Arsenic in soil, vegetation and water of a contaminated region.* International Journal of Environmental Science & Technology, 2011. **8**(2): p. 331-338.

111. Ármannsson, H. *Monitoring the effect of geothermal effluent from the Krafla and Bjarnarflag power plants on groundwater in the lake Mývatn area, Iceland, with particular reference to natural tracers*. in *Proceedings World Geothermal Congress*. 2005.

112. Svensson, M., *Mobilisation of geogenic arsenic into groundwater in Västerbotten County, Sweden.* Department of Earth Sciences. Uppsala University, Villavägen, 2007. **16**(75236): p. 20.

113. Kitterød, N.-O., et al., *Hydrogeology and groundwater quality in the Nordic and Baltic countries.* Hydrology Research, 2022. **53**(7): p. 958-982.

114. Kozyatnyk, I., et al., *Fractionation and size-distribution of metal and metalloid contaminants in a polluted groundwater rich in dissolved organic matter.* Journal of Hazardous Materials, 2016. **318**: p. 194-202.

115. Smith, E., et al., *Arsenic in Australian environment: an overview.* Journal of Environmental Science and Health, Part A, 2003. **38**(1): p. 223-239.

116. Appleyard, S., et al., *Groundwater acidification caused by urban development in Perth, Western Australia: source, distribution, and implications for management.* Soil Research, 2004. **42**(6): p. 579-585.

117. Pfeifer, H.-R., et al., *Natural arsenic-contamination of surface and ground waters in Southern Switzerland (Ticino).* Bull. Appl. Geol, 2002. **7**(1): p. 81-103.

118. González-A, Z.I., et al., *Natural enrichment of arsenic in a minerotrophic peatland (Gola di Lago, Canton Ticino, Switzerland), and implications for the treatment of contaminated waters*. 2005: Natural enrichment of arsenic in a minerotrophic peatland (Gola di Lago, Canton Ticino, Switzerland), and implications for the treatment of contaminated waters.

119. Daniele, L., *Distribution of arsenic and other minor trace elements in the groundwater of Ischia Island (southern Italy).* Environmental Geology, 2004. **46**(1): p. 96-103.

120. Sellerino, M., G. Forte, and D. Ducci, *Identification of the natural background levels in the Phlaegrean fields groundwater body (Southern Italy).* Journal of Geochemical Exploration, 2019. **200**: p. 181-192.

121. Preziosi, E., G. Giuliano, and R. Vivona, *Natural background levels and threshold values derivation for naturally As, V and F rich groundwater bodies: a methodological case study in Central Italy.* Environmental Earth Sciences, 2010. **61**: p. 885-897.

122. Petrini, R., et al., *Natural arsenic contamination in waters from the Pesariis village, NE Italy.* Environmental Earth Sciences, 2011. **62**(3): p. 481-491.

123. Carraro, A., et al., *Arsenic anomalies in shallow Venetian Plain (Northeast Italy) groundwater.* Environmental Earth Sciences, 2013. **70**(7): p. 3067-3084.

124. Peña Reyes, F.A., et al., *Hydrogeochemical overview and natural arsenic occurrence in groundwater from alpine springs (upper Valtellina, Northern Italy).* Journal of Hydrology, 2015. **529**: p. 1530-1549.

125. Ghezzi, L., et al., *Arsenic Contamination in Groundwater, Soil and the Food-Chain: Risk Management in a Densely Populated Area (Versilia Plain, Italy).* Applied Sciences, 2023. **13**(9): p. 5446.

126. Parrone, D., et al., *Arsenic-fluoride co-contamination in groundwater: Background and anomalies in a volcanic-sedimentary aquifer in central Italy.* Journal of Geochemical Exploration, 2020. **217**: p. 106590.

127. Sappa, G., et al., *Assessment of arsenic mobility in a shallow aquifer from Bevera Valley Basin (Northern Italy).* Arabian Journal of Geosciences, 2019. **12**(22): p. 678.

128. Ersbøll, A.K., et al., *Low-level exposure to arsenic in drinking water and incidence rate of stroke: A cohort study in Denmark.* Environment International, 2018. **120**: p. 72-80.

129. Kiurski-Milošević, J., et al., *Groundwater arsenic contamination in Zrenjanin area.* ECOLOGY OF URBAN AREAS 2013, 2013: p. 243.

130. Kristoforović-Ilić, M.J., et al., *Arsenic contamination in environment in the region of Vojvodina.* Cent Eur J Public Health, 2009. **17**(3): p. 152-157.

131. Djokic, L.S., et al. *Origin of arsenic in drinking waters in the West Backa district of Serbia*. in *Water Treatment Technologies for the Removal of High-Toxicity Pollutants*. 2010. Springer.

132. Kiurski-Milosević, J.Ž., M.B. Vojinović-Miloradov, and N.M. Ralević, *Fuzzy model for determination and assessment of groundwater quality in the city of Zrenjanin, Serbia.* Hemijska industrija, 2015. **69**(1): p. 17-28.

133. Jovanovic, D., et al., *Arsenic occurrence in drinking water supply systems in ten municipalities in Vojvodina Region, Serbia.* Environmental Research, 2011. **111**(2): p. 315-318.

134. Agbaba, J., et al., *Water Supply Systems for Settlements with Arsenic-Contaminated Groundwater&mdash;Making the Right Choice.* Applied Sciences, 2023. **13**(17): p. 9557.

135. Devic, G., D. Djordjevic, and S. Sakan, *Natural and anthropogenic factors affecting the groundwater quality in Serbia.* Science of The Total Environment, 2014. **468-469**: p. 933-942.

136. Ulniković, V.P. and S.M. Kurilić, *Heavy metal and metalloid contamination and health risk assessment in spring water on the territory of Belgrade City, Serbia.* Environmental Geochemistry and Health, 2020. **42**(11): p. 3731-3751.

137. Ilić, I., M. Puharić, and D. Ilić, *Groundwater Quality Assessment and Prediction of Spatial Variations in the Area of the Danube River Basin (Serbia).* Water, Air, & Soil Pollution, 2021. **232**(3): p. 117.

138. Ferreccio, C., et al., *Lung Cancer and Arsenic Concentrations in Drinking Water in Chile.* Epidemiology, 2000. **11**(6): p. 673-679.

139. Leybourne, M.I. and E.M. Cameron, *Source, transport, and fate of rhenium, selenium, molybdenum, arsenic, and copper in groundwater associated with porphyry–Cu deposits, Atacama Desert, Chile.* Chemical Geology, 2008. **247**(1): p. 208-228.

140. Elvira Hernández-García, M. and E. Custodio, *Natural baseline quality of Madrid Tertiary Detrital Aquifer groundwater (Spain): a basis for aquifer management.* Environmental Geology, 2004. **46**(2): p. 173-188.

141. García-Sánchez, A., A. Moyano, and P. Mayorga, *High arsenic contents in groundwater of central Spain.* Environmental Geology, 2005. **47**(6): p. 847-854.

142. Gómez, J.J., J. Lillo, and B. Sahún, *Naturally occurring arsenic in groundwater and identification of the geochemical sources in the Duero Cenozoic Basin, Spain.* Environmental Geology, 2006. **50**(8): p. 1151-1170.

143. Mayorga, P., et al., *Temporal variation of arsenic and nitrate content in groundwater of the Duero River Basin (Spain).* Physics and Chemistry of the Earth, Parts A/B/C, 2013. **58-60**: p. 22-27.

144. Navarro, A., X. Font, and M. Viladevall, *Geochemistry and groundwater contamination in the La Selva geothermal system (Girona, Northeast Spain).* Geothermics, 2011. **40**(4): p. 275-285.

145. Ventura-Houle, R., X. Font, and L. Heyer, *Groundwater arsenic contamination and their variations on episode of drought: Ter River delta in Catalonia, Spain.* Applied Water Science, 2018. **8**(5): p. 128.

146. Giménez-Forcada, E. and P.L. Smedley, *Geological factors controlling occurrence and distribution of arsenic in groundwaters from the southern margin of the Duero Basin, Spain.* Environmental Geochemistry and Health, 2014. **36**(6): p. 1029-1047.

147. Charlet, L., et al., *Risk of arsenic transfer to a semi-confined aquifer and the effect of water level fluctuation in North Mortagne, France at a former industrial site.* Science of The Total Environment, 2001. **277**(1): p. 133-147.

148. Tiouiouine, A., et al., *Determining the Relevant Scale to Analyze the Quality of Regional Groundwater Resources While Combining Groundwater Bodies, Physicochemical and Biological Databases in Southeastern France.* Water, 2020. **12**(12): p. 3476.

149. Ebengue Atega, P.L., et al., *Development and implementation of a multi-criteria aggregation operator to estimate the contributions of the natural geochemical background and anthropogenic inputs in groundwater in former mining regions: An application to arsenic and antimony in the Gardon river watershed (southern France).* Science of The Total Environment, 2022. **814**: p. 151936.

150. van Wendel de Joode, B., et al., *Manganese concentrations in drinking water from villages near banana plantations with aerial mancozeb spraying in Costa Rica: Results from the Infants' Environmental Health Study (ISA).* Environmental Pollution, 2016. **215**: p. 247-257.

151. Kunz, et al., *Treatment of arsenic-contaminated water using in-line electrolysis, co-precipitation and filtration in Costa Rica.* Water science & technology: Water supply, 2018.

152. Gurzau, E.S. and A.E. Gurzau, *Arsenic in drinking water from groundwater in Transylvania, Romania: an overview.* Arsenic Exposure & Health Effects IV, 2001.

153. Rowland, H.A.L., et al., *Geochemistry and arsenic behaviour in groundwater resources of the Pannonian Basin (Hungary and Romania).* Applied Geochemistry, 2011. **26**(1): p. 1-17.

154. Berg, M., et al., *Arsenic Contamination of Groundwater and Drinking Water in Vietnam:  A Human Health Threat.* Environmental Science & Technology, 2001. **35**(13): p. 2621-2626.

155. Berg, M., et al., *Hydrological and sedimentary controls leading to arsenic contamination of groundwater in the Hanoi area, Vietnam: The impact of iron-arsenic ratios, peat, river bank deposits, and excessive groundwater abstraction.* Chemical Geology, 2008. **249**(1): p. 91-112.

156. Phuong, N.M., et al., *Arsenic contamination in groundwater and its possible sources in Hanam, Vietnam.* Environmental Monitoring and Assessment, 2012. **184**(7): p. 4501-4515.

157. Winkel, L.H., et al., *Arsenic pollution of groundwater in Vietnam exacerbated by deep aquifer exploitation for more than a century.* Proceedings of the National Academy of Sciences, 2011. **108**(4): p. 1246-1251.

158. Postma, D., et al., *Groundwater arsenic concentrations in Vietnam controlled by sediment age.* Nature Geoscience, 2012. **5**(9): p. 656-661.

159. Huang, Y., et al., *Arsenic contamination of groundwater and agricultural soil irrigated with the groundwater in Mekong Delta, Vietnam.* Environmental Earth Sciences, 2016. **75**(9): p. 757.

160. Pham, L.H., et al., *Arsenic and other trace elements in groundwater and human urine in Ha Nam province, the Northern Vietnam: contamination characteristics and risk assessment.* Environmental Geochemistry and Health, 2017. **39**(3): p. 517-529.

161. Pham, H.V., T.K.T. Pham, and V.N. Dao, *Arsenic contamination in groundwater in the Red river delta, Vietnam-a review.* Vietnam Journal of Science, Technology and Engineering, 2018. **60**(1): p. 23-27.

162. Rahman, M.M., R. Naidu, and P. Bhattacharya, *Arsenic contamination in groundwater in the Southeast Asia region.* Environmental Geochemistry and Health, 2009. **31**(1): p. 9-21.

163. Chanpiwat, P., et al., *Contamination by arsenic and other trace elements of tube-well water along the Mekong River in Lao PDR.* Environmental Pollution, 2011. **159**(2): p. 567-576.

164. Brindha, K., et al., *Geochemical Characteristics and Groundwater Quality in the Vientiane Plain, Laos.* Exposure and Health, 2017. **9**(2): p. 89-104.

165. Cha, Y., et al., *Bayesian modeling approach for characterizing groundwater arsenic contamination in the Mekong River basin.* Chemosphere, 2016. **143**: p. 50-56.

166. Wattanasen, K., et al., *An integrated geophysical study of arsenic contaminated area in the peninsular Thailand.* Environmental Geology, 2006. **51**(4): p. 595-608.

167. Wongsasuluk, P., et al., *Heavy metal contamination and human health risk assessment in drinking water from shallow groundwater wells in an agricultural area in Ubon Ratchathani province, Thailand.* Environmental Geochemistry and Health, 2014. **36**(1): p. 169-182.

168. Wongsasuluk, P., et al., *Using urine as a biomarker in human exposure risk associated with arsenic and other heavy metals contaminating drinking groundwater in intensively agricultural areas of Thailand.* Environmental Geochemistry and Health, 2018. **40**(1): p. 323-348.

169. Santha, N., S. Sangkajan, and S. Saenton, *Arsenic Contamination in Groundwater and Potential Health Risk in Western Lampang Basin, Northern Thailand.* Water, 2022. **14**(3): p. 465.

170. Nilkarnjanakul, W., P. Watchalayann, and S. Chotpantarat, *Urinary arsenic and health risk of the residents association in contaminated-groundwater area of the urbanized coastal aquifer, Thailand.* Chemosphere, 2023. **313**: p. 137313.

171. Van de Wauw, J., R. Evens, and L. Machiels, *Are groundwater overextraction and reduced infiltration contributing to arsenic related health problems near the Marlin mine (Guatemala).* Ghent, Belgium: University of Ghent, 2010.

172. Lotter, J.T., et al., *Groundwater arsenic in Chimaltenango, Guatemala.* Journal of Water and Health, 2013. **12**(3): p. 533-542.

173. Fetisova, N. *Arsenic speciation and sorption in acid mine drainage and the polluted water of the Kosva river basin, Russia*. in *IOP Conference Series: Earth and Environmental Science*. 2022. IOP Publishing.

174. Bage, G., et al., *Arsenic, from a Woeful Environmental Hazard to a Wishful Exploration Tool: A Case Study of Arsenic Contaminated Groundwater of Chakariya Area, Singrauli District, Madhya Pradesh, India*, in *Innovations in Sustainable Mining: Balancing Environment, Ecology and Economy*, K. Randive, S. Pingle, and A. Agnihotri, Editors. 2021, Springer International Publishing: Cham. p. 115-138.

175. Huntsman-Mapila, P., et al., *Characterization of arsenic occurrence in the water and sediments of the Okavango Delta, NW Botswana.* Applied Geochemistry, 2006. **21**(8): p. 1376-1391.

176. Huntsman-Mapila, P., et al., *Arsenic Distribution and Geochemistry in Island Groundwater of the Okavango Delta in Botswana*, in *Sustaining Groundwater Resources: A Critical Element in the Global Water Crisis*, J.A.A. Jones, Editor. 2011, Springer Netherlands: Dordrecht. p. 55-67.

177. Akoachere, R.A., et al., *Trace metals in groundwater of the south eastern piedmont region of Mount Cameroon: quantification and health risk assessment.* Open Access Library Journal, 2019. **6**(4): p. 1-22.

178. Kouassy Kalédjé, P.S., et al., *Groundwater quality assessment in the catchment area of Kadey (East-Cameroon): water quality index approach.* Sustainable Water Resources Management, 2023. **9**(5): p. 137.

179. Verma, S., et al. *Solute chemistry and groundwater arsenic enrichment in southern part of Brahmaputra River Basin, India, adjacent to Indo-Burmese ranges*. in *Arsenic Research and Global Sustainability: Proceedings of the Sixth International Congress on Arsenic in the Environment (As2016), June 19-23, 2016, Stockholm, Sweden*. 2016. CRC Press.

180. Bokar, H., et al., *Geogenic influence and impact of mining activities on water soil and plants in surrounding areas of Morila Mine, Mali.* Journal of Geochemical Exploration, 2020. **209**: p. 106429.

181. Sidibe, A.M. and L. Xueyu, *Heavy metals and nitrate to validate groundwater sensibility assessment based on DRASTIC models and GIS: Case of the upper Niger and the Bani basin in Mali.* Journal of African Earth Sciences, 2018. **147**: p. 199-210.

182. Pritchard, M., T. Mkandawire, and J. O’neill, *Assessment of groundwater quality in shallow wells within the southern districts of Malawi.* Physics and Chemistry of the Earth, Parts A/B/C, 2008. **33**(8-13): p. 812-823.

183. Rivett, M., et al., *Arsenic occurrence in Malawi groundwater.* Journal of Applied Sciences and Environmental Management, 2018. **22**(11): p. 1807–1816-1807–1816.

184. Ligate, F., et al., *Geogenic contaminants and groundwater quality around Lake Victoria goldfields in northwestern Tanzania.* Chemosphere, 2022. **307**: p. 135732.

185. Stachnik, Ł., et al., *Arsenic pollution in Quaternary sediments and water near a former gold mine.* Scientific Reports, 2020. **10**(1): p. 18458.

186. Winkel, L., et al., *Hydrogeological survey assessing arsenic and other groundwater contaminants in the lowlands of Sumatra, Indonesia.* Applied Geochemistry, 2008. **23**(11): p. 3019-3028.

187. Rochaddi, B., A. Sabdono, and M. Zainuri, *Heavy Metal (As and Hg) contamination of shallow groundwater in the coastal areas of Pati and Rembang, Central Java, Indonesia.* IOP Conference Series: Earth and Environmental Science, 2020. **530**(1): p. 012035.

188. McGrory, E., E. Holian, and L. Morrison, *Assessment of groundwater processes using censored data analysis incorporating non-detect chemical, physical, and biological data.* Journal of Contaminant Hydrology, 2020. **235**: p. 103706.

189. Al-Makishah, N.H., M.A. Taleb, and M.A. Barakat, *Arsenic bioaccumulation in arsenic-contaminated soil: a review.* Chemical Papers, 2020. **74**(9): p. 2743-2757.

190. Rapant, S., Z. Dietzová, and S. Cicmanová, *Environmental and health risk assessment in abandoned mining area, Zlata Idka, Slovakia.* Environmental Geology, 2006. **51**(3): p. 387-397.

191. Hiller, E., et al., *Arsenic and antimony contamination of waters, stream sediments and soils in the vicinity of abandoned antimony mines in the Western Carpathians, Slovakia.* Applied Geochemistry, 2012. **27**(3): p. 598-614.

192. Schmidt, T., et al., *Environmental risk from the contamination of groundwater with toxic elements in the Slovak Republic and Bratislava region.* Carpathian Journal of Earth and Environmental Sciences, 2017. **12**: p. 541-548.

193. George, C.M., et al., *Arsenic exposure in drinking water: an unrecognized health threat in Peru.* Bulletin of the World Health Organization, 2014. **92**: p. 565-572.

194. Makaya, E. and M.T. Maphosa, *Spatial analysis of groundwater quality in Bulawayo, Zimbabwe: Geogenic and anthropogenic contributions.* Physics and Chemistry of the Earth, Parts A/B/C, 2023. **131**: p. 103436.

195. Al Kuisi, M., et al., *Hydrogeochemistry of groundwater from karstic limestone aquifer highlighting arsenic contamination: case study from Jordan.* Arabian Journal of Geosciences, 2015. **8**(11): p. 9699-9720.

196. Alansi, R.Q., et al., *Determination of Heavy Metals in Groundwater Around Al-Buraihi Sewage Station in Taiz City, Yemen.* J Health Pollut, 2021. **11**(30): p. 210604.

197. Stuyfzand, P.J. and M. Bonte, *A critical review of arsenic occurrence, fate and transport in natural and modified groundwater systems in The Netherlands.* Applied Geochemistry, 2023. **150**: p. 105596.

198. Abd Wahil, M.S., et al., *Health Risk Assessment on High Groundwater Arsenic Concentration among Adult and Children in Beranang Subdistrict, Malaysia.* Pertanika Journal of Science & Technology, 2020. **28**(3).

199. Machado, I., V. Bühl, and N. Mañay, *Total arsenic and inorganic arsenic speciation in groundwater intended for human consumption in Uruguay: Correlation with fluoride, iron, manganese and sulfate.* Science of The Total Environment, 2019. **681**: p. 497-502.

200. Embaby, A. and M. Redwan, *Sources and behavior of trace elements in groundwater in the South Eastern Desert, Egypt.* Environmental Monitoring and Assessment, 2019. **191**(11): p. 686.

201. Andrade, A.I.A.S.S. and T.Y. Stigter, *The distribution of arsenic in shallow alluvial groundwater under agricultural land in central Portugal: Insights from multivariate geostatistical modeling.* Science of The Total Environment, 2013. **449**: p. 37-51.

202. Cabral Pinto, M.M.S., et al., *An Inter-disciplinary Approach to Evaluate Human Health Risks Due to Long-Term Exposure to Contaminated Groundwater Near a Chemical Complex.* Exposure and Health, 2020. **12**(2): p. 199-214.

203. Costa, M.R., et al., *In vitro toxicity of arsenic rich waters from an abandoned gold mine in northeast Portugal.* Environmental Research, 2021. **202**: p. 111683.

204. Neiva, A.M.R., et al., *Assessment of metal and metalloid contamination in the waters and stream sediments around the abandoned uranium mine area from Mortórios, central Portugal.* Journal of Geochemical Exploration, 2019. **202**: p. 35-48.

205. Drahota, P., et al., *Mineralogical and geochemical controls of arsenic speciation and mobility under different redox conditions in soil, sediment and water at the Mokrsko-West gold deposit, Czech Republic.* Science of The Total Environment, 2009. **407**(10): p. 3372-3384.

206. MacLean, K. and W. Langille, *Arsenic in orchard and potato soils and plant tissue.* Plant and Soil, 1981. **61**: p. 413-418.

207. Zoltai, S., *Distribution of base metals in peat near a smelter at Flin Flon, Manitoba.* Water, Air, and Soil Pollution, 1988. **37**: p. 217-228.

208. Wang, S. and C.N. Mulligan, *Occurrence of arsenic contamination in Canada: Sources, behavior and distribution.* Science of The Total Environment, 2006. **366**(2): p. 701-721.

209. Ollson, C.A., *Arsenic Contamination of the Terrestrial and Freshwater Environment Impacted by Gold Mining Operations Yellowknife, Northwest Territories*. 2002: National Library of Canada= Bibliothèque nationale du Canada, Ottawa.

210. Wang, S. and C.N. Mulligan, *Occurrence of arsenic contamination in Canada: sources, behavior and distribution.* Science of the total Environment, 2006. **366**(2-3): p. 701-721.

211. Rasmussen, P., K. Subramanian, and B. Jessiman, *A multi-element profile of house dust in relation to exterior dust and soils in the city of Ottawa, Canada.* Science of the total environment, 2001. **267**(1-3): p. 125-140.

212. Francesconi, K., et al., *Arsenic species in an arsenic hyperaccumulating fern, Pityrogramma calomelanos: a potential phytoremediator of arsenic-contaminated soils.* Science of the Total Environment, 2002. **284**(1-3): p. 27-35.

213. Zheng, J., et al., *Speciation of arsenic in water, sediment, and plants of the Moira watershed, Canada, using HPLC coupled to high resolution ICP–MS.* Analytical and Bioanalytical Chemistry, 2003. **377**: p. 14-24.

214. Lambert, T.W. and S. Lane, *Lead, arsenic, and polycyclic aromatic hydrocarbons in soil and house dust in the communities surrounding the Sydney, Nova Scotia, tar ponds.* Environmental health perspectives, 2004. **112**(1): p. 35-41.

215. Ampiah-Bonney, R.J., J. Tyson, and G. Lanza, *Phytoextraction of arsenic from soil by Leersia oryzoides.* International journal of phytoremediation, 2007. **9**(1): p. 31-40.

216. Amuno, S., et al., *Comparative study of arsenic toxicosis and ocular pathology in wild muskrats (Ondatra zibethicus) and red squirrels (Tamiasciurus hudsonicus) breeding in arsenic contaminated areas of Yellowknife, Northwest Territories (Canada).* Chemosphere, 2020. **248**: p. 126011.

217. Munford, K.E., et al., *How arsenic contamination influences downslope wetland plant and microbial community structure and function.* Science of The Total Environment, 2023. **876**: p. 162839.

218. Donado, E.P., et al., *Soil contamination in Colombian playgrounds: effects of vehicles, construction, and traffic.* Environmental Science and Pollution Research, 2021. **28**: p. 166-176.

219. Rosas, I., et al., *Arsenic concentrations in water, soil, milk and forage in Comarca Lagunera, Mexico.* Water, Air, and Soil Pollution, 1999. **112**: p. 133-149.

220. Ongley, L.K., et al., *Arsenic in the soils of Zimapán, Mexico.* Environmental Pollution, 2007. **145**(3): p. 793-799.

221. Bundschuh, J., et al., *Arsenic in the human food chain: the Latin American perspective.* Science of The Total Environment, 2012. **429**: p. 92-106.

222. Ruíz-Huerta, E.A., et al., *Arsenic contamination in irrigation water, agricultural soil and maize crop from an abandoned smelter site in Matehuala, Mexico.* Journal of hazardous Materials, 2017. **339**: p. 330-339.

223. Daus, B., et al., *Arsenic speciation in plant samples from the Iron Quadrangle, Minas Gerais, Brazil.* Microchimica Acta, 2005. **151**: p. 175-180.

224. Menezes, M., et al., *Iron Quadrangle, Brazil: Elemental concentration determined by k 0-instrumental neutron activation analysis: Part I: Soil samples.* Journal of radioanalytical and nuclear chemistry, 2006. **270**(1): p. 111-116.

225. Lopes, L.R., *Influência de fatores biológicos na mobilidade de Arsênio presente em solos da cidade de Ouro Preto–estudos “in vitro”.* 2014.

226. Santos, A.C., *Biodisponibilidade relativa e bioacessibilidade de elementos potencialmente tóxicos em solo do Quadrilátero Ferrífero.* 2019.

227. Nogueira, T.A.R., et al., *Background concentrations and quality reference values for some potentially toxic elements in soils of São Paulo State, Brazil.* Journal of Environmental Management, 2018. **221**: p. 10-19.

228. Mng'ong'o, M., et al., *Assessment of arsenic status and distribution in Usangu agro-ecosystem-Tanzania.* Journal of Environmental Management, 2021. **294**: p. 113012.

229. Adomako, E.E., C. Deacon, and A.A. Meharg, *Variations in Concentrations of Arsenic and Other Potentially Toxic Elements in Mine and Paddy Soils and Irrigation Waters from Southern Ghana.* Water Quality, Exposure and Health, 2010. **2**(2): p. 115-124.

230. Obiri-Nyarko, F., et al., *Assessment of heavy metal contamination in soils at the Kpone landfill site, Ghana: Implication for ecological and health risk assessment.* Chemosphere, 2021. **282**: p. 131007.

231. Kootbodien, T., et al., *Heavy metal contamination in a school vegetable garden in Johannesburg.* South African Medical Journal, 2012. **102**(4): p. 226-227.

232. Ramudzuli, M.R. and A.C. Horn, *Arsenic residues in soil at cattle dip tanks in the Vhembe district, Limpopo Province, South Africa.* south African Journal of science, 2014. **110**(7-8): p. 1-7.

233. Mathee, A., et al., *Concentrations of arsenic and lead in residential garden soil from four Johannesburg neighborhoods.* Environmental research, 2018. **167**: p. 524-527.

234. Kapwata, T., et al., *Relations between personal exposure to elevated concentrations of arsenic in water and soil and blood arsenic levels amongst people living in rural areas in Limpopo, South Africa.* Environmental Science and Pollution Research, 2023. **30**(24): p. 65204-65216.

235. Díaz, S.L., et al., *Control factors of the spatial distribution of arsenic and other associated elements in loess soils and waters of the southern Pampa (Argentina).* CATENA, 2016. **140**: p. 205-216.

236. Pinter, I.F., et al., *Arsenic and trace elements in soil, water, grapevine and onion in Jáchal, Argentina.* Science of the total environment, 2018. **615**: p. 1485-1498.

237. Adamu, C.I. and T.N. Nganje, *Heavy metal contamination of surface soil in relationship to land use patterns: A case study of Benue State, Nigeria.* Materials Sciences and Applications, 2010. **1**(03): p. 127.

238. Odukoya, A.M., *Contamination assessment of toxic elements in the soil within and around two dumpsites in Lagos, Nigeria.* Ife journal of science, 2015. **17**(2): p. 351-361.

239. Kayode, O.T., A. Aizebeokhai, and A. Odukoya, *Geophysical and contamination assessment of soil spatial variability for sustainable precision agriculture in Omu-Aran farm, Northcentral Nigeria.* Heliyon, 2022. **8**(2).

240. Gorbunov, A.V., et al., *Heavy and Toxic Metals in Staple Foodstuffs and Agriproduct from Contaminated Soils.* Journal of Environmental Science and Health, Part B, 2003. **38**(2): p. 181-192.

241. Rashed, M.N., *Monitoring of contaminated toxic and heavy metals, from mine tailings through age accumulation, in soil and some wild plants at Southeast Egypt.* Journal of Hazardous Materials, 2010. **178**(1): p. 739-746.

242. Badawy, W., et al., *Assessment of industrial contamination of agricultural soil adjacent to Sadat City, Egypt.* Ecological Chemistry and Engineering S, 2016. **23**(2): p. 297-310.

243. Shaheen, S.M., et al., *Arsenic, chromium, molybdenum, and selenium: Geochemical fractions and potential mobilization in riverine soil profiles originating from Germany and Egypt.* Chemosphere, 2017. **180**: p. 553-563.

244. Salman, S.A., et al., *Soil characterization and heavy metal pollution assessment in Orabi farms, El Obour, Egypt.* Bulletin of the National Research Centre, 2019. **43**(1): p. 42.

245. Shaheen, S.M., et al., *Soil contamination by potentially toxic elements and the associated human health risk in geo-and anthropogenic contaminated soils: A case study from the temperate region (Germany) and the arid region (Egypt).* Environmental Pollution, 2020. **262**: p. 114312.

246. Shetaya, W.H., et al., *Soil and plant contamination by potentially toxic and emerging elements and the associated human health risk in some Egyptian environments.* Environmental Geochemistry and Health, 2021: p. 1-21.

247. Ferreira da Silva, E., et al., *Hazard assessment on arsenic and lead in soils of Castromil gold mining area, Portugal.* Applied Geochemistry, 2004. **19**(6): p. 887-898.

248. Pratas, J., et al., *Plants growing in abandoned mines of Portugal are useful for biogeochemical exploration of arsenic, antimony, tungsten and mine reclamation.* Journal of Geochemical Exploration, 2005. **85**(3): p. 99-107.

249. Oliveira, A. and M.E. Pampulha, *Effects of long-term heavy metal contamination on soil microbial characteristics.* Journal of bioscience and bioengineering, 2006. **102**(3): p. 157-161.

250. Marques, A.P.G.C., et al., *Arsenic, lead and nickel accumulation in Rubus ulmifolius growing in contaminated soil in Portugal.* Journal of Hazardous Materials, 2009. **165**(1): p. 174-179.

251. Neiva, A.M.R., et al., *Uranium and arsenic contamination in the former Mondego Sul uranium mine area, Central Portugal.* Journal of Geochemical Exploration, 2016. **162**: p. 1-15.

252. Candeias, C., et al., *Heavy metal pollution in mine–soil–plant system in S. Francisco de Assis – Panasqueira mine (Portugal).* Applied Geochemistry, 2014. **44**: p. 12-26.

253. Neiva, A., et al., *Uranium and arsenic contamination in the former Mondego Sul uranium mine area, Central Portugal.* Journal of Geochemical Exploration, 2016. **162**: p. 1-15.

254. Antunes, I.M.H.R., et al., *Potential toxic elements in stream sediments, soils and waters in an abandoned radium mine (central Portugal).* Environmental Geochemistry and Health, 2018. **40**(1): p. 521-542.

255. Taggart, M.A., et al., *The distribution of arsenic in soils affected by the Aznalcóllar mine spill, SW Spain.* Science of The Total Environment, 2004. **323**(1): p. 137-152.

256. Díez, M., et al., *Background arsenic concentrations in Southeastern Spanish soils.* Science of The Total Environment, 2007. **378**(1): p. 5-12.

257. Anawar, H.M., et al., *Exposure and bioavailability of arsenic in contaminated soils from the La Parrilla mine, Spain.* Environmental Geology, 2006. **50**(2): p. 170-179.

258. Moreno-Jiménez, E., et al., *The fate of arsenic in soils adjacent to an old mine site (Bustarviejo, Spain): mobility and transfer to native flora.* Journal of Soils and Sediments, 2010. **10**(2): p. 301-312.

259. Moreno-Jiménez, E., et al., *Using Mediterranean shrubs for the phytoremediation of a soil impacted by pyritic wastes in Southern Spain: A field experiment.* Journal of Environmental Management, 2011. **92**(6): p. 1584-1590.

260. Oyarzun, R., et al., *Environmental assessment of the arsenic-rich, Rodalquilar gold–(copper–lead–zinc) mining district, SE Spain: data from soils and vegetation.* Environmental Geology, 2009. **58**(4): p. 761-777.

261. García-Sánchez, A., P. Alonso-Rojo, and F. Santos-Francés, *Distribution and mobility of arsenic in soils of a mining area (Western Spain).* Science of The Total Environment, 2010. **408**(19): p. 4194-4201.

262. Ramos-Miras, J.J., et al., *Influence of parent material and soil use on arsenic forms in soils: A case study in the Amblés Valley (Castilla-León, Spain).* Journal of Geochemical Exploration, 2014. **147**: p. 260-267.

263. Signes-Pastor, A.J., et al., *Geographical variation in inorganic arsenic in paddy field samples and commercial rice from the Iberian Peninsula.* Food Chemistry, 2016. **202**: p. 356-363.

264. Mesa, V., et al., *Use of Endophytic and Rhizosphere Bacteria To Improve Phytoremediation of Arsenic-Contaminated Industrial Soils by Autochthonous Betula celtiberica.* Applied and Environmental Microbiology, 2017. **83**(8): p. e03411-16.

265. Keegan, T.J., et al., *Dispersion of As and selected heavy metals around a coal-burning power station in central Slovakia.* Science of The Total Environment, 2006. **358**(1): p. 61-71.

266. Jurkovič, L.u., et al., *Arsenic Concentrations in Soils Impacted by Dam Failure of Coal-Ash Pond in Zemianske Kostolany, Slovakia.* Bulletin of Environmental Contamination and Toxicology, 2011. **86**(4): p. 433-437.

267. Jurkovič, Ľ., et al., *Natural attenuation of antimony and arsenic in soils at the abandoned Sb-deposit Poproč, Slovakia.* Environmental Earth Sciences, 2019. **78**(24): p. 672.

268. Fazekašová, D. and J. Fazekaš, *Soil Quality and Heavy Metal Pollution Assessment of Iron Ore Mines in Nizna Slana (Slovakia).* Sustainability, 2020. **12**(6): p. 2549.

269. Kobza, J., *Arsenic in Agricultural Soils of Slovakia.* Polish Journal of Soil Science, 2021(1).

270. Rachwał, M., et al., *Application of magnetic susceptibility in assessment of heavy metal contamination of Saxonian soil (Germany) caused by industrial dust deposition.* Geoderma, 2017. **295**: p. 10-21.

271. Kalbitz, K. and R. Wennrich, *Mobilization of heavy metals and arsenic in polluted wetland soils and its dependence on dissolved organic matter.* Science of The Total Environment, 1998. **209**(1): p. 27-39.

272. Meyer, I., J. Heinrich, and U. Lippold, *Factors affecting lead, cadmium, and arsenic levels in house dust in a smelter town in eastern Germany.* Environmental Research, 1999. **81**(1): p. 32-44.

273. Krüger, F., et al., *Flood Induced Heavy Metal and Arsenic Contamination of Elbe River Floodplain Soils.* Acta hydrochimica et hydrobiologica, 2005. **33**(5): p. 455-465.

274. Tarvainen, T., et al., *Arsenic in agro-ecosystems under anthropogenic pressure in Germany and France compared to a geogenic As region in Finland.* Journal of Geochemical Exploration, 2020. **217**: p. 106606.

275. Frohne, T., J. Rinklebe, and R.A. Diaz-Bone, *Contamination of Floodplain Soils along the Wupper River, Germany, with As, Co, Cu, Ni, Sb, and Zn and the Impact of Pre-definite Redox Variations on the Mobility of These Elements.* Soil and Sediment Contamination: An International Journal, 2014. **23**(7): p. 779-799.

276. Sterckeman, T., et al., *Assessment of the Contamination of Cultivated Soils by Eighteen Trace Elements Around Smelters in the North of France.* Water, Air, and Soil Pollution, 2002. **135**(1): p. 173-194.

277. Fillol, C., et al., *Does arsenic in soil contribute to arsenic urinary concentrations in a French population living in a naturally arsenic contaminated area?* Science of The Total Environment, 2010. **408**(23): p. 6011-6016.

278. Jean-Soro, L., et al., *Origin of trace elements in an urban garden in Nantes, France.* Journal of Soils and Sediments, 2015. **15**(8): p. 1802-1812.

279. Rabier, J., et al., *Heavy Metal and Arsenic Resistance of the Halophyte Atriplex halimus L. Along a Gradient of Contamination in a French Mediterranean Spray Zone.* Water, Air, & Soil Pollution, 2014. **225**(7): p. 1993.

280. Marchant, B.P., N.P.A. Saby, and D. Arrouays, *A survey of topsoil arsenic and mercury concentrations across France.* Chemosphere, 2017. **181**: p. 635-644.

281. Rehman, I.u., et al., *Evaluation of arsenic contamination and potential risks assessment through water, soil and rice consumption.* Environmental Technology & Innovation, 2020. **20**: p. 101155.

282. Delplace, G., et al., *Pedo-geochemical background and sediment contamination of metal(loid)s in the old mining-district of Salsigne (Orbiel valley, France).* Chemosphere, 2022. **287**: p. 132111.

283. Schultz, E., et al., *Extractability of metals and ecotoxicity of soils from two old wood impregnation sites in Finland.* Science of The Total Environment, 2004. **326**(1): p. 71-84.

284. Salonen, V.-P. and K. Korkka-Niemi, *Influence of parent sediments on the concentration of heavy metals in urban and suburban soils in Turku, Finland.* Applied Geochemistry, 2007. **22**(5): p. 906-918.

285. Jarva, J., T. Tarvainen, and J. Reinikainen, *Application of arsenic baselines in the assessment of soil contamination in Finland.* Environmental Geochemistry and Health, 2008. **30**(6): p. 613-621.

286. Buttafuoco, G., et al., *Spatial variability and trigger values of arsenic in the surface urban soils of the cities of Tampere and Lahti, Finland.* Environmental Earth Sciences, 2016. **75**(10): p. 896.

287. Kilpi-Koski, J., et al., *An uptake and elimination kinetics approach to assess the bioavailability of chromium, copper, and arsenic to earthworms (Eisenia andrei) in contaminated field soils.* Environmental Science and Pollution Research, 2019. **26**(15): p. 15095-15104.

288. Karimi, N., et al., *Analysis of Arsenic in Soil and Vegetation of a Contaminated Area in Zarshuran, Iran.* International Journal of Phytoremediation, 2009. **12**(2): p. 159-173.

289. Sharifi, Z., A.A.S. Sinegani, and S. Shariati, *Potential of Indigenous Plant Species for the Phytoremediation of Arsenic Contaminated Land in Kurdistan (Iran).* Soil and Sediment Contamination: An International Journal, 2012. **21**(5): p. 557-573.

290. Tavakoli, H., et al., *Human health risk assessment of arsenic downstream of a steel plant in Isfahan, Iran: a case study.* International Journal of Environmental Science and Technology, 2020. **17**(1): p. 81-92.

291. Karimi, N., S.M. Ghaderian, and H. Schat, *Arsenic in soil and vegetation of a contaminated area.* International Journal of Environmental Science and Technology, 2013. **10**(4): p. 743-752.

292. Taheri, M., et al., *High soil and groundwater arsenic levels induce high body arsenic loads, health risk and potential anemia for inhabitants of northeastern Iran.* Environmental Geochemistry and Health, 2016. **38**(2): p. 469-482.

293. Ghazban, F., et al., *Evaluation of Heavy Metal Contamination of Surface Soils in Zarshouran Gold District, Northwestern Iran.* International Journal of Environmental Research, 2018. **12**(6): p. 843-860.

294. Khadem Moghadam Igdelou, N. and A. Golchin, *Risk assessment of contamination of the country's soil and water resources with arsenic.* Iranian Journal of Soil and Water Research, 2019. **50**(7): p. 1595-1617.

295. Rastegari Mehr, M., et al., *Arsenic in the rock–soil–plant system and related health risk in a magmatic–metamorphic belt, West of Iran.* Environmental Geochemistry and Health, 2020. **42**(11): p. 3659-3673.

296. Rastegari Mehr, M., et al., *Bioavailability, distribution and health risk assessment of arsenic and heavy metals (HMs) in agricultural soils of Kermanshah Province, west of Iran.* Journal of Environmental Health Science and Engineering, 2021. **19**(1): p. 107-120.

297. Nemati-Mansour, S., et al., *Environmental occurrence and health risk assessment of arsenic in Iran: a systematic review and Meta-analysis.* Human and Ecological Risk Assessment: An International Journal, 2022. **28**(5-6): p. 683-710.

298. Gong, Y., et al., *Status of arsenic accumulation in agricultural soils across China (1985–2016).* Environmental Research, 2020. **186**: p. 109525.

299. Kertulis-Tartar, G.M., et al., *Phytoremediation of an Arsenic-Contaminated Site Using Pteris vittata L.: A Two-Year Study.* International Journal of Phytoremediation, 2006. **8**(4): p. 311-322.

300. Zhang, H.H., et al., *Spatial distribution and vertical variation of arsenic in Guangdong soil profiles, China.* Environmental Pollution, 2006. **144**(2): p. 492-499.

301. Tong-Bin, C., et al., *Phytoremediation of arsenic-contaminated soil in China.* Phytoremediation: Methods and Reviews, 2007: p. 393-404.

302. Ye, W.-L., et al., *Phytoremediation of arsenic contaminated paddy soils with Pteris vittata markedly reduces arsenic uptake by rice.* Environmental Pollution, 2011. **159**(12): p. 3739-3743.

303. Shi, T., et al., *Monitoring Arsenic Contamination in Agricultural Soils with Reflectance Spectroscopy of Rice Plants.* Environmental Science & Technology, 2014. **48**(11): p. 6264-6272.

304. Mandal, A., T.J. Purakayastha, and A.K. Patra, *Phytoextraction of arsenic contaminated soil by Chinese brake fern (Pteris vittata): Effect on soil microbiological activities.* Biology and Fertility of Soils, 2014. **50**(8): p. 1247-1252.

305. Lei, M., et al., *Phytoextraction of arsenic-contaminated soil with Pteris vittata in Henan Province, China: comprehensive evaluation of remediation efficiency correcting for atmospheric depositions.* Environmental Science and Pollution Research, 2018. **25**(1): p. 124-131.

306. Xue, S., et al., *Cadmium, lead, and arsenic contamination in paddy soils of a mining area and their exposure effects on human HEPG2 and keratinocyte cell-lines.* Environmental Research, 2017. **156**: p. 23-30.

307. Jia, X., et al., *Mapping soil pollution by using drone image recognition and machine learning at an arsenic-contaminated agricultural field.* Environmental Pollution, 2021. **270**: p. 116281.

308. Patel, K.S., et al., *Arsenic contamination in water, soil, sediment and rice of central India.* Environmental Geochemistry and Health, 2005. **27**(2): p. 131-145.

309. Roychowdhury, T., et al., *Arsenic and other heavy metals in soils from an arsenic-affected area of West Bengal, India.* Chemosphere, 2002. **49**(6): p. 605-618.

310. Ghosh, A.K., P. Bhattacharyya, and R. Pal, *Effect of arsenic contamination on microbial biomass and its activities in arsenic contaminated soils of Gangetic West Bengal, India.* Environment International, 2004. **30**(4): p. 491-499.

311. Acharyya, S.K., et al., *Arsenic contamination in groundwater from parts of Ambagarh-Chowki block, Chhattisgarh, India: source and release mechanism.* Environmental Geology, 2005. **49**(1): p. 148-158.

312. Dwivedi, S., et al., *Arsenic affects mineral nutrients in grains of various Indian rice (Oryza sativa L.) genotypes grown on arsenic-contaminated soils of West Bengal.* Protoplasma, 2010. **245**(1): p. 113-124.

313. Chakraborti, D., et al., *Environmental arsenic contamination and its health effects in a historic gold mining area of the Mangalur greenstone belt of Northeastern Karnataka, India.* Journal of Hazardous Materials, 2013. **262**: p. 1048-1055.

314. Rahaman, S., et al., *Arsenic contamination: a potential hazard to the affected areas of West Bengal, India.* Environmental Geochemistry and Health, 2013. **35**(1): p. 119-132.

315. Srivastava, S. and Y.K. Sharma, *Arsenic occurrence and accumulation in soil and water of eastern districts of Uttar Pradesh, India.* Environmental Monitoring and Assessment, 2013. **185**(6): p. 4995-5002.

316. Shrivastava, A., et al., *Arsenic contamination in shallow groundwater and agricultural soil of Chakdaha block, West Bengal, India.* Frontiers in Environmental Science, 2014. **2**: p. 50.

317. Shrivastava, A., et al., *Arsenic Contamination in Soil and Sediment in India: Sources, Effects, and Remediation.* Current Pollution Reports, 2015. **1**(1): p. 35-46.

318. Gupta, K., et al., *Phyto-genotoxicity of arsenic contaminated soil from Lakhimpur Kheri, India on Vicia faba L.* Chemosphere, 2020. **241**: p. 125063.

319. Karim, M.M., Y. Komori, and M. Alam, *Subsurface Arsenic Occurrence and Depth of Contamination in Bangladesh.* 環境化学, 1997. **7**(4): p. 783-792.

320. Alam, M.B. and M.A. Sattar, *Assessment of arsenic contamination in soils and waters in some areas of Bangladesh.* Water Science and Technology, 2000. **42**(7-8): p. 185-192.

321. Meharg, A.A. and M.M. Rahman, *Arsenic Contamination of Bangladesh Paddy Field Soils:  Implications for Rice Contribution to Arsenic Consumption.* Environmental Science & Technology, 2003. **37**(2): p. 229-234.

322. Saha, G.C. and M.A. Ali, *Dynamics of arsenic in agricultural soils irrigated with arsenic contaminated groundwater in Bangladesh.* Science of The Total Environment, 2007. **379**(2): p. 180-189.

323. Hossain, M.F., *Arsenic contamination in Bangladesh—An overview.* Agriculture, Ecosystems & Environment, 2006. **113**(1): p. 1-16.

324. Spallholz, J.E., et al., *Selenium and arsenic content of agricultural soils from Bangladesh and Nepal.* Toxicological & Environmental Chemistry, 2008. **90**(2): p. 203-210.

325. Ahsan, D.A., T.A. DelValls, and J. Blasco, *Distribution of Arsenic and Trace Metals in the Floodplain Agricultural Soil of Bangladesh.* Bulletin of Environmental Contamination and Toxicology, 2009. **82**(1): p. 11-15.

326. Abdullah, S.M.A., et al., *Analysis of Arsenic Concentrations and Correlation in Water, Soil and Aurum by Neutron Activation Analysis Technique: A Case Study in Bagerhat, Bangladesh.* Bulletin of Environmental Contamination and Toxicology, 2010. **85**(3): p. 301-306.

327. Kurosawa, K., et al., *Groundwater–soil–crop relationship with respect to arsenic contamination in farming villages of Bangladesh – A preliminary study.* Environmental Pollution, 2008. **156**(2): p. 563-565.

328. Camm, G.S., et al., *Characterisation of a mining-related arsenic-contaminated site, Cornwall, UK.* Journal of Geochemical Exploration, 2004. **82**(1): p. 1-15.

329. Hartley, W., et al., *Arsenic stability and mobilization in soil at an amenity grassland overlying chemical waste (St. Helens, UK).* Environmental Pollution, 2009. **157**(3): p. 847-856.

330. Potts, P.J., M.H. Ramsey, and J. Carlisle, *Portable X-ray fluorescence in the characterisation of arsenic contamination associated with industrial buildings at a heritage arsenic works site near Redruth, Cornwall, UK.* Journal of Environmental Monitoring, 2002. **4**(6): p. 1017-1024.

331. Madejón, P. and N.W. Lepp, *Arsenic in soils and plants of woodland regenerated on an arsenic-contaminated substrate: A sustainable natural remediation?* Science of The Total Environment, 2007. **379**(2): p. 256-262.

332. Saikat, S., B. Barnes, and D. Westwood, *A review of laboratory results for bioaccessibility values of arsenic, lead and nickel in contaminated UK soils.* Journal of Environmental Science and Health, Part A, 2007. **42**(9): p. 1213-1221.

333. Šlejkovec, Z., et al., *Speciation analysis to unravel the soil-to-plant transfer in highly arsenic-contaminated areas in Cornwall (UK).* International Journal of Environmental Analytical Chemistry, 2010. **90**(10): p. 784-796.

334. Hartley, T.N., et al., *Historical arsenic contamination of soil due to long-term phosphate fertiliser applications.* Environmental Pollution, 2013. **180**: p. 259-264.

335. Middleton, D.R.S., et al., *Arsenic in residential soil and household dust in Cornwall, south west England: potential human exposure and the influence of historical mining.* Environmental Science: Processes & Impacts, 2017. **19**(4): p. 517-527.

336. Ostachowicz, J., et al., *Application of EDXRF in a survey of concentrations of lead, zinc and arsenic in soil from selected areas in Krakow, Poland.* X-Ray Spectrometry, 1995. **24**(2): p. 81-83.

337. Loska, K., et al., *Assessment of arsenic enrichment of cultivated soils in Southern Poland.* Polish Journal of Environmental Studies, 2003. **12**(2): p. 187-192.

338. Loska, K., D. Wiechuła, and I. Korus, *Metal contamination of farming soils affected by industry.* Environment International, 2004. **30**(2): p. 159-165.

339. Staszewski, T., et al., *Soil and plants contamination with selected heavy metals in the area of a railway junction.* Archives of Environmental Protection, 2015. **41**(1): p. 35--42.

340. Medyńska, A., *Arsenic contamination and mobility in forest soils under impact of copper smelter in SW Poland*, in *Arsenic in Geosphere and Human Diseases; Arsenic 2010*. 2010, CRC Press. p. 164-165.

341. Andrzej, et al., *Land-use impact on selected forms of arsenic and phosphorus in soils of different functions.* International Agrophysics, 2017. **31**(4).

342. Dradrach, A., et al., *Accumulation of Arsenic by Plants Growing in the Sites Strongly Contaminated by Historical Mining in the Sudetes Region of Poland.* International Journal of Environmental Research and Public Health, 2020. **17**(9): p. 3342.

343. Chen, M., et al., *Arsenic Background Concentrations in Florida, U.S.A. Surface Soils: Determination and Interpretation.* Environmental Forensics, 2001. **2**(2): p. 117-126.

344. Peryea, F.J. and T.L. Creger, *Vertical distribution of lead and arsenic in soils contaminated with lead arsenate pesticide residues.* Water, Air, and Soil Pollution, 1994. **78**(3): p. 297-306.

345. Francesconi, K., et al., *Arsenic species in an arsenic hyperaccumulating fern, Pityrogramma calomelanos: a potential phytoremediator of arsenic-contaminated soils.* Science of The Total Environment, 2002. **284**(1): p. 27-35.

346. Chirenje, T., et al., *Arsenic Distribution in Florida Urban Soils.* Journal of Environmental Quality, 2003. **32**(1): p. 109-119.

347. Yang, L. and R.J. Donahoe, *The form, distribution and mobility of arsenic in soils contaminated by arsenic trioxide, at sites in southeast USA.* Applied Geochemistry, 2007. **22**(2): p. 320-341.

348. Kim, E.J., J.-C. Yoo, and K. Baek, *Arsenic speciation and bioaccessibility in arsenic-contaminated soils: Sequential extraction and mineralogical investigation.* Environmental Pollution, 2014. **186**: p. 29-35.

349. Aide, M., D. Beighley, and D. Dunn, *Soil Profile Arsenic Concentration Distributions in Missouri Soils Having Cambic and Argillic Soil Horizons.* Soil and Sediment Contamination: An International Journal, 2014. **23**(3): p. 313-327.

350. Codling, E.E., R.L. Chaney, and C.E. Green, *Accumulation of Lead and Arsenic by Carrots Grown on Lead-Arsenate Contaminated Orchard Soils.* Journal of Plant Nutrition, 2015. **38**(4): p. 509-525.

351. Neupane, G. and R.J. Donahoe, *Calcium–phosphate treatment of contaminated soil for arsenic immobilization.* Applied Geochemistry, 2013. **28**: p. 145-154.

352. Nezat, C.A., S.A. Hatch, and T. Uecker, *Heavy metal content in urban residential and park soils: A case study in Spokane, Washington, USA.* Applied Geochemistry, 2017. **78**: p. 186-193.

353. Flynn, H.C., et al., *Assessment of bioavailable arsenic and copper in soils and sediments from the Antofagasta region of northern Chile.* Science of The Total Environment, 2002. **286**(1): p. 51-59.

354. De Gregori, I., et al., *Monitoring of copper, arsenic and antimony levels in agricultural soils impacted and non-impacted by mining activities, from three regions in Chile.* Journal of Environmental Monitoring, 2003. **5**(2): p. 287-295.

355. Díaz, O., et al., *Total and Bioavailable Arsenic Concentration in Arid Soils and its Uptake by Native Plants from the Pre-Andean Zones in Chile.* Bulletin of Environmental Contamination and Toxicology, 2011. **86**(6): p. 666-669.

356. Ahumada, I., et al., *Extractability of Arsenic, Copper, and Lead in Soils of a Mining and Agricultural Zone in Central Chile.* Communications in Soil Science and Plant Analysis, 2004. **35**(11-12): p. 1615-1634.

357. Tarvainen, T., A. Reyes, and S. Sapon, *Acceptable soil baseline levels in Taltal, Chile, and in Tampere, Finland.* Applied Geochemistry, 2020. **123**: p. 104813.

358. Bustos, V., et al., *Thresholds of arsenic toxicity to Eisenia fetida in field-collected agricultural soils exposed to copper mining activities in Chile.* Ecotoxicology and Environmental Safety, 2015. **122**: p. 448-454.

359. Tapia-Gatica, J., et al., *Advanced determination of the spatial gradient of human health risk and ecological risk from exposure to As, Cu, Pb, and Zn in soils near the Ventanas Industrial Complex (Puchuncaví, Chile).* Environmental Pollution, 2020. **258**: p. 113488.

360. Madrid, E., et al., *Arsenic concentration in topsoil of central Chile is associated with aberrant methylation of P53 gene in human blood cells: a cross-sectional study.* Environmental Science and Pollution Research, 2022. **29**(32): p. 48250-48259.

361. Alam, M.A., et al., *An appraisal of the principal concerns and controlling factors for Arsenic contamination in Chile.* Scientific Reports, 2023. **13**(1): p. 11168.

362. Baroni, F., et al., *Arsenic in soil and vegetation of contaminated areas in southern Tuscany (Italy).* Journal of Geochemical Exploration, 2004. **81**(1): p. 1-14.

363. Gál, J., A.S. Hursthouse, and S.J. Cuthbert, *Chemical availability of arsenic and antimony in industrial soils.* Environmental Chemistry Letters, 2006. **3**(4): p. 149-153.

364. Ungaro, F., et al., *Arsenic concentration in the soils of the Brenta Plain (Northern Italy): Mapping the probability of exceeding contamination thresholds.* Journal of Geochemical Exploration, 2008. **96**(2): p. 117-131.

365. Cavalca, L., et al., *Rhizobacterial communities associated with spontaneous plant species in long-term arsenic contaminated soils.* World Journal of Microbiology and Biotechnology, 2015. **31**(5): p. 735-746.

366. Cattani, I., G.M. Beone, and C. Gonnelli, *Influence of Rhizophagus irregularis inoculation and phosphorus application on growth and arsenic accumulation in maize (Zea mays L.) cultivated on an arsenic-contaminated soil.* Environmental Science and Pollution Research, 2015. **22**(9): p. 6570-6577.

367. Vamerali, T., et al., *Phytoremediation trials on metal- and arsenic-contaminated pyrite wastes (Torviscosa, Italy).* Environmental Pollution, 2009. **157**(3): p. 887-894.

368. Rimondi, V., et al., *Bioaccessible arsenic in soil of thermal areas of Viterbo, Central Italy: implications for human health risk.* Environmental Geochemistry and Health, 2022. **44**(2): p. 465-485.

369. Tremlová, J., et al., *A profile of arsenic species in different vegetables growing in arsenic-contaminated soils.* Archives of Agronomy and Soil Science, 2017. **63**(7): p. 918-927.

370. Jung, M.C., I. Thornton, and H.-T. Chon, *Arsenic, Sb and Bi contamination of soils, plants, waters and sediments in the vicinity of the Dalsung Cu–W mine in Korea.* Science of The Total Environment, 2002. **295**(1): p. 81-89.

371. Ko, I., et al., *Arsenic contamination of soils and sediments from tailings in the vicinity of Myungbong Au mine, Korea.* Chemical Speciation & Bioavailability, 2003. **15**(3): p. 67-74.

372. Jang, M., et al., *Remediation of arsenic-contaminated soils and washing effluents.* Chemosphere, 2005. **60**(3): p. 344-354.

373. Ko, M.-S., et al., *Stabilization of the As-contaminated soil from the metal mining areas in Korea.* Environmental Geochemistry and Health, 2012. **34**(1): p. 143-149.

374. Shagol, C.C., et al., *Arsenic-tolerant plant-growth-promoting bacteria isolated from arsenic-polluted soils in South Korea.* Environmental Science and Pollution Research, 2014. **21**(15): p. 9356-9365.

375. Kwon, J.C., Z.D. Nejad, and M.C. Jung, *Arsenic and heavy metals in paddy soil and polished rice contaminated by mining activities in Korea.* CATENA, 2017. **148**: p. 92-100.

376. Lee, P.-K., et al., *Source identification of arsenic contamination in agricultural soils surrounding a closed Cu smelter, South Korea.* Chemosphere, 2019. **217**: p. 183-194.

377. Phan, K., et al., *Arsenic contamination in the food chain and its risk assessment of populations residing in the Mekong River basin of Cambodia.* Journal of Hazardous Materials, 2013. **262**: p. 1064-1071.

378. Seyfferth, A.L., et al., *Arsenic Concentrations in Paddy Soil and Rice and Health Implications for Major Rice-Growing Regions of Cambodia.* Environmental Science & Technology, 2014. **48**(9): p. 4699-4706.

379. Hamzah, A., et al., *Determination of total arsenic in soil and arsenic-resistant bacteria from selected ground water in Kandal Province, Cambodia.* Journal of Radioanalytical and Nuclear Chemistry, 2013. **297**(2): p. 291-296.

380. Murphy, T., et al., *Effects of Arsenic, Iron and Fertilizers in Soil on Rice in Cambodia.* Journal of Health and Pollution, 2018. **8**(19).

381. Arao, T., et al., *Arsenic Contamination in Soils and Crops in Japan and Various Countermeasures (Symposium 3.5. 1 Heavy Metal Contaminated Soils,< Special Issue> International Symposium: Soil Degradation Control, Remediation, and Reclamation, Tokyo Metropolitan University Symposium Series No. 2, 2010).* Pedologist, 2011. **54**(3): p. 202-213.

382. Alam, M.G.M., S. Tokunaga, and T. Maekawa, *Extraction of arsenic in a synthetic arsenic-contaminated soil using phosphate.* Chemosphere, 2001. **43**(8): p. 1035-1041.

383. Itabashi, T., et al., *Speciation and Fractionation of Soil Arsenic from Natural and Anthropogenic Sources: Chemical Extraction, Scanning Electron Microscopy, and Micro-XRF/XAFS Investigation.* Environmental Science & Technology, 2019. **53**(24): p. 14186-14193.

384. Kohda, Y.H.-T., et al., *Arsenic uptake by Pteris vittata in a subarctic arsenic-contaminated agricultural field in Japan: An 8-year study.* Science of The Total Environment, 2022. **831**: p. 154830.

385. Rahman, S., et al., *Biodegradable Chelator-Assisted Washing and Stabilization of Arsenic-Contaminated Excavated Soils.* Water, Air, & Soil Pollution, 2022. **233**(6): p. 213.

386. Phuong, N.M., et al., *Arsenic contents and physicochemical properties of agricultural soils from the Red River Delta, Vietnam.* Soil Science and Plant Nutrition, 2008. **54**(6): p. 846-855.

387. Chu Ngoc, K., et al., *Arsenic and Heavy Metal Concentrations in Agricultural Soils Around Tin and Tungsten Mines in the Dai Tu district, N. Vietnam.* Water, Air, and Soil Pollution, 2009. **197**(1): p. 75-89.

388. Dung, T.T.T., et al., *Leachability of arsenic and heavy metals from blasted copper slag and contamination of marine sediment and soil in Ninh Hoa district, south central of Vietnam.* Applied Geochemistry, 2014. **44**: p. 80-92.

389. Bui, A.T.K., et al., *Accumulation and potential health risks of cadmium, lead and arsenic in vegetables grown near mining sites in Northern Vietnam.* Environmental Monitoring and Assessment, 2016. **188**(9): p. 525.

390. Phenrat, T., et al., *Arsenic residue in residential area after cleanup of pesticide illegal dumping sources in Thanh Hoa province, Central Vietnam.* Environmental Forensics, 2018. **19**(1): p. 66-78.

391. Ha, N.T.H., et al., *Uptake of arsenic and heavy metals by native plants growing near Nui Phao multi-metal mine, northern Vietnam.* Applied Geochemistry, 2019. **108**: p. 104368.

392. Ko, M.-S., et al., *Assessment and source identification of As and Cd contamination in soil and plants in the vicinity of the Nui Phao Mine, Vietnam.* Environmental Geochemistry and Health, 2020. **42**(12): p. 4193-4201.

393. Zarcinas, B.A., et al., *Heavy metals in soils and crops in Southeast Asia 2. Thailand.* Environmental Geochemistry and Health, 2004. **26**(3): p. 359-371.

394. Visoottiviseth, P., K. Francesconi, and W. Sridokchan, *The potential of Thai indigenous plant species for the phytoremediation of arsenic contaminated land.* Environmental pollution, 2002. **118**(3): p. 453-461.

395. Jankong, P., P. Visoottiviseth, and S. Khokiattiwong, *Enhanced phytoremediation of arsenic contaminated land.* Chemosphere, 2007. **68**(10): p. 1906-1912.

396. Chintakovid, W., et al., *Potential of the hybrid marigolds for arsenic phytoremediation and income generation of remediators in Ron Phibun District, Thailand.* Chemosphere, 2008. **70**(8): p. 1532-1537.

397. Jareonmit, P., K. Sajjaphan, and M.J. Sadowsky, *Structure and diversity of arsenic-resistant bacteria in an old tin mine area of Thailand.* J Microbiol Biotechnol, 2010. **20**(1): p. 169-178.

398. Sowana, A., et al., *Influence of coastal land use on soil heavy-metal contamination in Pattani Bay, Thailand.* Journal of Coastal Research, 2011. **27**(2): p. 252-262.

399. Sukreeyapongse, O., C. Tepwituksakit, and N. Notesiri. *Arsenic in soil, water and plant at contaminated sites and in agricultural soil of Thailand*. in *Macro Symposium 2009: Challenges for Agro-Environmental Research in Monsoon Asia*. 2009.

400. Tiankao, W. and S. Chotpantarat, *Risk assessment of arsenic from contaminated soils to shallow groundwater in Ong Phra Sub-District, Suphan Buri Province, Thailand.* Journal of Hydrology: Regional Studies, 2018. **19**: p. 80-96.

401. Baubekova, A., et al., *Evaluation of environmental contamination by toxic trace elements in Kazakhstan based on reviews of available scientific data.* Environmental Science and Pollution Research, 2021. **28**(32): p. 43315-43328.

402. Bou Kheir, R., et al., *A Comparative GIS tree‑pollution analysis between arsenic, chromium, mercury, and uranium contents in soils of urban and industrial regions in Qatar.* Euro-Mediterranean Journal for Environmental Integration, 2019. **4**(1): p. 10.

403. Alsafran, M., et al., *Ecological and Health Risks Assessment of Potentially Toxic Metals and Metalloids Contaminants: A Case Study of Agricultural Soils in Qatar.* Toxics, 2021. **9**(2): p. 35.

404. Wijesekara, G. and B. Marambe, *Arsenic in the environment–an overview on global and Sri Lankan context.* Ann. Sri Lanka Dep. Agric, 2011. **13**: p. 229-243.

405. Jayawardana, D.T., H.M.T.G.A. Pitawala, and H. Ishiga, *Assessment of soil geochemistry around some selected agricultural sites of Sri Lanka.* Environmental Earth Sciences, 2014. **71**(9): p. 4097-4106.

406. Prabagar, S., et al., *Accumulation of heavy metals in grape fruit, leaves, soil and water: A study of influential factors and evaluating ecological risks in Jaffna, Sri Lanka.* Environmental and Sustainability Indicators, 2021. **12**: p. 100147.

407. Pranoto, B.S.M. and W. Budianta, *Phytoremediation of heavy metals contaminated soil in artisanal gold mining at Selogiri, Wonogiri District, Central Java, Indonesia.* Journal of Applied Geology, 2020. **5**(2): p. 64-72.

408. Gafur, N.A., et al., *Environmental Survey of the Distribution and Metal Contents of Pteris vittata in Arsenic&ndash;Lead&ndash;Mercury-Contaminated Gold Mining Areas along the Bone River in Gorontalo Province, Indonesia.* International Journal of Environmental Research and Public Health, 2022. **19**(1): p. 530.

409. Ghrefat, H., et al., *Pollution assessment of arsenic and other selected elements in the groundwater and soil of the Gulf of Aqaba, Saudi Arabia.* Environmental Earth Sciences, 2016. **75**(3): p. 229.

410. Alarifi, S.S., et al., *Contamination and Environmental Risk Assessment of Potentially Toxic Elements in Soils of Palm Farms in Northwest Riyadh, Saudi Arabia.* Sustainability, 2022. **14**(22): p. 15402.

411. Alharbi, T. and A.S. El-Sorogy, *Risk Assessment of Potentially Toxic Elements in Agricultural Soils of Al-Ahsa Oasis, Saudi Arabia.* Sustainability, 2023. **15**(1): p. 659.

412. AlKhader, A., *The impact of phosphorus fertilizers on heavy metals content of soils and vegetables grown on selected farms in Jordan.* Agrotechnology, 2015. **5**(1): p. 137.

413. Ong, G.H., et al., *An investigation of arsenic contamination in Peninsular Malaysia based on Centella asiatica and soil samples.* Environmental Monitoring and Assessment, 2013. **185**(4): p. 3243-3254.

414. Atiaga, O., et al., *Toxic Elements in Soil and Rice in Ecuador.* Agronomy, 2021. **11**(8): p. 1594.

415. Jimenez, P.A.J., et al., *Assessing and Understanding Arsenic Contamination in Agricultural Soils and Lake Sediments from Papallacta Rural Parish, Northeastern Ecuador, via Ecotoxicology Factors, for Environmental Embasement.* Sustainability, 2023. **15**(5): p. 3951.

416. Bech, J., et al., *Arsenic and heavy metal contamination of soil and vegetation around a copper mine in Northern Peru.* Science of The Total Environment, 1997. **203**(1): p. 83-91.

417. Forslund, J., et al., *Does remediation save lives? — On the cost of cleaning up arsenic-contaminated sites in Sweden.* Science of The Total Environment, 2010. **408**(16): p. 3085-3091.

418. Pfeifer, H.-R., et al., *Distribution and behavior of arsenic in soils and waters in the vicinity of the former gold-arsenic mine of Salanfe, Western Switzerland.* Journal of Geochemical Exploration, 2007. **93**(3): p. 121-134.

419. Helgesen, H. and E.H. Larsen, *Bioavailability and speciation of arsenic in carrots grown in contaminated soil.* Analyst, 1998. **123**(5): p. 791-796.

420. Cordos, E.A., et al., *Distribution study of inorganic arsenic (III) and (V) species in soil and their mobility in the area of Baia-Mare, Romania.* Chemical Speciation & Bioavailability, 2006. **18**(1): p. 11-25.

421. Senila, M., et al., *Arsenic and antimony content in soil and plants from Baia Mare area, Romania.* Am. J. Environ. Sci, 2010. **6**: p. 33-40.

422. Ilieva, D.M., et al., *Application of chemical and biological tests for estimation of current state of a tailing dump and surrounding soil from the region of Tarniţa, Suceava, Romania.* Environmental Science and Pollution Research, 2020. **27**(2): p. 1386-1396.

423. Crnković, D., M. Ristić, and D. Antonović, *Distribution of Heavy Metals and Arsenic in Soils of Belgrade (Serbia and Montenegro).* Soil and Sediment Contamination: An International Journal, 2006. **15**(6): p. 581-589.

424. Antonijević, M., et al., *Metal concentrations in the soils and native plants surrounding the old flotation tailings pond of the Copper Mining and Smelting Complex Bor (Serbia).* Journal of Environmental Monitoring, 2012. **14**(3): p. 866-877.

425. Spahić, M.P., et al., *Assessment of contamination, environmental risk, and origin of heavy metals in soils surrounding industrial facilities in Vojvodina, Serbia.* Environmental Monitoring and Assessment, 2018. **190**(4): p. 208.

426. Barać, N., et al., *Arsenic in Agricultural Soils of a Historically Mined and Industrial Region of Southern Serbia and Northern Kosovo: Bioavailability and Uptake by Plants Species Zea mays L. and Solanum tuberosum L.* Soil and Sediment Contamination: An International Journal, 2015. **24**(6): p. 656-674.

427. Kireycheva, L.V., A.V. Ilinskiy, and L.I. Moskovkina, *Use of the combined prolonged ameliorant for detoxification of alluvial sod loam soil contaminated with Arsenic in Russia.* American Scientific Journal, 2016(7): p. 4-10.

428. Mlangeni, A.T., et al., *Evaluation of Metal(loids) Concentrations in Soils of Selected Rice Paddy Fields in Malawi.* Agronomy, 2022. **12**(10): p. 2349.

429. Mussa, C., et al., *Occurrence and ecological risk assessment of heavy metals in agricultural soils of Lake Chilwa catchment in Malawi, Southern Africa.* SN Applied Sciences, 2020. **2**(11): p. 1910.

430. Nyanza, E.C., et al., *Spatial Distribution of Mercury and Arsenic Levels in Water, Soil and Cassava Plants in a Community with Long History of Gold Mining in Tanzania.* Bulletin of Environmental Contamination and Toxicology, 2014. **93**(6): p. 716-721.

431. Iavazzo, P., et al., *Impact of past mining activity on the quality of water and soil in the High Moulouya Valley (Morocco).* Water, Air, & Soil Pollution, 2012. **223**: p. 573-589.

432. El Amari, K., et al., *Impact of mine tailings on surrounding soils and ground water: Case of Kettara old mine, Morocco.* Journal of African Earth Sciences, 2014. **100**: p. 437-449.

433. Sofia, F., et al., *Behavior of As, Cd, Co, Cr, Cu, Pb, Ni, and Zn at the soil/plant interface around an uncontrolled landfill (Casablanca, Morocco).* Remediation Journal, 2018. **28**(4): p. 65-72.

434. Ech-Charef, A., et al., *Soil heavy metal contamination in the vicinity of the abandoned Zeïda mine in the Upper Moulouya Basin, Morocco. Implications for airborne dust pollution under semi-arid climatic conditions.* Journal of African Earth Sciences, 2023. **198**: p. 104812.

435. Nassiri, O., et al., *Environmental and Geochemical Characteristics of Heavy Metals in Soils Around the Former Mining Area of Zeïda (High Moulouya, Morocco).* Water, Air, & Soil Pollution, 2023. **234**(2): p. 110.

436. Hinwood, A.L., et al., *Exposure to Inorganic Arsenic in Soil Increases Urinary Inorganic Arsenic Concentrations of Residents Living in Old Mining Areas.* Environmental Geochemistry and Health, 2004. **26**(1): p. 27-36.

437. Smith, E., J. Smith, and R. Naidu, *Distribution and nature of arsenic along former railway corridors of South Australia.* Science of the Total Environment, 2006. **363**(1-3): p. 175-182.

438. Pearce, D.C., K. Dowling, and M.R. Sim, *Cancer incidence and soil arsenic exposure in a historical gold mining area in Victoria, Australia: A geospatial analysis.* Journal of Exposure Science & Environmental Epidemiology, 2012. **22**(3): p. 248-257.

439. Conrad, S.R., et al., *Assessing pesticide, trace metal, and arsenic contamination in soils and dam sediments in a rapidly expanding horticultural area in Australia.* Environmental Geochemistry and Health, 2021. **43**(8): p. 3189-3211.

440. Jiménez-Oyola, S., et al., *Probabilistic multi-pathway human health risk assessment due to heavy metal (loid) s in a traditional gold mining area in Ecuador.* Ecotoxicology and Environmental Safety, 2021. **224**: p. 112629.

441. Papastergios, G., et al., *Arsenic background concentrations in surface soils of Kavala area, northern Greece.* Water, Air, & Soil Pollution, 2010. **209**: p. 323-331.

442. Sofianska, E. and K. Michailidis, *Chemical assessment and fractionation of some heavy metals and arsenic in agricultural soils of the mining affected Drama plain, Macedonia, northern Greece.* Environmental Monitoring and Assessment, 2015. **187**: p. 1-16.

443. Kampouroglou, E.E. and M. Economou-Eliopoulos, *Assessment of arsenic and associated metals in the soil-plant-water system in Neogene basins of Attica, Greece.* Catena, 2017. **150**: p. 206-222.

444. Farooqi, A., et al., *Sources of arsenic and fluoride in highly contaminated soils causing groundwater contamination in Punjab, Pakistan.* Archives of environmental contamination and toxicology, 2009. **56**: p. 693-706.

445. Sheik, C.S., et al., *Exposure of Soil Microbial Communities to Chromium and Arsenic Alters Their Diversity and Structure.* PLOS ONE, 2012. **7**(6): p. e40059.

446. Masood, N., A. Farooqi, and M.I. Zafar, *Health risk assessment of arsenic and other potentially toxic elements in drinking water from an industrial zone of Gujrat, Pakistan: a case study.* Environmental monitoring and assessment, 2019. **191**: p. 1-15.

447. Shehzad, M.T., et al., *Arsenic concentrations in soil, water, and rice grains of rice-growing areas of Punjab, Pakistan: multivariate statistical analysis.* Environmental Monitoring and Assessment, 2022. **194**(5): p. 346.

448. CoŞKun, M., et al., *Heavy metal pollution of surface soil in the Thrace region, Turkey.* Environmental monitoring and assessment, 2006. **119**: p. 545-556.

449. Yaylalı-Abanuz, G., *Heavy metal contamination of surface soil around Gebze industrial area, Turkey.* Microchemical Journal, 2011. **99**(1): p. 82-92.

450. Çolak, M., *Heavy metal concentrations in sultana-cultivation soils and sultana raisins from Manisa (Turkey).* Environmental earth sciences, 2012. **67**: p. 695-712.

451. Özkul, C., *Heavy metal contamination in soils around the Tunçbilek thermal power plant (Kütahya, Turkey).* Environmental monitoring and assessment, 2016. **188**: p. 1-12.

452. Varol, M., K. Gündüz, and M.R. Sünbül, *Pollution status, potential sources and health risk assessment of arsenic and trace metals in agricultural soils: A case study in Malatya province, Turkey.* Environmental Research, 2021. **202**: p. 111806.

453. Zhai, M., et al., *Heavy metal distribution in soils near Palapye, Botswana: an evaluation of the environmental impact of coal mining and combustion on soils in a semi-arid region.* Environmental Geochemistry and Health, 2009. **31**(6): p. 759-777.

454. Anawar, H.M., et al., *Arsenic contamination in groundwater of Bangladesh: perspectives on geochemical, microbial and anthropogenic issues.* Water, 2011. **3**(4): p. 1050-1076.

455. Manyiwa, T., et al., *Heavy metals in soil, plants, and associated risk on grazing ruminants in the vicinity of Cu–Ni mine in Selebi-Phikwe, Botswana.* Environmental Geochemistry and Health, 2021: p. 1-16.

456. Aschale, M., et al., *Pollution Assessment of Toxic and Potentially Toxic Elements in Agricultural Soils of the City Addis Ababa, Ethiopia.* Bulletin of Environmental Contamination and Toxicology, 2017. **98**(2): p. 234-243.

457. Bayissa, L.D. and H.R. Gebeyehu, *Vegetables contamination by heavy metals and associated health risk to the population in Koka area of central Ethiopia.* PloS one, 2021. **16**(7): p. e0254236.

458. Fernández-Martínez, R., et al., *Monitoring of mercury and other metals mobility by sequential fractionation in soils nearby an abandoned chlor-alkali plant in Managua (Nicaragua).* Environmental Earth Sciences, 2016. **75**: p. 1-12.

459. Carracelas, G., et al., *Irrigation management and variety effects on rice grain arsenic levels in Uruguay.* Journal of Agriculture and Food Research, 2019. **1**: p. 100008.

460. Säumel, I., et al., *Back to the future? Conservative grassland management can preserve soil health in the changing landscapes of Uruguay.* Soil, 2023. **9**(2): p. 425-442.

461. Žužul, S., et al., *Arsenic in air and soil in the vicinity of the central gas station Molve, Croatia.* Bulletin of environmental contamination and toxicology, 2011. **86**: p. 501-505.

462. Vidosavljević, D., et al., *Soil contamination as a possible long-term consequence of war in Croatia.* Acta Agriculturae Scandinavica, Section B–Soil & Plant Science, 2013. **63**(4): p. 322-329.

463. Kisić, I., et al., *The origin of arsenic in soils and groundwater of the Pannonian part of Croatia.* The holistic approach to environment, 2018. **8**(1): p. 23-36.

464. Bogunovic, I., et al., *Agricultural Soil Degradation in Croatia*, in *Impact of Agriculture on Soil Degradation II: A European Perspective*. 2022, Springer. p. 1-34.
